# Critical Evaluation of Gene Expression Changes in Human Tissues in Response to Supplementation with Dietary Bioactive Compounds: Moving Towards Better-Quality Studies

**DOI:** 10.3390/nu10070807

**Published:** 2018-06-22

**Authors:** Biljana Pokimica, María-Teresa García-Conesa

**Affiliations:** 1 Center of Research Excellence in Nutrition and Metabolism, Institute for Medical Research, University of Belgrade, Belgrade 11000, Serbia; biljana.pokimica@hotmail.com; 2 Research Group on Quality, Safety and Bioactivity of Plant Foods, Campus de Espinardo, Centro de Edafologia y Biologia Aplicada del Segura-Consejo Superior de Investigaciones Científicas (CEBAS-CSIC), P.O. Box 164, 30100 Murcia, Spain

**Keywords:** health effects, plant food bioactives, mRNA levels, RT-qPCR, human tissues, interindividual variability

## Abstract

Pre-clinical cell and animal nutrigenomic studies have long suggested the modulation of the transcription of multiple gene targets in cells and tissues as a potential molecular mechanism of action underlying the beneficial effects attributed to plant-derived bioactive compounds. To try to demonstrate these molecular effects in humans, a considerable number of clinical trials have now explored the changes in the expression levels of selected genes in various human cell and tissue samples following intervention with different dietary sources of bioactive compounds. In this review, we have compiled a total of 75 human studies exploring gene expression changes using quantitative reverse transcription PCR (RT-qPCR). We have critically appraised the study design and methodology used as well as the gene expression results reported. We herein pinpoint some of the main drawbacks and gaps in the experimental strategies applied, as well as the high interindividual variability of the results and the limited evidence supporting some of the investigated genes as potential responsive targets. We reinforce the need to apply normalized procedures and follow well-established methodological guidelines in future studies in order to achieve improved and reliable results that would allow for more relevant and biologically meaningful results.

## 1. Introduction

**Figure 1:**
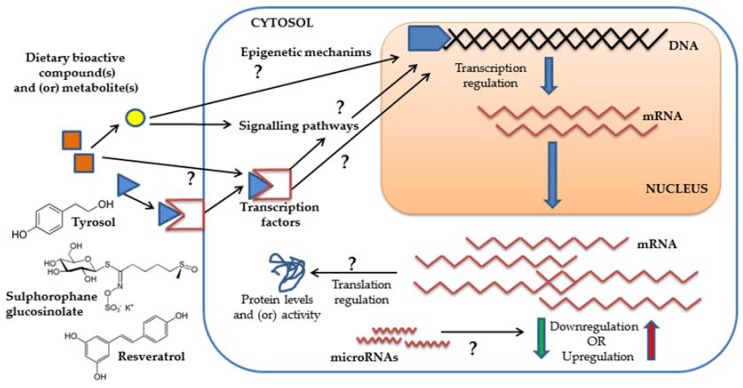
Scheme summarizing some of the general potential molecular mechanisms triggered in cells by bioactive compounds and/or derived metabolites and that may promote gene expression changes.

Plant foods and many of their derived products (beverages, oils, extracts) contain a variety of essential and non-essential bioactive compounds (mostly fatty acids and phytochemicals) which have been endorsed with a variety of biological activities that can contribute to the promotion of a healthy status and/or the prevention of various chronic and inflammatory disorders (e.g., cancer, obesity, cardiovascular and neurological diseases) [1,2]. A large number of pre-clinical studies have repeatedly shown that exposure of human cultured cells or animal models to some of these bioactive compounds and/or some of their derived metabolites is associated with changes in the levels of a wide array of molecular targets which suggest that this might constitute one of the mechanisms of action by which dietary bioactive compounds exert their beneficial effects [3]. Many of these compounds have been shown to exert anti-proliferative [4], anti-inflammatory [5] and anti-obesity [6] effects as well as cardiovascular [7] and neurological [8] protection, in association with changes in the expression of genes implicated in cancer development (*TP53*, *CTNNB1*, *MYC*, *MMPs*), apoptosis (*CASPs*, *BAX*, *BCL2*), cell cycle control (*CDKN1A*, *CDKN2A*), transcription (*NFKB*, *AKTs*, *STATs*, *NFE2L2*, *JUN*), cell adhesion (*VCAM1*, *ICAM1*), inflammation (*TNF*, *ILs*, *NOS2*, *PTGS2*, *CCL2*), xenobiotic metabolism (*CYPs*, *UGTs*, *SULTs*), energy metabolism (*PRKAs (AMPK)*, *PPARs*, *PPARGC1A*, *UCPs*), transporters (*SLC2A4*), and/or redox processes (*NOS2*, *GPXs*, *GSTs*, *SODs*, *CAT*) to mention but a few examples. Many of these molecular targets are involved in multiple cellular processes and appear to be commonly responsive to different compounds and thus, different bioactives or bioactive-containing products appear to exert their anti-inflammatory effects by downregulating the expression of pro-inflammatory cytokines such as *TNF*, *IL1B* and *IL6*, to reduce weight gain by altering key metabolic regulators like *PRKAs (AMPK)*, *PPARs* or *UCPs* or to exert antioxidant effects by the modulation of key targets such as *HMOX1* or *GPX* (extensive reviews on the regulation of genes by plant food bioactives in relation with chronic disorders can be seen at [1,2,3,4,5,6,7,8]. It has been proposed that the molecular responses to the bioactive compounds may be universal and present a high level of complexity given the large number of different molecules that can be modulated in the different types of cells and tissues within the body [9]. The specific mechanisms by which the dietary bioactive compounds or their derived metabolites may affect the different molecular targets have not yet been elucidated. Many of the molecular target changes have been shown at the level of gene expression and thus, it has been postulated that the effects of the bioactive compounds may be mediated by the induction or repression of gene expression *via* direct molecular interaction (signaling pathways, transcription factors) or, most likely, by epigenetic mechanisms (DNA methylation, histone modifications, microRNAS or LncRNAs) [9,10] (Figure 1). 

Another crucial issue that remains unresolved is the verification in humans of the proposed molecular responses to the intake of these compounds. An increasing number of human intervention trials have attempted to explore the effects of the consumption of different bioactive compounds or bioactive-containing foods or products on gene expression in different human tissues but, the results are still very limited. Despite the guiding principles proposed by the MIQE guidelines [11] to improve the general quality of gene expression biomedical research, many errors such as inappropriate study design and sampling protocols and gene expression data analysis-related incorrectness continue to be reported [12]. The validation of the involvement of specific gene targets and molecular mechanisms in the response to specific bioactive compounds or metabolites poses an extra level of difficulty in human dietary intervention studies that, at present, is far from being resolved. In this review, we have compiled a selection of human intervention trials in which the sample population was challenged with a source of bioactive compound(s), i.e., diets, foods or food-derived products (extracts, drinks, oils) and in which gene expression responses were investigated in different cells and tissue samples using RT-qPCR, the gold standard for accurate and sensitive measurement of gene expression [13]. Our main aims were to: (1) critically appraise the experimental design, methodology and gene expression data analysis applied in these studies; and (2) re-examine and discuss the accumulated evidence and relevance of the attained results. We herein reinforce the urge to follow general normalized procedures to improve the quality of future nutrigenomic studies so that we can collect most reliable gene expression responses to food bioactive compounds in humans and provide better evidence of the molecular targets and mechanisms underlying the beneficial effects of these compounds.

## 2. Literature Searching, Studies Selection and Data Extraction

This review has collected human clinical trials in which the test group of volunteers was administered with different dietary sources of bioactive compounds and where potential gene expression changes were investigated in human cells or tissues using RT-qPCR in response to the intervention. A search of the literature was undertaken using the PubMed database (last date assessed February 2018). Several key words were used in combination: (human OR humans OR patient OR patients OR volunteer* OR participant* OR subject* OR male OR males OR female OR females OR men OR women) AND (gene regulation OR nutrigenomic* OR gene OR genes OR gene expression OR transcription OR transcriptomic* OR mRNA OR messenger RNA OR RT-PCR OR real-time PCR OR PCR-arrays) AND (plant bioactive* OR polyphenol* OR phenolic* OR flavonoid* OR carotenoid* OR phytosterol* OR glucosinolate* OR fatty acids).

Human trials performing only microarray analyses for gene expression changes in response to dietary bioactive compounds were not specifically searched for and, therefore, not included in this review because of the broad diversity of analytical strategies, the complexity and large differences in the data reporting, and the still requested validation of the results by RT-qPCR. This type of studies deserves a complete separate revision. After we excluded some manuscripts reporting only microarray results, we revised and compiled information from 75 human intervention trials published between 1994 and 2018 (supplementary Table S1) [4,14,15,16,17,18,19,20,21,22,23,24,25,26,27,28,29,30,31,32,33,34,35,36,37,38,39,40,41,42,43,44,45,46,47,48,49,50,51,52,53,54,55,56,57,58,59,60,61,62,63,64,65,66,67,68,69,70,71,72,73,74,75,76,77,78,79,80,81,82,83,84,85,86,87]. Data extraction was conducted by two independent reviewers (BP and MTGC) and distributed in Supplementary Tables S1–S3. We classified the trials by intervention type and recorded the type of participants and trial design; involved groups (control, treatment); dose and duration; and associated bioavailability studies (Supplementary Table S1). We then assessed the reporting of several critical methodological details: description, preparation and characterization of the biological samples; experimental protocols applied for RNA isolation and characterization, and the selection of the reference gene(s) for data normalization (Supplementary Table S2). We finally recorded the gene expression changes reported in the manuscripts as potentially attributed to the interventions, the type of comparisons and sample size for each group compared, as well as the data analysis and presentation. We additionally searched for information about the potential association of the gene expression results with the presence of bioactive compounds and/or derived metabolites detected in the body after the intervention and/or with potential confirmatory changes at the protein level (Supplementary Table S3).

## 3. Summary of the Experimental Designs of the Intervention Trials Examined in this Review

Randomized controlled trials (RCTs) constitute one of the most powerful tools of research to evaluate the effectiveness of intervention with a drug or a dietary compound such as bioactive compounds on human health [88]. Of the studies included in this review (Supplementary Table S1) a considerable proportion (76%) was randomized with ~60% designed in parallel groups and ~39% in crossover. About 65% of these randomized studies were also blinded, most commonly (84%) double blinded, indicating that a good proportion of the trials follow an appropriate design. A few parallel or crossover studies did not report randomization and/or blinding. Regarding the sex of the participants, 59% of the studies were conducted in mixed cohorts of men and women, 33% in groups of men and only 8% in women. As for the health status, nearly 60% of the studies were carried out in healthy volunteers including some healthy obese people, smokers and sportive individuals. Among the disease groups, gene expression studies were carried out in patients with various disorders such as metabolic syndrome (MetS), diabetes, hypertension, obesity, risk of cardiovascular disease (CVD), diverse types of cancer (oral, colon, prostate, leukemia) and various other inflammatory diseases (chronic gastritis, photo-dermatosis, sickle cell disease, Friedrich ataxia, non-alcoholic fatty liver disease, cystic fibrosis).

We have collected a total of 75 intervention studies looking at the effects on gene expression of the intake of a variety of sources of bioactive compounds, from complex mixtures such as mix meals and diets (fruit + vegetable diets, Mediterranean diets), foods and derived food products (broccoli, oils, nuts, beverages, onions, fermented papaya, propolis), and extracts and supplements (grape, berry and pomegranate extracts, plant extracts, oil derived extracts and compounds, mixed supplements), to single compounds such as β-carotene (β-car), eicosapentaenoic acid (EPA), docosahexaenoic acid (DHA), resveratrol (Res), quercetin (Quer), flavopiridol, genistein, epigallocatechin gallate (EGCG), and curcumin (Cur). The duration of the interventions ranged from a few days up to one year but most studies were carried out in periods between one and 3–4 months. Approximately 15% of the studies were classified as acute or postprandial since gene expression was measured within 3–24 h of the intake. The doses of the test compounds/products were very variable and fluctuated between a few mg to g quantities. For example, the doses employed with single bioactive compounds varied between 30 mg of β-car [71], Quer [79], or genistein [83], up to nearly 3 g of EPA/DHA [72] or Res [76] and even up to 12 g of Cur [87]. Comparisons between several doses were also investigated in several studies (*n* = 19) and in 3 studies the exact daily dose of the treatment was not reported. 

One of the most critical points in a clinical trial design is the use of appropriate control groups and one of the best approaches should be the use of a matched placebo in which the control group would consume an almost identical test product containing all the constituents except for the bioactive ingredient [89]. The selection of a suitable control group is particularly complicated in trials with dietary interventions as evidenced by the variety of comparative strategies applied in the different studies included in this review. There were a considerable number of single-arm studies that did not include comparison to a control group and the changes in gene expression were only reported between after (post-) and before (pre-) intervention. Other trials estimated gene expression differences: (i) between low vs. high doses of the test bioactive compound(s), as in several studies looking at the effects in gene expression of the bioactive compounds present in the olive oil [27,28,30,31,34,35] or those present in broccoli sprouts [21,26]; (ii) between different diets [18,19] or food products [22,32,33,55]; and (iii) comparison to a placebo group [15,49,50,51] not always well described. 

Around 50% of the trials included the study of the appearance and/or changes in urine, plasma and/or in some tissues of the levels of different compounds and metabolites that were potentially derived from the intake of the test product, i.e., bioavailability studies. For example, the intake of mix fruit and vegetable was found to be associated with changes in the levels of carotenoids in plasma and of flavonoids in urine [15,16,17], the intake of broccoli with increases of sulphoraphane metabolites in urine and plasma [21,22,25], the intake of olive oil products with changes in tyrosol (Tyr) and derived metabolites (hydroxytyrosol, HTyr) in urine and plasma [28,30,31,34,35], or the presence of urolithins in prostate [37] and colon [4] tissues with the intake of walnuts and pomegranate. Some of the trials carried out with single compounds also reported the appearance of β-car [71], EPA/DHA [72], Res [73,74,76,77], Quer [80,81] or EGCG [85]. Of note, a few trials reported a high interindividual variability in the presence of the bioactive compounds and/or metabolites in the biological samples examined [4,37,76].

## 4. Overview of Some Critical Issues Related to the RT-qPCR Experimental Protocols Used in the Intervention Trials

RT-qPCR remains a most sensitive and reliable technique that can specifically measure small to moderate changes in mRNA levels in cells and tissues in response to environmental challenges [13] such as those promoted by dietary supplementation with bioactive compounds and/or foods and food products enriched in these compounds. Supplementary Table S2 collects information about some of the most critical points relative to the RT-qPCR experimental protocols form all the human intervention studies gathered in this review, i.e., type of samples (cells or tissues) and preparation, RNA extraction protocols and quantity/quality assessment as well as the house keeping gene(s) used as a reference for data normalization.

### 4.1. Sample Characterization and Handling

The majority of the intervention studies included in this review used RNA extracted from whole blood (10 studies) or from blood isolated immune cells (40 studies), principally from peripheral blood mononuclear cells (55%), as the target tissues in which to measure gene expression changes that might be attributed to the intervention with the test products containing bioactive compounds. Also, isolated lymphocytes were the target tissue in seven studies [14,38,42,43,44,60,79], and monocytes, polymorphonuclear granulocytes and neutrophils were used in one or two studies [23,45,60,81]. A total of 10 studies reported gene expression changes in tissue samples from the gastrointestinal tract (oral, stomach, small intestine and colon samples) [4,21,47,48,52,54,61,66,71,86]. Other additional internal tissue samples used in humans were biopsies from prostate [29,37,83], adipose tissue [36,73,74,85], skeletal muscle tissue [73,74,75,80,84] and skin samples [46,59,68,69]. One study looked at gene expression changes in cells present in nasal lavage [22]. In general, and despite the fact that all these blood cells and tissue samples are heterogeneous and constituted by different types of cells, most of the studies examined here did not report a complete blood count and blood cell proportions or some description of the cell types and composition present in any of the investigated tissues. Only one study, reported the proportion of immune cells forming the isolated mononuclear cell samples showing moderate interindividual variability in the cell composition: lymphocytes (84.4 ± 2.9%), monocytes (13.0 ± 3.2%) and granulocytes (2.2 ± 1.4%) [51]. A few other studies reported mononuclear cells purity (>95%) [58], isolated blast cells enrichment (>70%) [82], or neutrophils enrichment and viability (>90% cells, 90% viable) [45]. In the biopsies, classification between cancer and non-cancerous tissue samples was indicated to be carried out by specialized pathologists but no further description of the cell composition within the samples was included [4,37]. Only one study used laser capture microdissection for tissue sampling attaining a specific number of cells (10,000) from benign and from normal tissues [83].

Sample handling and preparation protocols also contribute to the experimental variability and thus, it is important to report in as much detail as possible how the samples were obtained and processed until use [11]. The revision of this part of the experimental protocol in all the selected articles evidenced a general poor or limited description of the procedure used to obtain the samples as well as about the processing time, the sample storage conditions and the time elapsed until RNA extraction. There is a broad variation in the sampling procedures used or the information reported with a considerable number of studies including few details. For example, for the gene expression studies carried out in whole blood, only half of them specifically reported the collection of fasting blood samples. Also, only six of these studies applied a specific commercial kit for direct RNA isolation from blood whereas the rest of the studies did not provide any information about the protocol used. In those cases where the storage was reported, the most common way of long term sample storing was at −80 °C (as shown in ~40% of the studies). However, the time elapsed before storage was not reported in many of the studies and, in those where it was indicated, it varied from quick-freezing in liquid N_2_ to maintenance and/or transport at room/low temperature for several hours before freezing. The preservation solution varied between studies (RNAlater or lysis buffer were most commonly used, but other buffers, DEPC-treated solutions, or even specific cell culture medium were also used). Further, and importantly, there was no reporting of the storage time period prior to the RNA extraction except for one study that indicated to have done gene expression analyses within one month of storage [61]. 

### 4.2. RNA Extraction Protocols, Quantity and Quality

RNA extraction, quantitation and integrity inspection are also critical steps that can influence the gene expression changes. The use of different RNA extraction protocols may account for some of the differences in gene expression results and various factors can affect the final quantity and quality of the isolated RNA (i.e., degradation by ubiquitous RNases, co-extraction of inhibitors, presence of contaminant DNA and/or protein) potentially compromising the results of the RT-qPCR analysis [12]. This is especially critical if we want to compare results from different studies. In this review, most of the studies indicated to have isolated the RNA using one of the many different commercially available extraction kits including mostly those based on affinity columns or those using the Trizol reagent and liquid-liquid extraction. Nevertheless, about 17% of the studies did not describe clearly the protocol used. In addition, of all the studies included, only 12 studies (16%) specified to have applied a DNase treatment in the procedure to remove contaminating genomic DNA. 

Reliable analysis of changes in the mRNA levels requires that the extracted RNA is of high quality and that both the quantity and integrity is accurately determined [90]. This is especially relevant in human clinical studies with usually limited and unique tissue samples, however, not many publications report RNA yield and/or quality values [12]. In this review, most of the selected studies that indicated to have measured the quantity of extracted RNA, reported to have done so by spectrophotometry (Abs 260 nm) using the NanoDrop system in many cases. Only in one study they used the Ribogreen method [44]. Nevertheless, none of the studies presented information about the RNA yield obtained from the specific cells or tissues analyzed except for one study where they indicated an average RNA yield of 0.3–1.0 μg extracted from nasal lavage samples [22]. 

Of the 75 clinical studies included in this review, we found that 66 publications (88%) did not report any quality score of the RNA samples used in the gene expression studies even though, a considerable number of these studies reported to have assessed the quality of the RNA, either by spectrophotometry determination of the Abs_260/280_ (mostly using the NanoDrop system) or by agarose gel and examination of the 18S and 28S bands (often using the Bioanalyzer capillary chips). Of those studies that included information about the RNA quality, two of them only mentioned that some of the samples had low quality [15,82] and four studies reported the quality of the ARN based solely on the Abs_260/280_ with values between 1.8 and 2.1 [37,67,74,75]. Two studies reported the RNA integrity number (RIN) values of their samples, RIN > 8.0 [64] and RIN = 6–9 [54], respectively, and only two other studies combined Abs_260/280_ and RIN values [4,51] to prove the quality of their RNA samples.

### 4.3. Reference Genes

The selection of genes stably expressed to be used as endogenous or reference genes remains the most common method for mRNA data normalization and a critical issue in RT-qPCR studies, especially when working with heterogeneous tissue samples. The incorrect use of reference genes can have a profound impact on the results of the study [91]. Table 1 displays the list of reference genes, with the most updated nomenclature available from GeneCards [92], used in the intervention studies included in this review. The majority of these studies used a single gene as a reference with *GAPDH* being the most commonly one applied, followed by *ACTB* and *18S rRNA*. The rest of the studies applied other common but yet less frequently used genes such as *B2M*, *HPRT1*, *GUSB*, or ribosomal proteins. There were also two studies in which normalization was carried out using the gene *AW109* [62], an RNA competitor [93], and single stranded DNA (*ssDNA*) [75], respectively, as references. As for the type of samples in which these genes were used for normalization, the three top reference genes, *GAPDH*, *ACTB* and *18S rRNA*, were indistinctively used in a wide range of cell types and tissue samples, i.e., whole blood, mononuclear cells, other various isolated immune circulating cells (lymphocytes, monocytes, neutrophils), gastrointestinal biopsies, prostate biopsies, skeletal muscle, adipose tissue and skin. On the other hand, in mononuclear cells (the primary type of cells used in the gene expression human studies) most of the listed genes (*GAPDH*, *ACTB*, *18SrRNA*, *HPRT1*, *B2M*, *UBC*, *PPIA*, *HMBS*, *YWHAZ*, *RPL13A*, *RPLP0*) were used as reference genes.

Only a few studies indicated to have used the average value or the combination of various reference genes (*GAPDH*, *ACTB*, *HPRT1*, *B2M*) [18,40], (*GAPDH*, *ACTB*) [64] and (*ACTB*, *UBC*, *PPIA*) [58] for the normalization process whereas others reported to have used separate reference genes (*RPLP0*, *ACTB*, *B2M*) [82] and (*GAPDH*, *HMBS*) [45] for the analysis of expression changes of different target genes within the same study. A total of seven studies did not clearly report the use of or the specific reference gene employed in their analyses. Regarding the criteria employed to choose the reference gene(s), very few studies indicated to have taken into consideration either information from the literature [66], the RefGenes tool in the curated expression database Genevestigator [38] or to have determined the stability of the genes using specific tools such as GeNorm or Normfinder [4,76]. 

### 4.4. Gene Expression Data Reporting

Supplementary Table S3 compiles information about the different genes that were investigated in each of the trials included in this review. In general, and regarding the sample size, most gene expression analyses were carried out using a small number of individuals per group (generally <25 and, frequently, the control and treated groups were integrated by less than 10–15 individuals). Table S3 includes the list of genes that were found upregulated (↑, red color) or downregulated (↓ green color) in response to each intervention. When available, we added the significance of the change (*p*-value) and the specific comparison in which the change was detected. We also annotated those genes that exhibited a not significant change and those that were reported not to be affected with the treatment. This information specifically shows the variety of comparative strategies and presentation of results (gene expression change) used in the different studies. Differential expression was reported for some genes as: (i) the difference between after (post-) and before (pre-) intervention (↑*HMOX1* in blood after intervention with 150 g of broccoli [26]); (ii) the difference between the treated and the control group only at the end of the intervention period (↑*PPARA* in blood in a group consuming EPA or DHA against a control group, [72]). There were various other comparative approaches such as between different doses of the bioactive compounds (↑*PPARA* in white blood cells when comparing olive oil with a high level of polyphenols vs. olive oil with a moderate dose of polyphenols [31]), between different diets (↓*UCP2* in blood with tocopherol-enriched Mediterranean diet vs. a Western high-fat diet [19] or between different food products (↑*HMOX1* in cells from nasal lavage after intervention with 200 g of broccoli sprouts vs. 200 g of alfalfa sprouts [22]). 

For the estimation of the gene expression changes, the majority of the trials reviewed here used relative expression quantification applying the Ct comparative method based on a standard curve, normalized Ct values (using a reference gen) and the 2^(−∆∆Ct)^ formula [94]. Nevertheless, the presentation of the results (expression changes) also varied a lot between the studies. The changes in the genes were reported either as relative expression levels in the different groups (occasionally as arbitrary units or as the number of copies of mRNA), as a change (typically as FC or ratio but also as the % of change) or as a combination of both within the same report. The final results were typically presented using a statistical approach to describe the central tendency and the dispersion of the results but there was also a high variability in the estimators used in each study. Around 65% of the studies used the mean value as an estimator of the average changes but a few studies reported the median [4,14,43,51,66,76] or even the geometric mean of the data [31]. Regarding the dispersion of the results, most studies indicated either the SEM (~35%) or the SD (~29%) but some studies reported the confidence intervals [18,31,47,54,62,77,86] or the quartiles/ranges [4,14,37,42,43,51,66,76,82]. Overall, there was a considerable lack of clarity in the presentation of the results, many of which (~50%) were included only in figures not always well described. In some studies it was not trivial to infer the evidence supporting the changes. Between 23% and 24% of the studies failed to report or to correctly identify the measurement of the average change or the data variability. Of note, a few studies reported the distribution of gene expression changes in the sample population, e.g., % of individuals exhibiting downregulation, upregulation or no change for a particular gene [4,82,86] whereas in some other studies, individual gene expression changes were displayed [14,54,57].

### 4.5. Gene Expression Results: Association with Bioavailability of Bioactive Compounds and/or with Protein Confirmatory Studies

Of the trials collected in this review that reported the presence of certain compounds and metabolites in the blood, urine and/or tissue samples (bioavailability), only a few (17 studies) attempted to investigate the potential relationship between the gene expression changes detected and the appearance and/or concentration of the specific compounds and/or metabolites. Only 5 of those studies reported some positive or inverse correlations between the changes of the expression in some genes and the levels of some metabolites. For instance, the downregulation of the expression of *IFNG*, *OLR1* or *ICAM1* and the upregulation of *ABCA1* in peripheral blood cells in association with the increase of Tyr and/or HTyr metabolites in urine or plasma following the intake of olive oil rich in polyphenols [28,30,31]. With regards to the validation of the gene expression changes by further testing the levels of the corresponding proteins and/or protein activity, only 27 studies (36% of total) made an attempt to substantiate gene changes with protein changes. In those, ~50% of the examined gene changes resulted in some confirmatory protein changes, e.g., the downregulation of TNF in blood and the reduction of the plasma levels of TNF following the intake of a grape seed extract [49] or, the upregulation in mononuclear cells of *LDLR* and the increase in the levels of LDLR in monocytes and T-lymphocytes after the intake of mix plant stanol esters [62]. 

## 5. Gene Expression Changes in Human Cells and Tissues in Response to Food Bioactive Compounds: Overview of the Accumulated Evidence

### 5.1. Gene Expression Changes Specifically Reported in Blood Isolated Immune Cells in Response to Different Sources of Bioactive Compounds

Table 2 collects the genes reported to be significantly (*p*-value < 0.05) up- or downregulated in blood isolated immune cells after intervention with dietary foods or products containing bioactive compounds. The table indicates the type of cells where the study was carried out, the comparison in which the changes were detected and the potential bioactive compounds involved in the response. Most of the studies were carried out in peripheral blood mononuclear cells but there were some studies looking at isolated lymphocytes [14,34,42,43,79], neutrophils [45,60] or granulocytes [23]. Most trials were carried out with foods or products containing mixed bioactive compounds and only a few studies investigated the effects of isolated compounds, e.g., Quer [79,80] or EPA/DHA [65,67]. The table also indicates the biological processes and health effects investigated in each study in relation with the response to the intervention and to which the changing genes might be associated with, i.e., metabolism of lipids and carbohydrates and associated disorders (atherosclerosis, high blood pressure), regulation of the inflammatory status, and oxidative stress and antioxidant responses. The messages associated with the different interventions and with the changing genes were messages of improvement or beneficial effects. Some of the genes most commonly investigated in relation with these responses and that might be considered as targets responsive to bioactive compounds included well known transcriptional factors, cytokines, key metabolic regulators and antioxidant genes. For instance, some members of the *PPAR* family (*PPARG*) were upregulated in circulating blood immune cells in response to the intake of mixed bioactive polyphenols and fatty acids [31,67,70]. Also, some cytokines (*TNF*) and interleukins (*IL1β*, *IL6*) were found to be downregulated in this type of cells following intervention with different oils [27,67], fruit extracts [51] or nuts [38]. Regarding the transcription factors, *NFKB* and *EGR1* were downregulated in these cells after the intake of mixed omega-3 [65] or mixed olive oil bioactive compounds [27,64] whereas *NFE2L2* was found increased in response to the mixed compounds present in sunflower oil [32] and in coffee [42,43] but downregulated in response to the intake of a berry extract rich in anthocyanins [53]. Several genes related with the antioxidant status regulation (*SODs*, *CAT*, *GPX1*) also exhibited up- or downregulation in the immune cells with different interventions [32,42,45,60]. 

### 5.2. Accumulated Evidence for Specific Gene Targets in Response to Different Sources of Bioactive Compounds

We specifically looked at all the changes reported in different cells and tissues for some genes that might be considered targets responsive to bioactive compounds and that are associated with inflammation (*TNF*) (Supplementary Table S4), energy metabolism (*PPARs*) (Supplementary Table S5), and antioxidant effects (*GPXs*) (Supplementary Table S6). As already stated, data reporting was variable among the studies and the quality of the results was generally poor. Where possible and using the information available from the articles we estimated the coefficient of variation (CV) of the reported gene expression changes. Overall, between 40% and 60% of the studies reported a lack of effect of the intervention on the selected genes.

Main details and results from those other studies reporting significant outcomes for *TNF*, *PPARs* and *GPXs* expression changes are gathered in Table 3. Based on these results, it would appear that the intake of different mix bioactive compounds could be associated with the downregulation of *TNF* and the upregulation of some *PPARS* (*PPARA*, *PPARG*) and *GPXs* (*GPX1*) genes in blood and/or in isolated blood immune cells. There were, however, some opposed significant results such as the upregulation exhibited by *TNF* expression levels in blood in response to EPA/DHA [72]. Also, different members of the same family of genes may react differently as it was the case of the *GPX* genes in response to the intake of hazelnuts [40]. Regarding the effect size (gene expression changes) and, for comparative purposes, we estimated and converted all the results into FC-values and % of change. We observed a large variation in the changes reported ranging from very small FC-values, e.g., +1.06 (6%) for *PPARG* in mononuclear cells [67] or +1.07 (7%) for *TNF* in blood [72] to rather high changes, i.e., FC > +2.5 (>150%) such as those reported for some members of the *PPARs* and *GPXs* families [18,31,70]. As for the variability in these gene expression responses, in those studies for which we were able to estimate the CV, these CV values were high and variable. In some cases, the % of change attributed to the intervention was in the range of the estimated CV, e.g., the 11% reduction of *TNF* by fish oil EPA + DHA with an estimated variability of 10.5–18.0% [67]. In other studies, the CV was well above the % of change attributed to the intervention, e.g., a 100% increase in *PPARA* attributed to mix olive oil compounds with estimated CV values > 100% [31]. In addition, none of these studies reported the potential association between those gene expression changes and the presence or changes in the quantities of specific compounds or derived metabolites in the samples and, there was little or none evidence supporting the regulation of the corresponding encoded proteins. Overall, the level of evidence supporting the specific described gene changes as potential mechanisms of response to the intake of the bioactive compounds present in the test food products investigated remains very low. 

Main details and results from those other studies reporting significant outcomes for *TNF*, *PPARs* and *GPXs* expression changes are gathered in Table 3. Based on these results, it would appear that the intake of different mix bioactive compounds could be associated with the downregulation of *TNF* and the upregulation of some *PPARS* (*PPARA*, *PPARG*) and *GPXs* (*GPX1*) genes in blood and/or in isolated blood immune cells. There were, however, some opposed significant results such as the upregulation exhibited by *TNF* expression levels in blood in response to EPA/DHA [72]. Also, different members of the same family of genes may react differently as it was the case of the *GPX* genes in response to the intake of hazelnuts [40]. Regarding the effect size (gene expression changes) and, for comparative purposes, we estimated and converted all the results into FC-values and % of change. We observed a large variation in the changes reported ranging from very small FC-values, e.g., +1.06 (6%) for *PPARG* in mononuclear cells [67] or +1.07 (7%) for *TNF* in blood [72] to rather high changes, i.e., FC > +2.5 (>150%) such as those reported for some members of the *PPARs* and *GPXs* families [18,31,70]. As for the variability in these gene expression responses, in those studies for which we were able to estimate the CV, these CV values were high and variable. In some cases, the % of change attributed to the intervention was in the range of the estimated CV, e.g., the 11% reduction of *TNF* by fish oil EPA + DHA with an estimated variability of 10.5–18.0% [67]. In other studies, the CV was well above the % of change attributed to the intervention, e.g., a 100% increase in *PPARA* attributed to mix olive oil compounds with estimated CV values > 100% [31]. In addition, none of these studies reported the potential association between those gene expression changes and the presence or changes in the quantities of specific compounds or derived metabolites in the samples and, there was little or none evidence supporting the regulation of the corresponding encoded proteins. Overall, the level of evidence supporting the specific described gene changes as potential mechanisms of response to the intake of the bioactive compounds present in the test food products investigated remains very low. 

### 5.3. Summary of the Effects on Gene Expression of Specific Food Products Containing Bioactive Compounds

Table 4 gathers the reported changes on gene expression following the consumption of three of the most investigated plant derived foods and products containing different families of bioactives, i.e., olive oil and extracts rich in polyphenols, mostly focused on Tyr and HTyr [27,28,30,31,34,35,36,63,64]; broccoli sprouts and derived products rich in sulphoraphane glucosinolates (SFGluc) [21,22,23,24,25,26]; and grape extracts containing polyphenols, mainly with a focus on Res [48,49,50,51,73,74,75,76,77,78]. Most of these gene expression changes were investigated in blood or peripheral blood immune cells except for a few studies conducted in adipose and/or skeletal tissue, and in samples from the gastrointestinal tract. 

A total of eight clinical trials that investigated the effects of the consumption of olive oil products containing bioactive compounds (polyphenols) were included in this review. These studies focused on the potential benefits of olive oil bioactives on energy metabolism, such as the regulation of the levels of cholesterol [31,34], and on inflammatory related processes [27,30,64] as well as in the concurrent regulation of the expression levels of specific genes related with these processes. The accumulated evidence to support any of these genes as specific gene targets responsive to the intake of the olive bioactives is still very limited although some of those genes display preliminary consistent changes following the consumption of these products. For example, the gene *ADRB2* was found to be downregulated in mononuclear cells in three separate trials comparing the intake of olive oil containing high vs. low doses of polyphenols [28,30,35]. Also, the transcription factor *EGR1* which regulates the transcription of numerous inflammatory related interleukins and cytokines [95], or the *IL7R* were found downregulated in mononuclear cells in response to high-polyphenol olive oil and olive leaf extract [27,28,30,64] supporting the anti-inflammatory effects attributed to these products. On the other hand, the changes in the expression levels reported for other targets such as the chemokine *CCL2* were found to be reduced in mononuclear cells following 21 days of intervention with olive oil polyphenols [30] as opposed to the induction reported in adipose tissue after 4 h of the intake of mix olive oil bioactive compounds or the lack of effect detected in the same adipose tissue after 28 days consuming the olive oil bioactive compounds [36]. *PTGS2* (also designated as *COX2* and critically involved in the anti-inflammatory response) was found downregulated following intervention with olive leaf polyphenols in mononuclear cells [64] but no significant change was detected in studies conducted with olive oil polyphenols [27,31]. Regarding the specific bioactive compounds present in the olive products, Tyr and derived metabolites (HTyr) have been investigated and reported as some of the molecules potentially responsible for some of the gene expression effects observed in humans. The upregulation of the membrane transporter *ABCA1* has been related with the increase of the levels of HTyr in plasma [31], or the downregulation of *IFNG* or *OLR1* have been associated with the increase of Tyr in urine [28,30]. 

Among the six intervention studies conducted with broccoli, the richest source of SFGluc, five trials examined and reported the effects of this type of product in the expression of *HMOX1*. The results were diverse with three studies giving significant evidence of the upregulation of this gene in blood [26], in polymorphonuclear granulocytes [23], and in nasal lavage cells [22] whereas in the remaining two studies, no change was detected in blood [25] or in mononuclear cells [24]. In all these intervention trials, the main bioactive compound to which the effects might be potentially attributed to was the SFGluc but, we found no evidence of the presence of SFGluc metabolites in the biological samples in association with the specific gene responses. The overall accumulated evidence in humans of the gene regulatory effects of SFGluc from broccoli remains limited.

The last group of bioactive-containing products gathered in Table 4 refers to studies conducted with grape derived powders and extracts, to which anti-inflammatory, antioxidant and metabolic benefits have also been attributed to. Some of the gene expression changes reported in humans after intervention with these grape extracts shows downregulation effects in various interleukins and in the multifunctional pro-inflammatory cytokine *TNF* [49,51]. In this latter study, the downregulation of *TNF* has been associated with the presence of Res in the grape extract [51]. Grape products are characterized by containing mix flavonoids, but it has received special attention for the presence of Res, a widely investigated stilbenoid with many reported beneficial effects [96]. We have also gathered in Table 4 several human trials in which the test product was the single compound Res. None of these studies reported significant gene expression changes except for the downregulation of the glucose transporter *SLC2A4* in muscle tissue [74]. Of note, two studies reported the absence of effect on *TNF* in skeletal muscle [75] and in adipose tissue [74]. Equally, two other studies reported the lack of effect on the expression levels of the deacetylase *SIRT1* [73,78], a well-established target of Res in pre-clinical models [97].

## 6. General Discussion

In this review, we have thoroughly examined 75 human clinical intervention studies published in the past 20 years that investigated the health benefits of the intake of different bioactive compounds or of foods or food products containing these compounds, and that included the study of the changes promoted in specific gene targets in different cells and tissues using RT-qPCR. In general, there was a poor quality in the design and description of the experimental protocols as well as in the analysis and reporting of the gene expression changes posing doubts about the consistency and validity of the published results. A similar scenario was illustrated for biomedical research [12] reinforcing the need to apply the MIQE guidelines and well-established recommendations for RT-qPCR analyses [11,13] to enhance the quality of future gene expression studies. We have compiled in Table 5 some of the most critical experimental inaccuracies found in the human intervention studies reviewed here, in parallel with some general recommendations that should be necessarily implemented in order to produce more reliable gene expression results that could be truly attributed to the intake of dietary bioactive compounds. On the basis of this Table, we suggest the feasibility of establishing a quality score system that would define the validity and sufficiency of the human trials investigating gene expression responses to intervention with dietary bioactive compounds, with a maximum score for those studies implementing all the points proposed here, i.e., best study design, good sample population description (even at individuals levels), good placebo-control and intervention groups that allow for the attribution of the effects to the intake of specific compound(s), thorough application of the MIQE guidelines for the RT-qPCR analyses, good comparative strategy and clarity of results presentation, assessment of interindividual variability, parallel bioavailability studies that allow for potential association between gene expression changes and the presence of specific compound(s) and/or derived metabolite(s) in the analyzed biological samples, and last, but not least, potential confirmation of the gene changes at the level of protein amounts and/or activity. 

In spite of the limitations of the studies carried out so far, we have put together and revised the accumulated evidence for some specific target genes commonly investigated in different samples, especially in circulating immune cells, in response to different sources of bioactive compounds. Blood isolated immune cells, and in particular, mononuclear cells (lymphocytes and monocytes) have been used as surrogate cells in human gene expression studies since they are easy to obtain, are considered to reflect the metabolic and immune processes that may occur in other tissues, e.g., liver, adipose or skeletal muscle, but also, because of their own role in inflammation, obesity and other cardiometabolic disorders. Nevertheless, not many genes have been yet confirmed as responsive molecular candidates in relation with the effect of bioactive compounds against these diseases [98]. With regards of the studies carried out in blood isolated immune cells and included in this review, very few genes have been repeatedly shown to be significantly regulated after the intake of several foods and food products containing diverse bioactive compounds (Table 2). For instance, *PPARG*, a member of the subfamily of nuclear hormone receptors PPARs that regulate the expression of many genes, has been found commonly upregulated in blood immune cells following intervention with mixed olive oil polyphenols [31], with a mixture of polyphenols, fatty acids and vitamins [70] or with mixed EPA + DHA [67]. PPARG was originally described as a key regulator of lipid metabolism in the adipocytes but it is also expressed in most immune cells where it attenuates the expression of pro-inflammatory genes such as *IL1β*, *TNF* or *IFNG* and thus, the activation of *PPARG* constitutes also an important anti-inflammatory strategy [99]. In the previous studies, the intake of those bioactive compounds was associated with the reduction of triglycerides (TAGs) [31,67,70], oxLDL [31], and free fatty acids (FFAs) [70] as well as with the increase of LDL- and HDL-cholesterol [67] giving some evidence of regulatory effects in the lipid metabolism.

With regards to anti-inflammatory effects, only the intake of EPA + DHA was additionally associated with the reduction of the expression of *IL1β* and *TNF* [67] supporting the notion that these effects could be related with the induction of *PPARG*. Recently, it has been demonstrated in obese children and adolescents that the expression of *PPARG* is significantly decreased in peripheral blood mononuclear cells in comparison with gender-matched control subjects, and also negatively correlated with the levels of plasma fasting glucose, reinforcing that interventions to normalize *PPARG* expression may be an effective tool to reduce obesity and associated disorders [104]. Overall, the results reviewed here provide preliminary evidence that the intake of different foods containing bioactive compounds may be a potential strategy to activate *PPARG* in circulating immune cells and contribute to normalize the levels of plasma glucose or lipids. Whether this molecular event is the direct consequence of the ingested bioactive compounds or derived metabolites on these cells or a reflection of changes that may be happening in other tissues such as the liver or adipose tissue is not yet known. There may be also alternative mechanisms involved in this response such as the potential inhibition by the bioactive compounds of the digestion and absorption of energy nutrients (fats, carbohydrates) in the intestine [3]. In support of this hypothesis, it has been shown that the reduction of calorie intake affects the expression of *PPARG* in peripheral blood mononuclear cells in rats [105] and in the adipose tissue of obese women [106] reinforcing this gene as a valuable target of the regulatory metabolic effects of bioactive compounds that warrants further investigation.

Another gene potentially responsive to the intake of bioactive compounds and associated with their attributed anti-inflammatory effects is *TNF*. TNF is a primary mediator of inflammation critically involved in diverse chronic diseases and thus, any compound(s) with the capacity to modulate (reduce) this pro-inflammatory cytokine may constitute an important means to battle these disorders. Many plant derived polyphenols have been shown to inhibit or reduce the production of TNF in various in vitro cell models such as macrophages, monocytes, leukocytes or adipocytes as well as in animal models [107]. Nevertheless, the evidence in human studies remains limited. For example, Cur has been reported to reduce the levels of circulating TNF in a meta-analysis of 8 RCTs although there was no association with the dose or duration of the supplementation [108]. At the gene expression level, we have found only one study reporting the lack of effect of turmeric-containing Cur on the expression of *TNF* in the gastric antrum [86]. On the other hand, the expression levels of *TNF* have been reported to be significantly reduced in isolated mononuclear cells and in blood following intervention with various other foods and food products containing mixed bioactive compounds, i.e., hazelnuts (polyphenols and fatty acids) [40], grape products (flavanols and Res) [49,51], and with EPA + DHA [67] (Table 3). None of these studies, however, described the potential association between the downregulation of the gene and the specific presence and quantities of bioavailable bioactive compounds or derived metabolites in the cell and blood samples. In one study, the downregulation of *TNF* was specifically associated with the presence of Res in the grape extracts administered to the participants [51]. Res has been widely investigated for its involvement in the modulation of the inflammatory response and the cell-specific regulation of gene expression including the downregulation of *TNF* and other cytokines in leukocytes [109]. We collected a total of six human studies investigating the effect of Res on gene expression in different cell and tissues (Table 4) but only two of them included *TNF* as a target and both reported the lack of change in the expression levels of this gene in skeletal and adipose tissue, respectively [74,75]. The evidence in humans supporting *TNF* expression as a target gene for Res remains low. A similar situation was observed for *SIRT1*, a well-established target reported to be activated by Res in many pre-clinical studies [97]. The protein levels of SIRT1 were found increased in the muscle of obese volunteers after the intake of Res for 30 days but the authors did not report specific changes at the gene expression level [110]. Of the studies collected here, the expression levels of *SIRT1* in skeletal muscle [73] and in white blood cells [78] did not appear to be induced after the intake of Res. 

The health-promoting effects of many plant-derived bioactive compounds have been long attributed to their ability to regulate antioxidant mechanisms in cells either by neutralizing reactive species and/or by regulating the expression levels of genes encoding for antioxidant enzymes [111]. One of such genes is *GPX1* that encodes for the most abundant and ubiquitously expressed member of the glutathione peroxidase family and catalyzes the reduction of hydrogen peroxide (H_2_O_2_) by glutathione protecting the cells against oxidative damage. *GPX1* expression is altered in several diseases and its overexpression confers resistance to oxidative damage but it has also been associated with certain disorders such as high glucose levels [112]. Regulating and maintaining adequate levels of GPX1 activity may be another important strategy to prevent or reduce the risk of these disorders. We found four human studies reporting the upregulation of the expression of *GPX1* in blood [18,39,40] and in neutrophils [45] in response to intervention with different sources of bioactive compounds, i.e., polyphenols from red wine [18] and mixed compounds in hazelnuts [40], Brazil nuts [39] and fermented papaya [45]. As in previous studies, there was no further evidence of the association with specific compounds or metabolites or of the translation into higher levels of the protein or of its activity. The levels of GPX1 mRNA, protein, and activity in tissues is known to be specifically responsive to dietary quantities of Se [112]. Whether the observed effects may be attributed to any of the bioactive compounds abundant in the Brazil nuts or to the presence of Se as suggested by Donadio et al., [39] is not known. More research is needed to further understand the role of GPX1 in oxidative, metabolic and inflammatory disorders and on the potential regulation of *GPX1* expression and activity by natural bioactive compounds.

Olive oil and broccoli are two of the most investigated food sources of the bioactive compounds, Tyr and HTyr [113] and SFGluc [114], respectively, with attributed beneficial effects against metabolic, inflammatory and oxidative disorders (Table 4). At the level of gene expression regulation in humans, olive oil with a high content of phenolic compounds has been reported to significantly downregulate the transcription factor *EGR1* [26,64] in mononuclear cells. EGR1 has an important role in the inflammatory response, it is involved in the proliferation, survival and differentiation of lymphocytes B and it has also been reported as an important hub protein deregulated in inflammatory and cardiovascular disorders as well as responsive to multiple stimuli [115]. On the other hand, broccoli containing SFGluc was found to upregulate the oxygenase *HMOX1* in blood [26], granulocytes [23] and cells from nasal lavage [22]. These results suggest that *EGR1* and *HMOX1* may also be part of the pool of human gene targets with the potentiality of responding to the intake of bioactive compounds with attributed anti-inflammatory and cytoprotective effects such as those present in the olive oil and the broccoli. In support of this, HTyr has been shown to induce the expression of *EGR1* in human promyelocytic leukemia cultured cells [116] and, the oral administration of sulforaphane or SFGluc promoted the appearance of derived metabolites and the induction of *HMOX1* in the breast tissue of rats and women [117]. Yet again, none of the previous human trials investigated the association of the regulation of *EGR1* or *HMOX1* with the presence of Tyr or HTyr or of sulphoraphane metabolites in the immune cells, blood samples or nasal cells. 

Overall, there is still limited evidence for the regulation of specific gene targets in human samples in response to the intake of bioactive compounds as well as poor agreement with results from previous pre-clinical studies. Some of the specific critical issues that may be implicated in this lack of accordance have been previously discussed [118]. Nevertheless, a number of genes (*PPARG*, *TNF*, *GPX1*, *EGR1*, *HMOX1*) are slowly emerging as potential molecular targets commonly responsive to different bioactive compounds that trigger a combination of interlinked metabolic, inflammatory, and antioxidant mechanisms that may underlay the health benefits of these compounds. Further studies are needed to confirm these and other molecular targets of the bioactive compounds. In addition to overcoming the technical inaccuracies and heterogeneity of the trials carried out so far, the interindividual variability in the bioavailability of the bioactive compounds and in the responses to these compounds is an essential feature inherent to humans that also needs to be addressed in future studies [100]. Different factors, i.e., sex, age, health status or genetic variation can greatly affect gene expression responses and must be taken into account in the design of future studies so that we can truly discern between the specific subpopulations of responders (gene up- or down-regulated) and non-responders (no change in the expression levels). Along these lines, a few studies included in this review have explored and reported the effects of specific genetic variation in the gene response to the intake of the test bioactive compounds [39,43,45,87]. It was noted that the expression of *GPX1* was upregulated upon consumption of Brazil nuts only in subjects with a CC genotype at the rs1050450 genetic variant of this gene [39]. Also, the single nucleotide polymorphisms of *NFE2L2* at the positions −617, −651 or −653 appear to be associated with increased expression of *NFE2L2* following coffee intake [43]. Other studies, however, did not find a clear genetic effect and thus, the Ser/Ser genotypes of *GSTM-1* or *hOGG1* did not influence the upregulation of *SOD1*, *CAT*, *GPX1*, *OGG1* following intervention with fermented papaya [45], or the presence of long or short GT genotypes in the promoter region of *HMOX1* did not affect the response to the consumption of Cur [87]. Although these results are very preliminary, they reflect the importance of including the study of the influence of genetic variation, as well as of other factors, to contribute to the understanding of the gene expression responses to dietary bioactive compounds. 

## 7. Concluding Remarks

This compilation of 75 human studies looking at gene expression effects that might be attributed to dietary bioactive compounds has shown that, most of these trials presented a range of critical limitations with regards to the study design, experimental settings, data analysis as well as poor reporting of essential features providing insufficient evidence of the specific reported gene changes. Although we are aware of the difficulties and economic constraints of implementing all the issues reviewed here and summarized in Table 5, we need to follow as many of these recommendations as possible. The aim is to increase the quality of future gene expression studies and the reliability of the genes selected and investigated as potential targets responsive to specific supplementation with bioactive compounds. The inclusion of individual gene expression results is relevant to get a more comprehensive picture of gene expression regulation in response to bioactive compounds without the need of doing expensive large trials. Obtaining more reliable gene targets in humans will also allow for better in vitro studies still essential to investigate and understand the cellular and mechanisms of action of these compounds.

## Figures and Tables

**Table 1:** List of reference genes reported in the human intervention studies included in this review.

Gene Symbol	*n* Studies ^1^	Cell/Tissue Samples in Which the Gene Has Been Used as a Reference Gene
Most common genes used as reference genes		
*GAPDH*	31 [4,16,18,20,25,26,28,32,33,34,35,37,39,40,44,45,46,49,50,51,53,55,57,64,65,67,72,73,78,79,86] 1 tested, but not used [63]	Blood, white blood cells, mononuclear cells, lymphocytes, neutrophils, gastric antrum, colon cancer and colon normal tissue, prostate hyperplasia and prostate cancer tissue, skeletal muscle, adipose tissues
*ACTB*	16 [14,18,40,41,42,43,46,48,58,63,64,70,76,81,82,84] 1 tested, but not used [53]	Blood, white blood cells, leukemic blasts, lymphocytes, CD14+ monocytes, colon cancer and colon normal tissue, skeletal muscle tissue, skin tissue
*18S rRNA*	11 [15,17,21,22,24,56,59,61,80,85,87] 1 considered, but not used [66]	White blood cells, mononuclear cells, skeletal muscle tissue, buccal swabs, gastric mucosa, adipose tissue, nasal cells, epidermis blister, buttock skin
Other genes less commonly used as reference genes		
*B2M*	6 [18,40,70,71,74,82]	Blood, mononuclear cells, leukemic blasts, colon tissue, skeletal muscle, adipose tissue
*HPRT1*	3 [18,38,40], 2 tested, but not used [4,72] 1 considered, but not used [66]	Blood, lymphocytes
*GUSB*	2 [29,52], 1 tested, but not used [4]	Oral mucosa, prostate tissue
*RPLP0*	3 [40,60,82], 1 tested, but not used [63]	Blood, leukemic blasts, lymphocytes, neutrophils
*RPL13A*	1 [27]	Mononuclear cells
*RPL32*	1 [36]	Adipose tissue
*HMBS*	2 [45,47]	Neutrophils, colon mucosa
*ATP5O*	1 [66]	Duodenal biopsies
*DUSP1*	1 [54]	Oral biopsies
*ALAS1*	1 [83]	Prostate cancer and normal tissue
*YWHAZ*	1 [38]	Lymphocytes
*UBC*	1 [58]	Mononuclear cells
*PPIA*	1 [58]	Mononuclear cells
*G6PD*	2 [66,72]	Blood (tested but not used), duodenal tissue (selected but not used)
Other reference molecules used		
*AW109*	1 [62]	Mononuclear cells
*ssDNA*	1 [75]	Skeletal muscle

^1^ Number of studies that report to have used and/or tested the reference gene. Genes nomenclature from GeneCards [92] (in alphabetical order): *ACTB*, actin beta; *ALAS1*, 5′-aminolevulinate synthase 1; *ATP5O*, ATP synthase, H+ transporting, mitochondrial F1 complex, O subunit; *AW109*, competitor RNA; *B2M*, beta-2-microglobulin; *DUSP1*, dual specificity phosphatase 1; *GAPDH*, glyceraldehyde-3-phosphate dehydrogenase; *GUSB*, glucuronidase beta; *G6PD*, glucose-6-phosphate dehydrogenase; *HMBS*, hydroxymethylbilane synthase (alias: *PBGD*, porphobilinogen deaminase); *HPRT1*, hypoxanthine phosphoribosyltransferase 1; *PPIA*, peptidylprolyl isomerase A; *RPLP0*, ribosomal protein lateral stalk subunit P0 (*36B4 rRNA:* encodes for RPLP0*)*; *RPL13A*, ribosomal protein L13A; *RPL32*, ribosomal protein L32; *18S rRNA*, 18S ribosomal RNA; *ssDNA*, single stranded DNA; *UBC*, ubiquitin C; *YWHAZ*, tyrosine 3-monooxygenase/tryptophan 5-monooxygenase activation protein zeta.

**Table 2:** Overview of the significant gene expression changes reported in peripheral blood isolated immune cells in response to different interventions with various diets, foods, or derived products containing bioactive compounds (*p*-value < 0.05).

Reference	Cells ^1^	Groups Compared	Potential Bioactive Compounds (Specifically Indicated in the Article)	Upregulated genes	Downregulated Genes	Main Biological Message Reported in the Article Potentially Associated with the Gene(s) Response
*White blood cells*						
Daak AA et al., 2015 [65]	White blood cells	Omega-3 capsules vs. placebo capsules (high oleic oil blend)	EPA, DHA	-	*NFKB1*	Improvement of oxidative stress status and amelioration of inflammation
Farràs M et al., 2013 [31]	White blood cells	Olive oil high polyphenols vs. low polyphenols	Mixed olive oil compounds (polyphenols)	*ABCA1*, *SCARB1*, *PPARG*, *PPARA*, *PPARD*, *MED1*, *CD36*, *PTGS1*	-	Enhancement of cholesterol efflux from cells
Nieman DC et al., 2007 [80]	White blood cells	Quer vs. placebo	Quer	-	*CXCL8*, *IL10*	Modulation of post-exercise inflammatory status
*Mononuclear cells*						
Konstantinidou V et al., 2010 [28]	Mononuclear cells	Med diet + olive oil vs. control diet	Mixed compounds in the Med diet (potential specific contribution of olive oil polyphenols)	-	*ADRB2*, *ARHGAP15*, *IL7R*, *POLK*, *IFNG*	Regulation of atherosclerosis-related genes, improvement of oxidative stress and inflammatory status
Radler U et al., 2011 [70]	Mononuclear cells	Low-fat yoghurt containing grapeseed extract + fish oil + phospholipids +L- Carn + VitC + VitE (post vs. pre)	Mixed compounds (polyphenols, fatty acids, vitamins)	*PPARG*, *CPT1A*, *CPT1B*, *CRAT*, *SLC22A5*	-	Regulation of fatty acids metabolism
Jamilian M et al., 2018 [67]	Mononuclear cells	Fish oil vs. placebo	Mixed compounds (EPA + DHA)	*PPARG*	*LDLR*, *IL1B*, *TNF*	Improvement of inflammatory status and of insulin and lipid metabolism
Plat J & Mensik RP, 2001 [62]	Mononuclear cells	Oils with mixed stanols vs. control (margarine + rapeseed oil)	Mixed compounds (stanol esters: sitos, camp)	*LDLR*	-	Improvement of LDL-cholesterol metabolism
Shrestha S et al., 2007 [57]	Mononuclear cells	*Psyllum* + plant sterols vs. placebo	Mixed compounds (*Psyllum* fiber + plant sterols)	*LDLR*	-	Improvement of LDL-cholesterol metabolism
Perez-Herrera A et al., 2013 [32]	Mononuclear cells	Sunflower oil vs. other oils (virgin olive oil, olive antioxidants, mixed oils)	Mixed compounds in sunflower oil	*CYBB*, *NCF1*, *CYBA*, *NFE2L2*, *SOD1*, *CAT*, *GSR*, *GSTP1*, *TXN*	-	Induction of postprandial oxidative stress (potential reduction by oil phenolics)
Rangel-Zuñiga OA et al., 2014 [33]	Mononuclear cells	Heated sunflower oil (post vs. pre)	Mixed compounds in sunflower oil	*XBP1*, *HSPA5*, *CALR*	-	Induction of postprandial oxidative stress
Camargo A et al., 2010 [27]	Mononuclear cells	Olive oil high polyphenols vs. low polyphenols	Mixed olive oil compounds (polyphenols)	-	*EGR1*, *IL1B*	Lessening of deleterious inflammatory profile
Castañer O et al., 2012 [30]	Mononuclear cells	Olive oil high polyphenols (Post vs. Pre) or Olive oil high polyphenols vs. low polyphenols	Mixed olive oil compounds (polyphenols)	-	*CD40LG*, *IL23A*, *IL7R*, *CXCR2*, *OLR1*, *ADRB2*, *CCL2*	Reduction of atherogenic and inflammatory processes
Martín-Peláez S et al ., 2015 [35]	Mononuclear cells	Olive oil high polyphenols (post vs. pre) or Olive oil high polyphenols vs. low polyphenols	Mixed olive oil compounds (polyphenols)	-	*ACE*, *NR1H2*, *ILR8*	Modulation of the renin-angiotensin-aldosterone system and systolic blood pressure
Boss A et al., 2016 [64]	Mononuclear cells	Olive leaf extract vs. placebo (glycerol +sucrose, no polyphenols)	Mixed olive leaf compounds (oleuropein, HTyr)	*ID3*	*EGR1*, *PTGS2*	Regulation of inflammatory and lipid metabolism pathways
Ghanim H et al., 2010 [58]	Mononuclear cells	*Polygonum cuspidatum* extract vs. placebo	Mixed compounds in *Polygonum cuspidatum* extract (Res)	*IRS1*	*MAPK8*, *KBKB*, *PTPN1*, *SOCS3*	Suppressive effect on oxidative and inflammatory stress
Barona J et al., 2012 [50]	Mononuclear cells	Grape powder vs. control	Mixed compounds in grape powder (polyphenols)	*NOS2*	-	Anti-oxidative and anti-inflammatory response
Tomé-Carneiro J et al., 2013 [51]	Mononuclear cells	Grape extract, Grape extract + Res (post vs. pre or extracts vs. placebo)	Mixed compounds in grape extract (Res)	*LRRFIP1*	*IL1β*, *TNF*, *CCL3*, *NFKBIA*	Beneficial immune-modulatory effect
Kropat C et al., 2013 [53]	Mononuclear cells	Bilberry pomace extract (post vs. pre)	Mixed compounds in bilberry pomace extract (anthocyanins)	*NQO1*	*HMOX1*, *NFE2L2*	Regulation of antioxidant transcription and antioxidant genes
*Isolated single type of cells*						
Persson I et al., 2000 [14]	Lymphocytes	Mix Veg (post vs. pre)	Mixed compounds in the Mix Veg	-	*GSTP1*	Compensatory downregulation of endogenous antioxidant systems
Hernández-Alonso P et al., 2014 [38]	Lymphocytes	Diet + pistacchio vs. control diet	Mixed compounds in the pistachio (fatty acids, minerals, vitamins, carotenoids, tocopherols polyphenols)	-	*IL6*, *RETN*, *SLC2A4*	Impact on inflammatory markers of glucose and insulin metabolism
Boettler U et al., 2012 [43]	Lymphocytes	Coffee brew (post vs. pre)	Mixed compounds in coffee (CGA, NMP)	*NFE2L2*	-	Regulation of antioxidant transcription
Volz N et al., 2012 [42]	Lymphocytes	Coffee brew (post vs. pre)	Mixed compounds in coffee (CGA, NMP)	*NFE2L2*	*HMOX1*, *SOD1*	Regulation of antioxidant transcription and antioxidant genes
Morrow DMP et al., 2001 [79]	Lymphocytes	Quer vs. placebo	Quer	-	*TIMP1*	Mediator of carcinogenic processes
Marotta F et al., 2010 [45]	Neutrophils	Fermented papaya (Post vs. Pre)	Mixed compounds in fermented papaya	*SOD1*, *CAT*, *GPX1*, *OGG1*	-	Regulation of redox balance
Carrera-Quintanar L et al., 2015 [60]	Neutrophils	T_1_: *Lippia citriodora* extract T_2_: Almond beverage (+vitC + vitE) T_1_ + T_2_ (post vs. pre)	Mixed compounds	-	*SOD2*, *SOD1*	Adaptative antioxidant response
Yanaka A et al., 2009 [23]	Polymorpho-nuclear granulocytes	Broccoli sprouts (post vs. pre)	Mixed compounds (SFGluc)	*HMOX1*	-	Protective effect against bacterial infection (anti-oxidative, anti-inflammatory)

^1^ White blood cells or Leukocytes: mononuclear cells agranulocytes (lymphocytes and monocytes) and polymorphonuclear granulocytes (neutrophils, eosinophils, basophils, mast cells); Lymphocytes (T cells, B cells, NK cells). Table abbreviations (in alphabetical order): camp, campestanol; CGA, chlorogenic acid; DHA, docosahexaenoic acid; EPA, eicosapentaenoic acid; HTyr, hydroxytyrosol; l-Carn, l-carnitine; LDL, low-density lipoprotein; Med, Mediterranean; NMP, *N*-methylpyridinum; post-, after treatment; pre-, baseline or before treatment; Quer, quercetin; Res, resveratrol; SFGluc, sulforaphane glucosinolates; sitos, sitostanol; Veg, vegetables; VitC, vitamin C; VitE, vitamin E. Genes nomenclature from GeneCards [92] (in alphabetical order) : *ABCA1*, ATP binding cassette subfamily A member 1; *ACE*, angiotensin I converting enzyme; *ADRB2*, adrenoceptor beta 2; *ARHGAP15*, rho GTPase activating protein 15; *CALR*, calreticulin (alias: *CRT*); *CAT*, catalase; *CCL2*, C-C motif chemokine ligand 2 (alias: *MCP-1*, monocyte chemotactic protein 1); *CCL3*, C-C motif chemokine ligand 3; *CD36*, CD36 molecule; *CD40LG*, CD40 ligand; *CPT1A*, carnitine palmitoyltransferase 1A; *CRAT*, carnitine O-acetyltransferase; *CXCL8*, C-X-C motif chemokine ligand 8 (alias: *IL8*); *CXCR2*, C-X-C motif chemokine receptor 2 (alias: *IL8RA*); *CYBA*, cytochrome B-245 alpha chain (alias: *P22-Phox*); *CYBB*, cytochrome B-245 beta chain (alias: *NOX2*, *GP91-Phox*)*; EGR1*, early growth response 1; *GPX1*, glutathione peroxidase 1; *GSTP1*, glutathione S-transferase Pi 1; *GSR*, glutathione-disulfide reductase (alias: *GRD1*); *HMOX1*, heme oxygenase 1 (alias:*HO-1*); *HSPA5*, heat shock protein family A (Hsp70) member 5 (alias: *BIP*); *ID3*, inhibitor of DNA binding 3, HLH protein; *IFNG*, interferon gamma; *IL1B*, interleukin 1 beta; *IL6*, interleukin 6; *IL7R*, interleukin 7 receptor; *IL10*, interleukin 10; *IL23A*, interleukin 23 subunit alpha; *IRS1*, insulin receptor substrate 1; *LDLR*, low density lipoprotein receptor; *LRRFIP1*, LRR binding FLII interacting protein 1; *MAPK8*, mitogen-activated protein kinase 8 (alias: *JKN1*); *MED1*, mediator complex subunit 1 (alias: *PPARBP*); *NFE2L2*, nuclear factor, erythroid 2 like 2 (alias: *NRF2*); *NQO1*, NAD(P)H quinone dehydrogenase 1; *NOS2*, nitric oxide synthase 2 (alias: *iNOS*); *NR1H2*, nuclear receptor subfamily 1 group H member 2; *OGG1*, 8-oxoguanine DNA glycosylase; *OLR1*, oxidized low density lipoprotein receptor 1; *POLK*, DNA polymerase kappa; *PPARA*, peroxisome proliferator activated receptor alpha; *PPARD*, peroxisome proliferator activated receptor delta; *PPARG*, peroxisome proliferator activated receptor gamma; *PTGS1*, prostaglandin-endoperoxide synthase 1 (alias: *COX1*); *PTGS2*, prostaglandin-endoperoxide synthase 2 (alias: *COX2*); *PTPN1*, protein tyrosine phosphatase, non-receptor type 1 (alias: *PTP1B*); *RETN*, resistin; *SCARB1*, scavenger receptor class B member 1 (alias: *SRB1*); *SLC2A4*, solute carrier family 2 member 4 (alias: *GLUT4*); *SLC22A5*, solute carrier family 22 member 5 (alias: *OCTN2*); *SOCS3*, suppressor of cytokine signaling 3; *SOD1*, superoxide dismutase 1 (alias: *Cu/ZnSOD*); *SOD2*, superoxide dismutase 2 (alias: *MnSOD*); *TIMP1*, TIMP metallopeptidase inhibitor 1; *TNF*, tumor necrosis factor (alias: *TNFα*); *TXN*, thioredoxin; *XBP1*, X-box binding protein 1. Orange color: upregulated genes; green color: downregulated genes.

**Table 3:** Overview of the significant expression changes reported for specific gene targets in different cells and tissue samples following intervention with diet, foods, or derived products containing bioactive compounds. Analysis of the evidence supporting the changes and, the potentiality of these changes as a mechanism of action underlying the beneficial effects attributed to these products and bioactive compounds (*p*- value < 0.05).

Reference	Cells ^1^ (*n* = Number of Samples Analysed)	Groups Compared	Potential Bioactive Compounds (Specifically Indicated in the Article)	Gene Expression Change (FC; % of Change)	Data Quality	Variability (Estimated CV %)	Association with Specific Compounds, Metabolites	Protein Change	Level of Evidence
*Inflammation:* TNF									
Di Renzo L. et al., 2017 [40]	Blood (*n* = 22)	McD meal + hazelnuts vs. McD meal (post-)	Mix compounds present in the hazelnuts (fatty acids, polyphenols, etc.)	↓*TNF* (FC < -1.5; −34%)	Poor	No information available	No evidence	No evidence	Low
Weseler AR et al., 2011 [49]	Blood (*n* = 15)	Grape seeds (post- vs. pre-)	Mix compounds present in the seeds (flavanols)	↓*TNF* (FC = -1.14; −12%)	Poor	No information available	No evidence	Inhibition of ex vivo LPS-induced TNF in blood	Low
Tomé-Carneiro J et al., 2013 [51]	Mononuclear cells (*n* = 9–13)	Grape extract + Res (post- vs. pre-) Grape extract + Res vs. control (post-)	Mix compounds present in the grape extract + Res	↓*TNF* (FC = −2.56 to −1.54; −61% to −35%)	High	3.2–7.9 (only baseline levels)	No evidence	(NC) TNF in serum or plasma	Low
Jamilian M et al., 2018 [67]	Mononuclear cells (*n* = 20)	Fish oil (EPA + DHA) vs. placebo (post-)	Fish oil compounds (EPA + DHA)	↓*TNF* (FC = −1.12; −11%)	Medium-Poor	10.5–18.0	No evidence	No evidence	Low
Vors C. et al., 2017 [72]	Blood (*n* = 44)	EPA vs. control (post-) DHA vs. control (post-)	EPA, DHA	↑*TNF* (EPA, FC = +1.07; 7%)(DHA, FC = +1.09; 9%)	Poor	19.8–24.2	No evidence	No correlation between TNF and TNF in plasma	Low
*Energy metabolism: PPARs*									
Radler U et al., 2011 [70]	Mononuclear cells (*n* = 20–22)	Low-fat yoghurt (grapeseed extract + fish oil + phospholipids + l-carn + vitC + vitE) (post- vs. pre-)	Mix compounds (PUFAs, polyphenols, l-carn)	↑*PPARG* (FC = +2.53; 153%)	Poor	49.8	No evidence	No evidence	Low
Vors C et al., 2017 [72]	Blood (*n* = 44)	EPA vs. control (post-) DHA vs. control (post-)	EPA DHA	↑*PPARA* (EPA, FC = +1.12; 12%) (DHA, FC = +1.10; 10%)	Poor	17.9–26.1	No evidence	No evidence	Low
Jamilian M et al., 2018 [67]	Mononuclear cells (*n* = 20)	Fish oil (EPA + DHA) vs. placebo (post-)	Fish oil compounds (EPA + DHA)	↑*PPARG* (FC = +1.06; 6%)	Medium-Poor	9.0–11.3	No evidence	No evidence	Low
Farràs M al., 2013 [31]	White blood cells (*n* = 13)	High- vs. low-polyphenols in olive oil	Mix olive oil compounds (polyphenols)	↑*PPARG* (FC = +2.8; 180%) ↑*PPARA* (FC = +2.0; 100%) ↑*PPARD* (FC = +2.0; 100%) ↑*MED1* (FC = +1.45; 45%)	Poor	32.2–118.3	No evidence	No evidence	Low
*Antioxidant system: GPXs*									
Di Renzo L et al., 2014 [18]	Blood (*n* = 24)	Red wine Med meal + red wine McD meal + red wine (post- vs. pre-)	Mix compounds in red wine	↑*GPX1* (FC = +1.41; 41%) (FC = +1.52; 52%) (FC = +2.83; 183%)	Poor	No information available	No evidence	No evidence	Low
Di Renzo L. et al., 2017 [40]	Blood (*n* = 22)	McD meal + hazelnuts vs. McD meal (post-)	Mix compounds present in the hazelnuts (fatty acids, polyphenols, etc.)	↑*GPX1*, *GPX3*, *GPX4* (F > +1.5; 50%)	Poor	No information available	No evidence	No evidence	Low
				↓*GPX7* (FC < −1.5; −33%)					
Donadio JLS et al., 2017 [39]	Blood (unclear, *n* = 130 or *n* = 12?)	Brazil nuts (with Se) (post- vs. pre-)	Mix compounds present in the brazil nuts (Se, fatty acids, polyphenols, etc.)	↑*GPX1* (FC = +1.3; 30% for a particular genotype)	Poor	No information available	No evidence	No evidence	Low
Marotta F et al., 2010 [45]	Neutrophils (*n* = 11)	Fermented papaya (post- vs. pre-)	Mix compounds present in the fermented papaya	↑*GPX1* (FC = +61–67; >6000% (?))	Poor	No information available	No evidence	No evidence	Low

^1^ White blood cells or Leukocytes: mononuclear cells agranulocytes (lymphocytes and monocytes) and polymorphonuclear granulocytes (neutrophils, eosinophils, basophils, mast cells). Table abbreviations (in alphabetical order): CV, coefficient of variation; DHA, docosahexaenoic acid; EPA, eicosapentaenoic acid; FC, fold-change; l-carn, l-carnitine; LPS, lipopolysaccharide; McD, MacDonald; Med, Mediterranean; NC, no change; post-, after treatment; pre-, baseline or before treatment; PUFA, polyunsaturated fatty acids; Res, resveratrol; Se, selenium; VitC, vitamin C; VitE, vitamin E. Genes nomenclature from GeneCards [92] (in alphabetical order): *GPX1-4*, glutathione peroxidase 1-4; *MED1*, mediator complex subunit 1 (alias: *PPARBP*); *PPARA*, peroxisome proliferator activated receptor alpha; *PPARD*, peroxisome proliferator activated receptor delta; *PPARG*, peroxisome proliferator activated receptor gamma; *TNF*, tumour necrosis factor (alias: *TNFα*). Orange color: upregulated genes; green color: downregulated genes.

**Table 4:** Overview of the significant gene expression changes attributed to the intervention with specific foods or derived products containing bioactive compounds and reported in different cells and tissue samples (*p*-value < 0.05).

Reference	Cells ^1^	Groups Compared	Potential Bioactive Compounds (Specifically Indicated in the Article)	Upregulated Genes	Downregulated Genes	Genes not Changing or with a Not Significant Change	Association with Metabolites	Effect on Protein Levels	Main biological Message Reported
*Olive oil and derived products*									
Farràs M et al., 2013 [31]	White blood cells ^1^	High vs. moderate polyphenol olive oil (post-)	Olive oil polyphenols	*ABCA1*, *SCARB1*, *PPARG*, *PPARA*, *PPARD*, *MED1*, *CD36*, *PTGS1*	-	(NC) *ABCG1*, *PTGS2*	↑HTyr acetate in plasma with ↑*ABCA1*	NR	Enhancement of cholesterol efflux from cells
Konstantinidou V et al., 2010 [28]	Mononuclear cells	Med diet + olive oil (polyphenols) vs control diet (post-)	Mix compounds present in the Med diet and the olive oil (fatty acids, polyphenols, vitamins etc.)	-	*ADRB2*, *ARHGAP15*, *IL7R*, *POLK*, *IFNG*	-	↓*IFNG* with ↑Tyr in urine (highest dose of olive oil)	↓ IFNγ in plasma (post- vs. pre-)	Regulation of atherosclerosis-related genes, improvement of oxidative stress and inflammatory status
Camargo A et al., 2010 [27]	Mononuclear cells	High vs. low polyphenol olive oil (post-)	Olive oil polyphenols	-	*EGR1*, *IL1B*	(NS↓) *JUN*, *PTGS2*	NR	NR	Lessening of deleterious inflammatory profile
Castañer O et al., 2012 [30]	Mononuclear cells	High vs. low polyphenol olive oil (post-)	Olive oil polyphenols	-	*CD40LG*, *IL23A*, *IL7R*, *CXCR2*, *OLR1*, *ADRB2*, *CCL2*	(NS↓) *IFNG*, *VEGFB*, *ICAM1* (NC) *ALOX5AP*, *TNFSF10*	↓*OLR1* with the ↑Tyr and ↑HTyr in urine	↓CCL2	Reduction of atherogenic and inflammatory processes
Hernáez Á et al., 2015 [34]	Mononuclear cells	High vs. low polyphenol olive oil (post-)	Olive oil polyphenols	-	-	(NS↑) *LPL*	NR	NR	Reduction of LDL concentrations and of LDL atherogenicity
Martín-Peláez S et al., 2015 [35]	Mononuclear cells	High vs. low polyphenol olive oil (post-)	Olive oil polyphenols	-	*ACE*, *NR1H2*, *CXCR2*	(NS↓) *CXCR1*, *ADRB2*, *MPO*, *ACE* (NC) *ECE2*, *OLR1*	NR	NR	Modulation of the renin-angiotensin-aldosterone system and systolic blood pressure
Crespo MC et al., 2015 [63]	Mononuclear cells	Olive mill waste water extract Hytolive (enriched in HTyr) vs. placebo (post-)	Olive waste polyphenols (HTyr)	-	-	(NC) Phase II enzymes*: NQO1*,*2*, *GSTA1*,*4*, *GSTK1*, *GSTM1-5*, *GSTO1*,*2*, *GSTP1*, *GSTM1*,*2*, *HNMT*, *INMT*, *MGST1-3*	NR	NR	Hormesis hypothesis of activation of phase II enzymes by polyphenols
Boss A et al., 2016 [64]	Mononuclear cells	Olive leaf extract (oleuropein, HTyr) vs. placebo (post-)	Olive leaf polyphenols (oleuropein, HTyr)	*ID3*	*EGR1*, *PTGS2*	-	NR	NR	Regulation of inflammatory and lipid metabolism pathways
Kruse M et al., 2015 [36]	Adipose tissue	Olive oil (MUFA) (post- vs. pre-, postpandrial)	Mix olive oil bioactive compounds	*CCL2*	-	(NS↓) *IL6*, *IL8* (NS↑) *IL10*, *TNF* (NC) *IL1β*, *ADGRE1*, *SERPINE1*	NR	(NC) MCP-1 (CCL2)	Acute inflammatory and metabolic response related genes
*Broccoli and derived products*									
Atwell LL et al., 2015 [25]	Blood	Broccoli sprout (SFGluc) vs. Myrosinase-treated broccoli sprout extract (SFGluc) (post- vs. pre-)	SFGluc	-	-	(NC) *CDKN1A*, *HMOX1*	NR	(NC) plasma levels of HMOX1 (HO-1)	Search for chemopreventive targets
Doss JF et al., 2016 [26]	Blood	Broccoli (SFGluc)	SFGluc	*HBG1*, *HMOX1*	-	(NS↑) *NQO1*	NR	(NC) Hbg1 or HbF	Gene expression studies in sickle cell disease (oxidative stress related)
Riso P et al., 2010 [24]	Mononuclear cells	Broccoli (SFGluc, Lut, *β*-car, VitC) (post- vs. pre-)	SFGluc, Lut, *β*-car, VitC	-	-	(NC) *OGG1*, *NUDT1*, *HMOX1*	NR	NR	Antioxidant protection related to DNA repairing enzymes
Yanaka A et al., 2009 [23]	Polymorpho-nuclear granulocytes	Broccoli sprout (SFGluc) (post- vs. pre-)	SFGluc	*HMOX1*	-	-	NR	NR	Protective effect against bacterial infection (anti-oxidative, anti-inflammatory)
Gasper AV et al., 2007 [21]	Gastric antrum	Broccoli drink (containing SFGluc) (post- vs. pre-)	SFGluc	*GCLM*, *TXNRD1*	-	(NC) *CDKN1A*	NR	NR	Effect on xenobiotic metabolism
Riedl MA et al., 2009 [22]	Cells from nasal lavage	Broccoli sprout (SFGluc) (different doses) vs. control (alfalfa sprout)	SFGluc	*GSTM1*, *GSTP1*, *NQO1*, *HMOX1*	-	-	NR	NR	Effect on Phase II metabolism
*Grape products and compounds (Res)*									
Weseler AR et al., 2011 [49]	Blood	Flavanols isolated from grape seeds (post- vs. pre-)	Flavanols	-	*IL6*, *TNF*, *IL10*	(NS↓) *CAT*, *GSR*, *HMOX1* (NC) *IL1β*, *CXCL8*, *NOS2*, *NFKBIA*, *ICAM1*, *VCAM1*, *GPX1*, *GPX4*, *SOD2*	NR	Plasma: ↓TNF (NC) IL10	Anti-inflammatory effects in blood
Barona J et al., 2012 [50]	Mononuclear cells	Grape powder vs. placebo	Mix compounds in grape (flavonoids)	*NOS2* (individuals without dyslipidemia)	-	(NC) *CYBB*, *SOD1*, *SOD2*, *GPX1*, *GPX4*	NR	NR	Anti-oxidative and anti-inflammatory response
Tomé-Carneiro J et al., 2013 [51]	Mononuclear cells	Grape extract vs Grape extract + Res (post- vs. pre-)	Polyphenols, Res	*LRRFIP1*	*IL1β*, *TNF*, *CCL3*, *NFKBIA*	(NC) *NFKB1*	NR	NC in TNF levels in PBMC or serum	Beneficial immune-modulatory effect
Nguyen AV et al., 2009 [48]	Colon tissue (cancer and normal)	Low concentration of grape powder (post- vs. pre-)	Res, flavanols, flavans, anthocyanins, catechin	Normal tissue *MYC* Cancer tissue *MYC*, *CCND1*	Normal tissue *CCND1*, *AXIN2*	-	NR	NR	Effect on cancer related pathway
Mansur AP et al., 2017 [78]	White blood cells	Res (post- vs. pre-; T vs. C)	Res	-	-	(NC) *SIRT1*	NR	↑ Serum hSIRT1	Comparative study with caloric restriction
Chachay VS et al., 2014 [76]	Mononuclear cells	Res (from *Polygonium cuspidatum*)	Res	-	-	(NC) *NQO1*, *PTP1B*, *IL6*, *HMOX1*	NR	↓plasma IL6	Effects on non-alcoholic fatty liver disease
Yiu EM et al., 2015 [77]	Mononuclear cells	Res (two doses) (post- vs. pre-)	Res	-	-	(NC) *FXN* (dose 1) (NS↓) *FXN* (dose 2)	NR	(NC) FXN in PBMC	Effect on the neurodegenerative disease (Friedreich ataxia)
Olesen J et al., 2014 [75]	Skeletal muscle	Res (post- vs. pre-; T vs. C)	Res	-	-	(NC) *PPARGC1A*, TNF, *NOS2*	NR	(NC) TNF, iNOS in muscle and plasma	Metabolic and inflammatory status
Yoshino J et al., 2012 [73]	Skeletal muscle and adipose tissue	Res (post- vs. pre-; T vs. C)	Res	-	-	(NC) *SIRT1*, *NAMPT*, *PPARGC1A*, *UCP3*	NR	NR	Metabolic effects
Poulsen MM et al., 2013 [74]	Skeletal muscle and adipose tissue	Res (post- vs. pre-)	Res	-	Muscle *SLC2A4*	Muscle (NC) *PPARGC1A* Adipose (NC*) TNF*, *NFKB1*	NR	NR	Metabolic and inflammatory effects

^1^ White blood cells or Leukocytes: mononuclear cells agranulocytes (lymphocytes and monocytes) and polymorphonuclear granulocytes (neutrophils, eosinophils, basophils, mast cells). Table abbreviations (in alphabetical order): β-car, β-carotene; HTyr, hydroxytyrosol; LDL, low-density lipoprotein; Lut, lutein; Med, Mediterranean; NC, no change; NR, not reported; NS, not significant; MUFA, monounsaturated fatty acids; PBMC, peripheral blood mononuclear cells; post-, after treatment; pre-, baseline or before treatment; Res, resveratrol; SFGluc, sulphoraphane glucosinolates; Tyr, tyrosol; VitC, vitamin C. Genes nomenclature from GeneCards [92] (in alphabetical order): *ABCA1*, ATP binding cassette subfamily A member 1; *ABCG1*, ATP binding cassette subfamily G member 1; *ACE*, angiotensin I converting enzyme; *ADRB2*, adrenoceptor beta 2; *ADGRE1*, adhesion G protein-coupled receptor E1 (alias: *EMR1*); *ALOX5AP*, arachidonate 5-lipoxygenase activating protein; *ARHGAP15*, rho GTPase activating protein 15; *CAT*, catalase; *CCL2*, C-C motif chemokine ligand 2 (alias: *MCP-1*, monocyte chemotactic protein 1); *CCL3*, C-C motif chemokine ligand 3; *CCND1*, cyclin D1; *CD36*, CD36 molecule; *CD40LG*, CD40 ligand; *CDKN1A*, cyclin dependent kinase inhibitor 1A (alias: *p21*); *CXCL8*, C-X-C motif chemokine ligand 8 (alias: *IL8*); *CXCR1*, C-X-C motif chemokine receptor 1; *CXCR2*, C-X-C motif chemokine receptor 2 (alias: *IL8RA*); *CYBB*, cytochrome B-245 beta chain (alias: *NOX2*, *GP91-Phox*)*; ECE2*, endothelin converting enzyme 2; *EGR1*, early growth response 1; *EMR1*, *FXN*, frataxin, Friedreich ataxia protein; *GCLC*, glutamate-cysteine ligase catalytic subunit (alias: *γGCL*); *GPX1*, glutathione peroxidase 1; *GPX4*, glutathione peroxidase 4; *GSTA1*, glutathione *S*-transferase alpha 1; *GSTA4*, glutathione *S*-transferase alpha 4; *GSTK1*, glutathione *S*-transferase kappa 1; *GSTM1*, glutathione *S*-transferase Mu 1; *GSTM2*, glutathione *S*-transferase Mu 2; *GSTM3*, glutathione *S*-transferase Mu 3; *GSTM4*, glutathione *S*-transferase Mu 4; *GSTM5*, glutathione *S*-transferase Mu 5; *GSTO1*, glutathione *S*-transferase omega 1; *GSTO2*, glutathione *S*-transferase omega 2; *GSTP1*, glutathione *S*-transferase Pi 1; *GSR*, glutathione-disulfide reductase (alias: *GRD1*); *HBG1*, hemoglobin subunit gamma 1; *HMOX1*, heme oxygenase 1 (alias:*HO-1*); *HNMT*, histamine *N*-methyltransferase; *ICAM1*, intercellular adhesion molecule 1; *ID3*, inhibitor of DNA binding 3, HLH protein; *IFNG*, interferon gamma; *IL1B*, interleukin 1 beta; *IL6*, interleukin 6; *IL7R*, interleukin 7 receptor; *IL8*, interleukin 8*; IL10*, interleukin 10; *IL23A*, interleukin 23 subunit alpha; *INMT*, indolethylamine *N*-methyltransferase; *JUN*, jun proto-oncogene, AP-1 transcription factor subunit; *LPL*, lipoprotein lipase; *LRRFIP1*, LRR binding FLII interacting protein 1; *MED1*, mediator complex subunit 1 (alias: *PPARBP*); *MGST1*,microsomal glutathione *S*-transferase 1; *MGST2*, microsomal glutathione *S*-transferase 2; *MGST3*, microsomal glutathione *S*-transferase 3; *MPO*, myeloperoxidase; *MYC*, MYC proto-oncogene, BHLH transcription factor; *NAMPT*, nicotinamide phosphoribosyltransferase; *NFKB1*, nuclear factor kappa B subunit 1; *NFKBIA*, NFKB inhibitor alpha; *NQO1*, NAD(P)H quinone dehydrogenase 1; *NQO2*, *N*-ribosyldihydronicotinamide: quinone reductase 2; *NOS2*, nitric oxide synthase 2 (alias: *iNOS*); *NR1H2*, nuclear receptor subfamily 1 group H member 2; *NUDT1*, nudix hydrolase 1; *OGG1*, 8-oxoguanine DNA glycosylase; *OLR1*, oxidized low density lipoprotein receptor 1; *POLK*, DNA polymerase kappa; *PPARA*, peroxisome proliferator activated receptor alpha; *PPARD*, peroxisome proliferator activated receptor delta; *PPARG*, peroxisome proliferator activated receptor gamma; *PPARGC1A*, PPARG coactivator 1 alpha (alias: *PGC1α*); *PTGS1*, prostaglandin-endoperoxide synthase 1 (alias: *COX1*); *PTGS2*, prostaglandin-endoperoxide synthase 2 (alias: *COX2*); *PTPN1*, protein tyrosine phosphatase, non-receptor type 1 (alias: *PTP1B*); *SCARB1*, scavenger receptor class B member 1 (alias: *SRB1*); *SERPINE1*, Serpin Family E Member 1; *SIRT1*, sirtuin 1; *SLC2A4*, solute carrier family 2 member 4 (alias: *GLUT4*); *SOD1*, superoxide dismutase 1 (alias: *Cu/ZnSOD*); *SOD2*, superoxide dismutase 2 (alias: *MnSOD*); *TNF*, tumor necrosis factor (alias: *TNFα*); *TNFSF10*, TNF Superfamily Member 10; *TXNRD1*, thioredoxin reductase 1 (alias: *TR1*); *UCP3*, uncoupling protein 3; *VCAM1*, vascular cell adhesion molecule 1; *VEGFB*, vascular endothelial growth factor B. Orange color: upregulated genes; green color: downregulated genes.

**Table 5:** General recommendations to further enhance the quality and relevance of future intervention trials looking at the effects on gene expression of dietary bioactive compounds in humans.

Specific critical issues related to the human gene expression studies revised in this article that need improvement	Strategies to improve the quality of the studies and the level of evidence to support the link between gene response-bioactive compound
**Human clinical trial design**	
○ **Study design** ✓ A considerable proportion of the studies analyzed in this review were designed as single arm studies with no appropriate control group included. ✓ Many of the randomized controlled studies were designed in a parallel fashion. ✓ Studies conducted with an appropriate placebo group were scarce and most studies used other comparisons: low vs. high doses of the bioactive compounds. In comparisons carried out between different diets or foods or food products (complex mix of compounds), it will be difficult to assign a gene response to a particular bioactive compound(s).	○ **Study design** ✓ Gene expression studies in human trials must include appropriate and well-described control or reference groups to which the intervention group can be compared to [89]. ✓ A crossover design may be preferred since these kind of studies reduce the between subject variability [89]. ✓ Whenever possible appropriate placebos containing all the compounds in the test product except for the specific bioactive compound(s) to be investigated should be applied and described [89]. Alternatively and (o) additionally, different doses (low vs. high) of the bioactive compound(s) may be used.
○ **Sample population description** ✓ Most of the studies conducted so far have included limited information of the volunteers taking part in the study that, in general, are described as a group (average values, ranges), e.g., mixed cohorts of men and women, different ranges of age, lifestyle, health status, etc. ✓ Regarding the health status, about 60% of the gene expression studies included in this review was carried out in healthy participants only.	○ **Sample population description** ✓ Future studies should specify as many variables of the test population as possible: sex, age, ethnic group, lifestyle habits, etc., and, if possible, at the individual level (e.g., by means of Supplementary Material). This will contribute to the understanding of gene expression variability and population distribution of gene expression responses and the factors that may affect it [100]. ✓ More studies in groups with specific diseases are needed. Of especial consideration should be the individual characterization of the patients investigated, in particular, with regards to specific molecular targets identified to be implicated in the disease development and/or in the pathology itself.
○ **Sample size** ✓ In general, the sample size of the control and intervention groups used in the trials gathered here was small and insufficient to clearly discern significant and reliable changes in gene expression in human tissue samples in response to the treatment and/or, to establish the actual distribution of gene expression changes in the test and control population.	○ **Sample size** ✓ An increase of the sample size is needed but, sample size for gene expression studies may vary depending on the gene, type of sample, and other factors. As we learn more about gene expression interindividual variability and about the effect sizes (gene expression changes) in response to dietary bioactives, we shall be able to establish more appropriate sample sizes for each study.
○ **Type of intervention** ✓ Mixed compounds or single compounds? ✓ Foods or extracts (pills)? ✓ Duration: continued or acute or postprandial? ✓ Doses of the compounds and products tested are very variable. Is there an effective dose? Are high doses better than low doses?	○ **Type of intervention** ✓ More studies using single highly pure compounds are needed in order to unambiguously prove the potential effects of those compounds or of their derived metabolites in gene expression in specific cells or tissues. ✓ Mixed compounds in the form of extracts or complex foods should also be investigated to understand potential interactions or synergisms occurring between compounds and/or the effect of matrix on gene expression effects. ✓ One important issue to clarify is the differences between immediate (rapid) gene expression responses (short term after intake of the compounds) and those long term responses (after prolonged consumption) and their relevance in a specific disease. An additional critical point is the continuity or reversibility of those responses (epigenetics vs. temporal gene expression regulation). ✓ Dose-response studies should be implemented to establish the most effective concentration of a compound to induce a particular gene expression change.
**qRT-PCR experimental protocols**	
○ **Samples processing and characterization prior to RNA extraction** ✓ This review has evidenced a general lack of information about cell composition and heterogeneity of the blood cell and tissue samples used in the different trials. ✓ There was also little or poorly described information about the procedures applied for sample extraction and processing as well as about storage conditions and time elapsed during sample preparation or storage until further use for RNA extraction.	○ **Samples processing and characterization prior to RNA extraction** ✓ Knowledge of the heterogeneity and cell composition of the human blood cells and tissue samples used in the study is necessary. Researchers should include as many details as possible about the sample characterization. ✓ Sample preparation protocols can have a large impact on gene expression [4]. It should be a generalized practice within this type of human studies to include as many details as possible about the protocols for sample extraction, processing and storage conditions including the time periods involved in each stage.
○ **RNA extraction protocols, yielding and purity** ✓ Most of the studies included in this review have used validated commercial RNA extraction kits for RNA isolation. Nevertheless, there is a general lack of detailed description of the protocols and no indication of important issues such as the inclusion of DNAse treatment to remove genomic DNA. Although most studies indicate the measurement of RNA quantity and quality, most reports do not include data about the RNA yield or the quality results (e.g., RIN value)	○ **RNA extraction protocols, yielding and purity** ✓ Future studies looking at gene expression effects of bioactive compounds must include detailed information of the RNA extraction protocol with indication of genomic DNA removal, yield of RNA attained in the different tissue samples and specifically indicate the quality of the RNA samples used in the study by indicating the RIN value. It has been recommended that values at least above 5.0, that indicate a good total RNA quality [101], should be used.
○ **Reference genes selection and stability analysis** ✓ Most of the studies included in this review have used indistinctively *GAPDH*, *ACTB* or *18S rRNA* as reference genes for data normalization in a variety of human cell and tissue samples. There were very few studies applying a specific criteria or stability analysis to test and select the most appropriate reference gene.	○ **Reference genes selection and stability analysis** ✓ It is essential that future studies looking at gene expression effects of bioactive compounds include a validation assessment of the most suitable reference genes for normalization of gene expression results. This should be implemented for each tissue sample and experimental condition tested [91].
**Data comparison and presentation**	
○ **Comparative strategy.** ✓ Most gene expression changes have been reported using different comparative strategies. In many of the studies examined here we found that the expression changes were referred either only to the comparison between control and treatment groups at the end of the intervention, or comparing the post- and pre-intervention time points only for the treated group.	○ **Comparative strategy.** ✓ For highest evidence of the occurrence of a gene expression change as a consequence of a particular intervention with a source of bioactive compound(s) it is important to establish: ⇨ (1) baseline gene expression conditions in the control and treated groups (is there already any difference or not?), ⇨ (2) gene expression changes in the control volunteers, post-intervention vs. baseline (is the gene changing in the control group?), ⇨ (3) gene expression changes in the treated group, post-intervention vs. baseline (is the gene changing in the treated group? Is this change different to the control group?), ⇨ (4) post-intervention comparison between the control and treated groups (can we corroborate the differences between the two groups?). ✓ Additional evidence should be provided by dose/response studies, different time-points and by showing the reversal of the change and the return to the baseline conditions after a washout period.
○ **Results presentation: value of change.** ✓ We found a wide variety of results presentation to indicate the changes in gene expression, i.e., FC-value, ratio and log_2_ (ratio), % of change, mRNA expression levels, arbitrary units, number of mRNA copies. Very often, the results were presented only in figures from where the actual average and dispersion values were difficult to infer.	○ **Results presentation: value of change.** ✓ Although all the indicated ways of presenting gene expression changes used in the different studies are valid, we propose that standardization to FC-values and % of change attributed to the intervention with bioactives should be implemented so that future results from different studies can more easily be compared by means, e.g., of a meta-analysis. Also, and to improve the clarity of presentation, in addition to figures, appropriate tables including all these data are strongly recommended.
○ **Results presentation: data summary and distribution (variability).** ✓ Many studies failed to report whether the gene expression changes investigated followed a normal distribution and to clearly indicate any measure of the average and dispersion (variability) employed. ✓ Those studies that did report gene expression average changes indicated the final results mostly as the arithmetic mean value followed by either the SEM or the SD. The median value and IQR were applied only in a few studies. ✓ The significance of the changes was generally presented as a *p*-value and in most cases *p* < 0.05 was accepted as significant. ✓ Overall, group average gene expression changes were presented in most of the studies included in this review. These average values were calculated using all the individual results, i.e., adding together, both upregulation and downregulation changes. Very few studies included individual data.	○ **Results presentation: data summary and distribution (variability).** ✓ It is important that information about data (gene expression changes) normality is provided within the articles as well as to clearly describe the estimators used in the study. ✓ When providing group average and dispersion gene expression changes, it is probably best to opt for more robust estimators such as the median and the IQR unless the data follow a normal distribution. In this latter case, the mean and the SD should be used (the SEM is not a measure of dispersion) [102]. ✓ As a general tendency, the use of the 95% CI is recommended rather than the *p*-value. The 95% CI gives information both about the significance of the results and of the amount of change, i.e., ‘effect size’ [103]. ✓ Given the high variability in the gene expression responses, the interpretation of individual results rather than (or in addition to) group average responses should be implemented [4]. The gene expression upregulation and downregulation results within the test sample population should be differentiated and the distribution of volunteers displaying one type or another of response should be indicated. Also, those individuals not exhibiting a change in the gene should be separately stated. In this regard, it is important that we progress on the knowledge and understanding of where to establish the difference between a change (‘effect size’) and a no change (‘no effect’). Up to date, this is based merely on arbitrary cut-off values. As we progress on the knowledge of the gene expression regulation in response to bioactive compounds we shall be able to better establish what a meaningful change may be (‘effect size’) so that we can discern within the sample population who is responding and who is not responding to the treatment at the molecular level.
**Other relevant information needed to prove the relationship between the intake of bioactive compounds and gene expression changes and to increase the level of evidence supporting these molecular changes as a responsive mechanism underlying the health benefits of bioactive compounds in humans**	
○ **Bioavailability studies** ✓ Many of the gene expression studies included in this review did not perform appropriate parallel bioavailability studies of the bioactive compounds investigated.	○ **Bioavailability studies** ✓ We need to determine and quantify the specific bioactive compounds and/or derived metabolites in the cells or tissue samples where the gene expression is going to be analyzed. The interindividual variability in the bioavailability of these compounds can have a major impact on the gene expression differences [100].
○ **Association between gene expression changes and bioactive compounds and/or metabolites** ✓ Of those studies in which the metabolism and presence of certain compounds or metabolites was reported, very few indicated to have found some association (either negative or positive correlation) between the gene changes detected and the levels of the specific compound(s).	○ **Association between gene expression changes and bioactive compounds and/or metabolites** ✓ We need to search for any relationship(s) between the gene expression changes detected in the treated group and the presence/concentration of specific bioactive compounds and/or derived metabolites in the cells and tissue samples and/or in the circulating blood and/or in urine. ✓ Additional evidence of the relationship compound-gene change could be provided by dose/response studies (e.g., larger gene expression changes with increases in the concentration of a compound) and/or by showing the reversal of the gene expression changes in the absence of the compound (e.g., after a washout period).
○ **Changes at the protein/activity level** ✓ Only a few studies attempted to explore the potential association between the gene expression changes and changes in the protein levels with only half of them reporting some agreement between transcription and translation.	○ **Changes at the protein/activity level** ✓ To further progress on the understanding of the molecular responses and mechanisms implicated in the health benefits of bioactive compounds we need to implement more and better studies looking at the protein responses in parallel to the gene expression studies and try to confirm that the transcription regulation promoted by the specific bioactive compounds and/or metabolites is translated into active proteins.

## Supplementary Materials

The following are available online at http://www.mdpi.com/2072-6643/10/7/807/s1, Table S1: Characteristics of the human trials included in this review: study design, type of participants, control and intervention description, dose and duration of treatment, analyses and related bioavailability studies, Table S2: Gene expression experimental features extracted from the articles collected in this review: type of sample, preparation and description; RNA extraction, quality/purity analysis and reporting; reference gene applied, Table S3: Reported changes in gene expression levels in human tissues (as analyzed by RT-PCR) following intervention with diet, foods or products rich in bioactive compounds or with single compounds (↓green color, significant downregulation; ↑red color, significant upregulation, attributed to treatment either in comparison to baseline or to control; *p*-values are indicated, *p* < 0.05 are accepted as significant). The type of comparisons performed in the study, the way data are presented and whether there were any association(s) with bioactive metabolites and/or with protein quantity/activity are also included, Table S4: Summary of the expression changes in *TNF* as determined in human cell or tissue samples following intervention with diets, foods or derived products rich in bioactive compounds or with single bioactive compounds. The type and quality of the data presented in the study, estimation of or information about the variability as well as information about the association between the observed effects with the presence of bioactive metabolites and/or with the corresponding protein quantity/activity are indicated, Table S5: Summary of the expression changes in the *PPAR* family of genes as determined in human cell or tissue samples following intervention with diets, foods or derived products rich in bioactive compounds or with single bioactive compounds. The type and quality of the data presented in the study, estimation of or information about the variability as well as information about the association between the observed effects with the presence of bioactive metabolites and/or with the corresponding protein quantity/activity are indicated, Table S6: Summary of the expression changes in the *GPX* family of genes as determined in human cell or tissue samples following intervention with diets, foods or derived products rich in bioactive compounds or with single bioactive compounds. The type and quality of the data presented in the study, estimation of or information about the variability as well as information about the association between the observed effects with the presence of bioactive metabolites and/or with the corresponding protein quantity/activity are indicated.
