# Supplementary material for: Critical Evaluation of Gene Expression Changes in Human Tissues in Response to Supplementation with Dietary Bioactive Compounds: Moving Towards Better-Quality Studies
Source: Nutrients. 2018 Jun 22;10(7):807. doi: 10.3390/nu10070807 (PMC6073419; doi:10.3390/nu10070807)
Supplement: Supplementary file 1 [file nutrients-10-00807-s001.pdf]

Supplementary Material
'Critical Evaluation of Gene Expression Changes in Human Tissues in Response to Supplementation with Dietary Bioactive Compounds: Moving Towards Better-Quality Studies' by Biljana Pokimica and María-Teresa García-Conesa

Table S1. Characteristics of the human trials included in this review: study design, type of participants, control and intervention description, dose and duration of treatment, analyses and related bioavailability studies.

**Study Experimental Characteristics**						
Reference	Clinical trial design (RCT, crossover, parallel)	Participants (health status, gender)	C (Control description)	T (Treatment with bioactive compounds, products or diet)	Total daily dose, duration (d or h)[1]	Bioavailability studies: type of sample, compounds and (or) metabolites analysed, main results[2]
**Mix meals and diets**						
Persson I et al., 2000 [1]	Single arm	Healthy, men	C: not included	T: mix Veg	T: 250 g, 21 d	NR
Møller P et al., 2003 [2]	RCT, parallel, double blinded (regarding C_1 and C_2)	Healthy, mix	C_1: placebo tablet + energy drink (same amount of sugars as T)<br>C_2: tablet with antioxidants + minerals (same amount as T) + energy drink (same amount of sugars as T)	T: mix FruVeg	T: 600 g, 24 d	Plasma: (NS↑) *β-car*, T, C_2 (post- *vs* pre-)<br>(NC) VitC, T, C_2 (post- *vs* pre-)<br>(NS↓, 69%) VitC, *β*-car, C_1 (post- *vs* pre-)
Almendingen K et al., 2005 [3]	Randomized, crossover, single blinded	Healthy, mix	C: no proper control included (comparison between doses)	$T_{1,2}$: mix FruVeg	T_1: 300 g,<br>T_2: 750 g,<br>14 d	Plasma: ↑*α*-car, *β*-car, T_2 *vs* T_1 (post-)<br>(NS↑) Lyc, Lut, T_2 *vs* T_1 (post-) [4]<br>Urine: ↑Quer, Isor, Nar, Hes, Total flavonoids T_2 *vs* T_1 (post-), T_1 (post- *vs* pre-), T_2 (post- *vs* pre-)<br>↑Kaem, Tam, Eri, T_2 *vs* T_1 (post-), T_2 (post- *vs* pre-); (NC) Phlo [5]
Dragsted LO et al., 2006 [6]	RCT, parallel, double blinded (regarding C_1 and C_2)	Healthy, mix	C_1: placebo tablet + energy drink<br>C_2: tablet with vitamins + minerals (same amount as T) + energy drink	T: mix FruVeg	T: 600 g, 24 d	Plasma: ↓VitC, β-car, C_1 (post- *vs* pre-),<br>↓Lyc C_1, C_2 (post- *vs* pre-), ↑β-car, C_2 *vs* T (post-),C_2 *vs* C_1 (post-), ↑*α*-toc C_2 *vs* T (post-)<br>(NC) VitC, folate C_2 *vs* T (post-)<br>[7]
Di Renzo L et al., 2014 [8]	Randomized, crossover	Healthy, mix	C: baseline (includes comparison between the different groups)	T_1: Red wine<br>T_2: Med meal<br>T_3: T_1+T_2<br>T_4: McD meal<br>T_5: T_4+T_1	T_1: 250 mL,<br>T_2: 1 meal,<br>T_3: 250 mL T_1 + T_2,<br>T_4: 1 meal,<br>T_5: 250 mL T_1 + T_4,<br>4 h	NR
De Lorenzo A et	Randomized,	Healthy,	C: not included	T_1: Toc-enriched Med meal	$T_{1,2}$: 1 meal,	NR

al., 2017 [9]	crossover	mix	(includes comparison between T_1 and T_2)	T_2: western H-F meal	3 h	
Marques-Rocha JL et al., 2016 [10]	Single arm (part of RESMENA study [11])	MetS, mix	C: not included	T: hypocaloric Med-based diet	T: seven meals per day, Menu plan, 56 d	NR
**Foods and derived food products**						
Broccoli						
Gasper AV et al., 2007 [12]	RCT, crossover	Healthy, mix	C: water	T_1: high SFGluc broccoli drink (2295.9 ± 217.53 μmol/L), T_2: standard SFGluc broccoli drink (682.6 ± 113.30 μmol/L)	T_1: 150 mL, T_2: 150 mL, 6 h	Plasma: ↑SF metabolites, T_1 *vs* T_2 (post-), T_1 (max at 2h), T_2 (max at 1 h 30) Urine: ↑SF metabolites, T_1 *vs* T_2 (post-) [13]
Riedl MA et al., 2009 [14]	RCT, Parallel, Single blinded	Healthy, non-smokers mix	C: alfalfa sprout	$T_{1\text{-}8}$: broccoli sprout (SFGluc)	T_1: 25 g(~13 μmol SF), T_2: 50 g (~26 μmol SF), T_3: 75 g(~38 μmol SF), T_4: 100 g(~51 μmol SF), T_5: 125 g (~64 μmol SF), T_6: 150 g (~76 μmol SF), T_7: 175 g (~89 μmol SF), T_8: 200 g (~102 μmol SF) C: 200 g, 3 d	Serum: ↑SF, $T_{1\text{-}8}$ (post- *vs* pre-) Detected at 24 h after final dose ND at baseline
Yanaka A et al., 2009 [15]	RCT, parallel, double blinded	*H. pylori* infected, mix	C: alfalfa sprout	T: broccoli sprout (SFGluc)	T: 50 g, 24 h	Urine: ↑DTH, T (post- *vs* pre-), T *vs* C (post-) after 28 d and 56 d of 70 g SFG intake
Riso P et al., 2010 [16]	RCT, crossover	Healthy, smokers men	C: control diet	T: broccoli (SFGluc, Lut, β-car, VitC)	T: 250 g, 10 d	Plasma: ↑folate, ↑Lut, T (post- *vs* pre-) (NC) β-car
Atwell LL et al., 2015 [17]	Randomized, parallel (2-phases)	Healthy, non-smokers mix	C: no proper control included (includes comparison between T_1 and T_2)	T_1: broccoli sprout (SFGluc) T_2: myrosinase-treated broccoli sprout extract (SFGluc)	Phase I (single dose): T_1: 200 μmol SFGluc, T_2: 200 μmol SFGluc, 2 d Phase II (two doses in 12 h): T_1:2 x 100 μmol SFGluc, T_2:2x 100 μmol SFGluc, 2 d	Plasma: ↑SF metabolites, T_1, T_2 (post-), T_1 *vs* T_2 Urine: ↑SF metabolites, T_1, T_2 (post-), T_1 *vs* T_2
Doss JF et al., 2016 [18]	Parallel, open-label, phase 1	Sickle cell disease, mix	C: not included	$T_{1\text{-}3}$: broccoli (SFGluc)	T_1: 50 g, T_2: 100 g, T_3: 150 g, 21 d	Urine and complete metabolic profile analysed at the baseline and at the end of the study but results NR
Oils						
Camargo A et	Randomized,	MetS,	C: no proper control	T_1: high polyphenol olive	$T_{1,2}$: 40 mL,	NR

al., 2010 [19]	crossover,<br>double blinded	mix	included (comparison between T_1 and T_2)	oil<br>(398 ppm)<br>T_2: low polyphenol olive oil<br>(70 ppm)	4 h	
Konstantinidou V et al., 2010 [20]	RCT,<br>parallel	Healthy,<br>mix	C: habitual diet (includes comparison between T_1 and T_2)	T_1: Med diet + virgin olive oil (328 mg/kg polyphenols),<br>T_2: Med diet + washed virgin olive oil (55 mg/kg polyphenols)	$T_{1,2}$: no specific daily dose established, provided 15 L for participants and their families,<br>90 d	Urine: ↓Tyr, T_2 *vs* C (post-),<br>(NC) HTyr, T_2 *vs* C (post-),<br>↑Tyr, HTyr, T_1 *vs* T_2 (post-)
Chan JM et al., 2011 [21]	RCT,<br>parallel,<br>double blinded	Prostate cancer,<br>men	C: soy oil + olive oil + multivit	T_1: lycopene in soy oil + olive oil + multivit<br>T_2: fish oil + soy oil + multivit	T_1: 30 mg,<br>T_2: 3 g,<br>90 d	NR
Castañer O et al., 2012 [22]	Randomized,<br>crossover	Healthy,<br>men	C: no proper control included (comparison between T_1 and T_2)	T_1: high polyphenol olive oil<br>(366 mg/kg)<br>T_2: low polyphenol olive oil<br>(2.7 mg/kg)	T_1: 25 mL<br>(8.4 mg polyphenols),<br>T_2: 25 mL<br>(0.06 mg polyphenols),<br>21 d	Urine: ↑Tyr, HTyr, T_1 *vs* T_2 (post-)
Farràs M et al, 2013 [23]	Randomized,<br>crossover,<br>double blinded	Healthy with pre- or stage-I hypertension,<br>mix	C: no proper control included (comparison between T_1 and T_2)	T_1: high polyphenol olive oil (961 mg/kg)<br>T_2: moderate polyphenol olive oil (289 mg/kg)	T_1: 30 mL<br>(26.2 mg polyphenols),<br>T_2: 30 mL<br>(8 mg polyphenols),<br>5 h	Plasma: ↑HTyr metabolites,<br>T_1 (post- *vs* pre-), T_2 (post- *vs* pre-)<br>Max at 2 h post
Perez-Herrera A et al., 2013 [24]	Randomized,<br>crossover	Healthy obese,<br>non-smokers,<br>mix	C: no proper control included (comparison between different groups)	T_1: heated virgin olive oil<br>T_2: heated sunflower oil<br>T_3: heated T_2 + canola oil +dimethylpolysiloxane<br>T_4: heated T_2 + canola oil + olive antioxidants	$T_{1\text{-}4}$: 0.45 mL of oil/kg BW volunteer,<br>4 h	NR
Rangel-Zuñiga OA et al., 2014 [25]	Randomized,<br>crossover	Healthy obese,<br>non-smokers,<br>mix	C: no proper control included (comparison between different groups)	T_1: heated virgin olive oil<br>T_2: heated sunflower oil<br>T_3: heatedT_2 + canola oil +dimethylpolysiloxane<br>T_4: heated T_2 + canola oil + olive antioxidants	$T_{1\text{-}4}$: 0.45 mL oil/kg BW volunteer,<br>4 h	NR
Hernáez Á et al., 2015 [26]	Randomized,<br>crossover,<br>double-blinded	Healthy,<br>men<br>(sub-sample from the EUROLIVE study [27])	C: no proper control included (comparison between T_1 and T_2)	T_1: high polyphenol olive oil<br>(366 mg/kg)<br>T_2: low polyphenol olive oil<br>(2.7 mg/kg)	T_1: 25 mL (8.4 mg polyphenols)<br>T_2: 25 mL (0.06 mg polyphenols),<br>27 d	Urine: ↑Tyr, HTyr, T_1 (post- *vs* pre-),<br>T_1 *vs* T_2 (post-)

Martín-Peláez S et al ., 2015 [28]	Randomized, crossover, double blinded	Healthy, men (subsample from the EUROLIVE study[27])	C: no proper control included (comparison between T_1 and T_2)	T_1: high polyphenol olive oil (366 mg/kg)<br>T_2: low polyphenol olive oil (2.7 mg/kg)	T_1: 25 mL (8.4 mg polyphenols),<br>T_2: 25 mL (0.06 mg polyphenols),<br>21 d	Urine: ↑Tyr, ↑HTyr, T_1 (post- *vs* pre-),<br>T_1 *vs* T_2 (post-)<br>24 h
Kruse M et al., 2015 [29]	Randomized, parallel	Healthy obese, men	C: no proper control included (comparison between T_1 and T_2)	T_1: rapeseed/canola oil (MUFA, PUFA)<br>T_2: Olive oil (MUFA)	T_1: 50 g, T_2: 50 g,<br>28 d,<br>T_1: 25 g, T_2: 25 g,<br>4 h	Plasma: ↑n-3 (ALA, EPA), T_1 (post- *vs* pre-)<br>↓n-6/n-3 ratio, T_1 (post- *vs* pre-)<br>(NC) n-3, n-6 , T_2 (post- *vs* pre-),<br>(NC) DHA , T_1, T_2, (post -*vs* pre-)
Nuts						
González-Sarrías A et al., 2010 [30]	Randomized, parallel	Benign prostate hyperplasia or cancer, men	C: not consuming T_1 or T_2	T_1: walnuts (ETs),<br>T_2: pomegranate juice (ETs)	T_1: 35 g (202 mg ETs + 8 mg free EA),<br>T_2: 200 mL (265 mg ETs + 14 mg free EA),<br>3 d	Urine, plasma: Uro-A-gluc main metabolite detected. High interindividual variation in Uro metabolites, T_1 , T_2 (post-)<br>Prostate tissue: Detection of Uro-A-gluc, Uro-B-gluc, DMEA, T_1, T_2 (post-)
Hernández-Alonso P et al., 2014 [31]	RCT, crossover	Pre-diabetes, mix	C: control diet	T: control diet + pistachio	T: 57 g pistachios,<br>120 d	Plasma: ↑LZ , ↑γT, T *vs* C (post-),<br>T (post- *vs* pre-)
Donadio JLS et al., 2017 [32]	Single arm	Healthy, mix (from SU.BRA.NUT study [33])	C: not included	T: Brazil nut (Se)	T: 3-4 g (300 μg)<br>56 d	NR
Di Renzo L et al., 2017 [34]	RCT, crossover	Healthy, mix	C: no proper control included (includes comparison between T_1 and T_2)	T_1: McD meal+ raw hazelnuts<br>T_2: McD meal	T_1: 1 meal + 40 g<br>T_2: 1 meal,<br>3 h	NR
Beverages						
Guarrera S et al., 2007 [35]	Randomized, single arm (part of a RCT, parallel study)	Healthy smokers, men	C: not included	T: green tea, bilberry juice and soya products (flavonoids)	T: dose not reported (flavonoids intake estimated),<br>28 d	Urine: (NC) phenolics, T (post- *vs* pre-)
Volz N et al., 2012 [36]	Single arm	Healthy, men	C: not included	T: coffee (CGA, NMP)	T: 29.5 g,<br>28 d	NR
Boettler U et al., 2012 [37]	Single arm	Healthy, men	C: not included	T: coffee (CGA, NMP)	T: 29.5 g,<br>28 d	NR
Various						
de Pascual-Teresa S et al., 2003 [38]	RCT, crossover, single blinded	Healthy, mix	C: glucose + water	T_1: white onion + maltodextrin (low in Quer),<br>T_2: yellow onion	T_1: 368 g (11.2 mg of Quer aglycone content),<br>T_2: 368 g (306.9 mg of Quer aglycone content),	Plasma: ↑Quer metabolites (sulfated, glucuronide, methylated), T_2 (max at ~1 h)

				(high in Quer)	C: 25 g + 400 mL<br>3 h, 6 h	
Marotta F et al., 2010 [39]	Single arm	Healthy,<br>non-smokers<br>mix	C: not included	T: fermented papaya	T: 6 g (sublingual)<br>28 d	NR
Bertuccelli G et al., 2016 [40]	RCT,<br>parallel,<br>double blinded	Healthy with aging skin,<br>mix	C: antioxidant mix (*trans*-Res + selenium + VitE + VitC	T: fermented papaya	T: 9 g (sublingual)<br>C: 10 mg+60 μg+10 mg+50 mg,<br>90 d	NR
Ishikawa H et al., 2012 [41]	RCT,<br>parallel,<br>double blinded	Adenoma polyps,<br>mix	C: placebo	T: propolis (Atrepillin C, polyphenols)	T: 165 μmol Atrepillin C<br>150 μmol phenols,<br>90 d	NR
**Extracts and mixed compounds or supplements**						
Fruit extracts						
Nguyen AV et al., 2009 [42]	Randomized,<br>parallel,<br>open label,<br>phase I	Colon cancer,<br>mix	C: not included	T_1: Res + Quer<br>T_2: Res + Quer<br>T_3: grape powder*<br>T_4: grape powder*<br>*(Res, flavanols, flavans, anthocyanins, catechin)	T_1: 15.54 mg + 480 mg,<br>T_2: 3.886 mg + 120 mg,<br>T_3: 120 g (0.114 mg Res)<br>T_4: 80 g (0.073 mg Res)<br>14 d	NR
Weseler AR et al., 2011 [43]	RCT,<br>parallel,<br>double blinded	Healthy,<br>non-obese,<br>men	C: placebo	T: flavanols isolated from grape seeds	T: 200 mg,<br>56 d	NR
Barona J et al., 2012 [44]	RCT,<br>crossover,<br>double blinded	MetS,<br>men	C: placebo	T: grape powder	T: 46 g<br>(267 mg polyphenols),<br>28 d	NR
Tomé-Carneiro J et al., 2013 [45]	RCT,<br>parallel<br>triple blinded	Hypertensive with T2D,<br>men	C: placebo	T_1: grape extract<br>T_2: grape extract + Res	T_1: 151 mg polyphenols<br>T_2: 139 mg polyphenols + 8 mg Res<br>183 d (×1), 365 d (×2)	NR
Mallery SR et al., 2008 [46]	Single arm	Premalignant oral lesions *vs* healthy,<br>mix	C: not included	T: black raspberry gel	T: 2 g (lingual application)<br>42 d	NR
Kropat C et al., 2013 [47]	Parallel	Ileostomy probands *vs.* healthy,<br>women	C: not included	T: bilberry pomace extract (anthocyanins)	T: 10 g<br>(2.5 g anthocyanins),<br>8 h	Ileostomy effluent: within 1-2 h detected 30 ± 6% of ingested anthocyanins (a large % was degraded during passage of the upper gastrointestinal tract)
Knobloch TJ et al., 2016 [48]	Single arm<br>Phase 0	Oral squamous cell cancer,<br>mix	C: not included	T: black raspberry dried powder<br>(anthocyanins, EA, ET, Quer glycosides)	T: 4.3 g,<br>~14 d	Cancer tissue: C3R, C3X, C3G, C3S detected (post-)

Xie, L et al., 2017 [49]	RCT, parallel, double blinded	Healthy former smokers, mix	C: 0.2% beet juice concentrate	T: aronia berry extract (polyphenols)	T: 500 mg, 84 d	Plasma, urine: (NC) anthocyanins, phenolic acids, T *vs* C (post-), T *vs* C (pre-)
Nuñez-Sanchez MA et al., 2017 [50]	RCT, parallel	CRC, mix	C: not consumed T	$T_{1,2}$: pomegranate extract	T_1: 900 mg (144 mg/g of punicalagin, 4 mg/g of punicalin, 588 mg/g of EA derivatives), T_2: 900 mg (310 mg/g of punicalagin, 10.8 mg/g of punicalin, 56 mg/g of EA derivatives), ~14 d	Urine: High interindividual variability in Uro metabolites<br>Colon: No relationship between detected Uro metabolites and gene expression
Knott A et al., 2008 [51]	RCT, parallel, single blinded	Healthy, women	C_1: emulsion based on PEG-40-stearate<br>C_2: untreated area of skin	T: *Arctium lappa* fruit extract (0.25 % Arctiin) added to C (topical application)	T: 4 mg/cm^2 (1 mg Arctiin) of skin, 84 d	NR
Plant extracts						
Shrestha S et al., 2007 [52]	RCT, crossover, double blinded	Healthy, mix (menopausal status mix)	C: placebo	T: *Psyllium* + plant sterols	T: 10 g *psyllium* + 2.6 g plant sterols, 28 d	NR
Ghanim H et al., 2010 [53]	RCT, parallel	Healthy, mix	C: placebo	T: *Polygonum cuspidatum* extract (Res)	T: 40 mg, 42 d	NR
Marini A et al., 2012 [54]	Single arm	Healthy, postmenopausal women	C: not included	T: pine bark extract Pycnogenol (procyanidins)	T: 75 mg , 84 d	NR
Carrera-Quintanar L et al., 2015 [55]	RCT, parallel, single blinded (+exercise training routine)	Healthy sport practising, men	C: placebo	T_1: *Lippia citriodora* extract<br>T_2: Almond beverage (+VitE + VitC)<br>T_3: T_1 +T_2	T_1: 1.2 g,<br>T_2: 250 mL (+ 25 mg+ 75 mg),<br>T_3: 0.55 g T_1+250 mL T_2<br>21 d	NR
Turowski JB et al., 2015 [56]	Single arm	Cystic fibrosis *vs* healthy, mix	C: not included	T: flaxseed (fiber + lignan phenolics + n-3 fatty acids)	T: 40 g, 28 d	Plasma: Classification based on high enterolignan and low enterolignan levels at 14 d<br>↑ED, ↑EL, in high-lignan group (post- *vs* pre-)
Oil derived products						
Plat J and Mensik RP, 2001 [57]	RCT, parallel, double blinded	Healthy, mix	C: rapeseed oil based margarine and shortening	T_1: vegetable oil-based plant stanol esters mix added to C<br>T_2: wood-based plant stanol esters mix added to C	T_1: ~3.8 g stanol esters,<br>T_2: ~4.0 g stanol esters<br>C: < 0.1 g stanol esters<br>56 d	Serum: ↓Sit, Cam (reduced cholesterol absorption), T_{1+2} *vs* C (post-)
Crespo MC et	RCT,	Healthy,	C: placebo	$T_{1,2}$: Olive mill waste water	T_1: 5 mg,	NR

al., 2015 [58]	crossover, double blinded	mix		extract Hytolive (enriched in HTyr)	T_2: 25 mg, 7 d	
Boss A et al., 2016 [59]	RCT, parallel, double blinded	Healthy, men	C: placebo (glycerol +sucrose, no polyphenols)	T: olive leaf extract (oleuropein, HTyr)	T: 20 mL (estimated to be ~17 mL, ~121.8 mg oleuropein, ~6.4 mg of HTyr), 56 d	NR
Daak AA et al., 2015 [60]	RCT, parallel, double blinded	Sickle cell disease patients, mix	C_1: capsules containing high oleic acid (41%) blend<br>C_2: hydroxyurea	T: capsules containing n-3 PUFA (EPA, DHA)	T: 555.6-833.4 mg DHA + 78-117 mg of EPA (doses according to age),<br>C_2: >20 mg/kg<br>365 d	Red blood cells: ↑EPA, ↑DHA, ↓n-6 PUFA, ↓AA, T (post- *vs* pre-)<br>↓EPA, ↑n-6 PUFA, C (post- *vs* pre-)
Labonté M-E et al., 2013 [61]	RCT, crossover double blinded	T2D, men	C: placebo (50/50 blend of corn and soybean oil)	T: fish oil (EPA + DHA)	T: 5 g of fish oil (3 g of EPA (64%= 1.92g) + DHA (36%=1.08g))<br>56 d	Plasma phospholipid FA: ↑n-3 (EPA, DHA, DPA), ↓ n-6 (AA, DGLA), T *vs* C
Jamilian M et al., 2018 [62]	RCT, parallel, double blinded	GDM, women	C: placebo	T: fish oil (EPA + DHA)	T: 2000 mg (360 mg EPA + 240 mg DHA) , 42 d	NR
Mixed compounds and products						
Marini A et al., 2014 [63]	RCT, parallel, double blinded	Polymorphic light eruption photo-dermatosis, mix (+ UVA induced photo-dermatosis)	C: placebo (microcrystalline cellulose)	T: lycopene + β-car + probiotic (*Lactobacillus johnsonii)*	T: 1 capsule (2.5 mg + 4.7 mg + 5.10^8 cfu), 84 d	NR
Farris P et al., 2014 [64]	Single arm	Healthy, women	C: not included	T: Res + Bai + vitE (topical application)	T: 2 mg/cm^2 of skin, 84 d	Skin: Res, Bai detected in epidermis and dermis
Radler U et al. 2011 [65]	RCT, parallel, double blinded	Obese, hyperlipidemic, mix	C: low-fat yoghurt + vitC + vitE	T: low-fat yoghurt + grapeseed extract + fish oil + phospholipids + Carn + vitC + vitE	T: 250 g yoghurt +162 mg polyphenols + 200 mg n-3 PUFA + 800 mg phospholipids + 1 g Carn + 120 mg VitC + 20 mg VitE, 84 d	Urine: ↑Carn, ↑acyl-Carn<br>T (post *vs* pre), T *vs* C (post-)
**Single bioactive compounds**						
Frommel TO et al., 1994 [66]	Parallel	Prior history of CRC /polyps and healthy, mix	C_1: placebo<br>C_2: (healthy, no T or C_1)	T: β-car	T: 30 mg, 90 d	Serum, colon tissue: ↑ β-car, T (post- *vs* pre-)
Vors C et al., 2017 [67]	RCT, crossover, double blinded	Healthy with abdominal obesity,	C: corn oil (no EPA or DHA)	T_1: EPA<br>T_2: DHA	T_1: 2.7 g EPA,<br>T_2: 2.7 g DHA,<br>C: 3 g	Plasma phospholipids: ↑EPA in T_1 *vs* T_2 or C, ↑DHA in T_2 *vs* C or T_1<br>[68]

		mix, (subsample of ComparED study [68])			70 d	
Yoshino J et al., 2012 [69]	RCT, parallel, double blinded	Healthy, non-obese (lean & overweight) women (post-menopausal)	C: placebo	T: Res	T: 75 mg, 84 d	Plasma: ↑Res , ↑dihydroRes, T (post- *vs* pre-) , max at ~2h
Poulsen MM et al., 2013 [70]	RCT, parallel, double blinded	Healthy obese, men	C: placebo	T: Res	T: 1500 mg, 28 d	Urine: ↑Res metabolites, T *vs* C (post-)
Olesen J et al., 2014 [71]	RCT, parallel, double blinded (+ exercise training in 2 of 4 groups)	Healthy physically inactive, men	C: placebo	T: Res	T: 250 mg, 56 d	NR
Chachay VS et al., 2014 [72]	RCT, parallel, double blinded	Overweight/ obese, non-alcoholic fatty liver disease, men	C: placebo	T: Res (from *Polygonium cuspidatum*)	T: 3000 mg, 56 d	Plasma pharmacokinetic study: ↑Res after 60 min of oral dose (1.5 g). High interindividual variation (54%)
Yiu EM et al., 2015 [73]	Not randomized, parallel, open-label	Friedrich ataxia, mix	C: not included	$T_{1,2}$: Res	T_1: 1 g, T_2: 5 g, 84 d	Plasma pharmacokinetic study: ↑Res and Res metabolites (glucuronides, sulfates) after 45 min and 90 min of oral doses (0.5 and 2.5 g)
Mansur AP et al., 2017 [74]	Randomized, parallel	Healthy, overweight, mix	C: caloric restriction (1000 calories/d)	T: Res	T: 500 mg, 30 d	NR
Morrow DMP et al., 2001 [75]	Crossover (no wash-up period included)	Healthy, non-smokers, men	C: flavoured drink	T: Quer added to C	T: 30 mg, 14 d	NR
Nieman DC et al., 2007 [76]	RCT, parallel, double blinded, (+ exercise training)	Healthy trained cyclists, men	C: placebo (Tang powder)	T: Quer	T: 1000 mg, 24 d (21 d before+ 3 d of training)	Plasma: ↑Quer, T *vs* C (post, at 21 d)
Boesch-Saadatmandi C et al., 2009 [77]	RCT, crossover, double blinded	High CVD risk phenotype, mix	C: placebo	T: Quer	T: 150 mg, 42 d	Plasma : ↑Quer after supplementation and in comparison to placebo
Nelson DM et al., 2011 [78]	Single arm	Acute myeloid leukaemia, mix	C: not included	T: flavopiridol	T: 50-100 mg/m^2 (bolus infusion), 3 d	NR
Lazarevic B et	RCT,	Prostate cancer,	C: placebo	T: genistein	T: 30 mg,	NR

al., 2012 [79]	parallel,<br>double blinded,<br>phase-2	men			33 d	
Kerksick CM et al., 2013 [80]	RCT,<br>parallel,<br>double blinded,<br>(+ exercise training)	Healthy non-resistance-trained,<br>men	C: placebo (glucomannan)	T_1: NAC<br>T_2: EGCG	T_1: 1800 mg<br>T_2: 1800 mg,<br>C: 1000 mg<br>14 d	NR
Most J et al, 2015 [81]	RCT,<br>crossover,<br>double blinded,<br>(postprandial response)	Healthy,<br>overweight,<br>mix	C: placebo + liquid test meal (partially hydrolysed cellulose)	T: EGCG + liquid test meal	T: 282 mg<br>3 d	Plasma: ↑EGCG (maximum concentration of EGCG 1 h after intake and then decline during 6 h)
Koosirat C et al., 2010 [82]	RCT,<br>parallel	Chronic gastritis,<br>mix	C: Omeprazole + Amoxicillin + Metronidazole	T: Turmeric tablet (containing 40 mg Cur)	T: 120 mg,<br>28 d,<br>C: 40 mg + 2g + 1600 mg,<br>7 d	NR
Klickovic U et al., 2014 [83]	Single arm,<br>phase-1 pilot study,<br>open-label	Healthy,<br>men	C: not included	T: Cur + Bioperine (black pepper extract containing the alkaloid piperine added to improve Cur bioavailability)	T: 12 g + 5 mg<br>2 d	Plasma: Cur ND (post-, pre-)

[1]Unless otherwise indicated, dose and duration were the same for C and T; studies classified as acute (≤ 24 h); [2]Bioavailability results presented as: sample where the compounds have been detected, ↑increase or ↓decrease, comparison where the change has been detected.

Table abbreviations (in alphabetical order): AA, arachidonic acid; ALA, alpha linolenic acid; α-car, α-carotene; α-toc, α-tocopherol; γT, γ-tocopherol; β-car, β-carotene; Bai, baicalin; BW, body weight; C, control; C3G, cyanidin-3-glucoside; C3R, cyanidin-3-rutinoside; C3S, cyaniding-3-sambubioside; C3X, cyaniding-3-xylosylrutinoside; Cam, campesterol; Carn, carnitine; CGA, chlorogenic acid; cfu, colony-forming unit; CRC, colorectal cancer; Cur, curcumin; CVD, cardiovascular disease; d, days; DGLA, dihomo-γ-linolenic acid; DHA, docosaxehaenoic acid; DMEA, dimethyl ellagic acid; DPA, docosapentanoic acid; DTH, dithiocarbamates; EA, ellagic acid; ED, enterodiol; EGCG, epigallocatechin gallate; EL, enterolactone; EPA, eicosapentaenoic acid; Eri, eriodictyol; ETs, ellagitannins; FruVeg, fruits and vegetables; GDM, gestational diabetes mellitus; h, hours; Hes, hesperetin; H-F, high-fat; HTyr, hydroxytyrosol; Isor, isorhamnetin; Kaem, kaempferol; Lut, lutein; Lyc, lycopene; LZ, lutein-zeaxantin; McD, Macdonald; Med, Mediterranean; MetS, metabolic syndrome; MUFA, monounsaturated fatty acids; multivit, multivitamin; NAC, N-acetyl-cysteine; Nar, naringenin; NC, no change; ND, not detected; NMP, *N*-methylpyridinum; NR, not reported; NS, not significant; n-3, omega-3; n-6, omega-6; PEG, polyethylene glycol; Phlo, phloretin; post-, after treatment; pre-, baseline or before treatment; PUFA, polyunsaturated fatty acids; Quer, quercetin; RCT, randomized control trial; Res, resveratrol; Se, selenium; SF, sulforaphane; SFGluc, sulforaphane glucosinolates; Sit, sitosterol; T, treatment; Tam, tamarixetin; T2D, type 2 diabetes; Tyr, tyrosol; Uro, urolithin; Uro-A-gluc, urolithin-A glucuronide; Uro-B-gluc,urolithin-B glucuronide; UVA, ultraviolet; Veg, vegetables; VitC, vitamin C; VitE, vitamin E.

Table S2. Gene expression experimental features extracted from the articles collected in this review: type of sample, preparation and description; RNA extraction, quality/purity analysis and reporting; reference gene applied.

Reference	**Sample/RT-PCR protocols**				
	Sample description	Sample preparation, characterization, storage conditions	RNA isolation protocol, storage conditions	RNA quantity & quality analyses (Abs $_{260/280}$ or RIN values)	Reference gene(s)
**Blood**					
Almendingen K et al., 2005 [3]	Blood	Fasting blood samples collected at the baseline and the end of the study. Processed immediately after sampling.	RNA isolated from fresh blood using total RNA Chemistry and ABI Prism 6700t Automated Nucleic Acid Workstation. DNase step NR.	NR	*GAPDH*
Guarrera S et al., 2007 [35]	Blood	Blood samples collected at the baseline and the end of the study, using PAXgene Blood RNA tubes.	Total RNA isolated using a column affinity procedure (PAXgene blood RNA kit). DNase step included.	NR	*ACTB*
Doss JF et al., 2016 [18]	Blood	Blood samples collected at the baseline and the end of the study, using PAXgene Blood RNA tubes.	RNA isolated from blood using PAXgene Blood miRNA Kit. DNase step included.	NR	*GAPDH*
Atwell LL et al., 2015 [17]	Blood	Blood samples collected in EDTA vacutainers before and at 3, 6, 12, 24, and 48 h after treatment. Samples preserved in PAXgene Blood RNA tubes.	Total RNA isolated using the PAXgene Blood miRNA kit. DNase step NR.	NR	*GAPDH*
Di Renzo L et al., 2014 [8]	Blood	Fasting blood samples collected before and after each treatment using PAXgene Blood RNA tubes and stored at -80 ºC until RNA extraction.	Total RNA isolated using PAXgene Blood miRNA Kit. DNase step NR.	Yes (quantity and quality assessed by spectrophotometer and agarose gel) (values NR)	*GAPDH, ACTB, HPRT1, B2M* (average value)
Weseler AR et al., 2011 [43]	Blood	Blood collected at the baseline, in the middle (28 d) and at the end of the study, added to RNALater and stored at -80 ºC.	Total RNA isolated from blood using RiboPure-Blood kit. DNase step included.	Yes (quantity and quality by spectrophotometer) (values NR)	*GAPDH*
De Lorenzo A. et al., 2016 [9]	Blood	Fasting blood samples at baseline and 3-h postprandial samples. No further information.	No information regarding RNA isolation. DNase step NR.	NR	NR
Vors C et al., 2017 [67]	Blood	Fasting blood samples collected after each treatment using PAXgene Blood RNA tubes.	Total RNA isolated from whole blood using the PAXgene RNA kit. DNase step NR.	NR	*GAPDH* (*G6PD, HPRT1* also tested but not used due to higher variability)
Donadio JLS et al., 2017 [32]	Blood	Blood samples collected at baseline, 28 d and 56 d of supplementation, and 28 d and 56 d after the end of supplementation. No further description.	Total RNA extracted from whole blood using Ribopure Blood Kit. DNase step NR.	Yes (quantity and quality by NanoDrop) (values NR)	*GAPDH*

Di Renzo L et al., 2017 [34]	Blood	Fasting blood samples at baseline and 3-h postprandial collected using PAX gene Blood RNA Tubes, and stored at -80 ºC.	RNA isolated using PAX gene Blood miRNA Kit.<br>DNase step NR.	Yes (quantity and quality assessed by spectrophotometer and agarose gel)<br>(values NR)	*ACTB, B2M, GAPDH, HPRT1, RPLP0*, included in the PCR-array (no further specification)
**Peripheral blood isolated immune cells[1]**					
Møller P et al., 2003 [2]	White blood cells	Fasting blood samples collected at baseline and at days 9, 16, and 24 during study, and 28 d after treatment, using EDTA tubes. No clear information about leukocytes isolation (method based on [84])	Total RNA isolated from full blood using the PAXgene blood RNA isolation kit but gene expression results in leukocytes.<br>DNase step NR.	NR<br>(low in some samples, values NR)	*18S rRNA*
Dragsted LO et al., 2006 [6]	White blood cells	Fasting blood samples collected before, during and after treatment. No clear information about leukocytes isolation (method based on [84])	No information regarding RNA isolation from leukocytes in the article.<br>DNase step NR	NR	*18S rRNA*
Farràs M et al, 2013 [23]	White blood cells	Blood samples collected before and 5 h after treatment using PAXgene tubes, kept for 2 h at room temperature then stored at -80 ºC. No information on isolation of leukocytes from blood or further characterization.	Total RNA isolated from leukocytes using PAXgene extraction kit by a liquid–liquid method.<br>DNase step NR	Yes (quality assessed by Agilent technology)<br>(values NR)	NR
Daak AA et al., 2015 [60]	White blood cells	Fasting blood samples collected at the baseline and the end of the study using EDTA tubes. Unclear description of cells isolation.	RNA isolated from peripheral blood cells using RNAqueous Kit.<br>DNase step NR.	Yes (quality by 2% agarose gel)<br>(values NR)	*GAPDH*
Marques-Rocha JL et al., 2016 [10]	White blood cells	Fasting blood samples collected in EDTA tubes at the baseline and at the end of the study. Cells isolated by centrifugation and frozen at -80 ºC (WBC in buffy coat).	Total RNA extracted using the Trizol method.<br>DNase step NR	Yes (quantity and quality by NanoDrop)<br>(values NR)	*GAPDH*
Mansur AP et al., 2017 [74]	White blood cells	Blood collected at the baseline and at the end of the study. No description of sample preparation or characterization indicated.	Total RNA isolated from peripheral leukocytes using the Trizol reagent.<br>DNase step NR.	NR	*GAPDH*
Plat J and Mensik RP, 2001 [57]	Mononuclear cells	Fasting whole blood samples collected at the end of study using EDTA tubes. PBMC isolated using Histopaque-1077, washed with a sterile, DEPC-treated 0.9% NaCl solution, resuspended in DEPC-treated solution, stored at -20 ºC until RNA isolation.	RNA isolated within 7 d of storage by chloroform extraction and isopropanol precipitation, and stored in DEPC ethanol at -80°C.<br>DNase step NR.	Yes (quantity by spectrophotometer at 260 nm; quality by 0.8% agarose gel)<br>(values NR)	*AW109* (competitive RT-PCR in the presence of this RNA competitor)
Konstantinidou V et al., 2010 [20]	Mononuclear cells	Fasting blood samples collected at the baseline and the end of the study. Protocols based on previous work and not included here.	Total RNA isolated from PBMC using a liquid-liquid method. Protocol based on previous work and not included here.<br>DNase step NR.	Yes (quantity and quality assessed but methods not indicated)<br>(values NR)	*GAPDH*
Riso P et al., 2010 [16]	Mononuclear cells	Fasting whole blood samples collected at the baseline and the end of the study using microtubes with heparin. PBMC isolated from	Liquid-liquid method. No information about RNA isolation.<br>DNase step NR.	NR	*18S rRNA*

		whole blood using Histopaque -1077. Cells resuspended in a solution containing 50% foetal bovine serum, 40% culture medium and 10% dimethyl sulphoxide and stored at -80 ºC.			
Shrestha S et al., 2007 [52]	Mononuclear cells	Whole blood samples collected after treatment. PBMC isolated from whole blood by the method of Böyum [85] using Histopaque-1077. Cells re-suspended in TRIS buffer and frozen at -80 ºC.	Total RNA extracted from PBMC by the method of Chomczynski and Sacchi [86], slightly modified by using isopropyl alcohol for RNA precipitation (Trizol method). DNase step included.	NR	*GAPDH*
Boss A et al., 2016 [59]	Mononuclear cells	Fasting whole blood samples collected at the baseline and the end of the study. PBMC extracted from blood samples by Ficoll–Paque density gradient centrifugation and stored until RNA extraction.	RNA extracted using the RNeasy Plus Mini Kit. DNase step NR.	Yes (quantitation by NanoDrop; quality by Bioanalyzer) (RIN > 8)	*GAPDH*, *ACTB* (combined values)
Klickovic U et al., 2014 [83]	Mononuclear cells	Blood samples collected before and at 2.5, 5, 7.5, 10, 24, and 48 h after treatment, using EDTA tubes. PBMC isolated from blood using Ficoll-Plaque prefilled tubes.	RNA isolated from cell pellets using lysis buffer. No further description of extraction method. DNase step NR.	NR	*18S rRNA*
Hernáez Á et al., 2015 [26]	Mononuclear cells	Fasting blood samples collected before and after treatment. Cell isolation protocol not indicated.	Total RNA isolated by means of a liquid-liquid method. DNase step NR.	Yes (quantity and quality checked but method not indicated) (values NR)	*GAPDH* (CV=0.98% in C)
Barona J et al., 2012 [44]	Mononuclear cells	Fasting blood samples collected before and after treatment using EDTA tubes. PBMC isolated from blood using Histopaque-1077 according to method of Böyum [85]. Total and relative numbers of PBMC analyzed but not reported.	Total RNA isolated from PBMC. Solution in phosphate buffer followed by extraction with Trizol [87]. DNase step included.	NR	*GAPDH*
Ghanim H et al., 2010 [53]	Mononuclear cells	Fasting blood samples collected in Na-EDTA tubes, at baseline and at 7, 21 and 42 d of treatment. Cells isolation by centrifugation in Lympholyte medium. It indicates the separation between MNC suspensions and polymorphonuclear cells (>95% purity).	Total RNA isolated using the RNAqueous4PCR Kit. DNase step NR.	NR	*ACTB, UBC, PPIA* (three genes used)
Martín-Peláez S et al., 2015 [28]	Mononuclear cells	Fasting blood samples collected at baseline and at the end of the study, stored at -80 ºC until analysis. Protocol based on previous work.	RNA extraction based on a previous reference. Not explained in the article. DNase step NR.	Yes (based on previous work) (values NR)	*GAPDH*
Camargo A et al., 2010 [19]	Mononuclear cells	Blood samples collected in EDTA tubes 4 h after treatment. Stored in ice water in the dark. PBMC isolated within 2 h using Ficoll gradient centrifugation, harvested in PBS buffer and stored in liquid N_2 at -80 ºC prior to RNA	Total RNA extracted from PBMC using TRI Reagent. RNeasy MiniElute Cleanup Kit included. DNase step NR.	Yes (quantitation by NanoDrop; quality assessed by 1.6% agarose gel) (intact bands for 18S and	*RPL13A*

		extraction.		28S rRNA on agarose gel)	
Rangel-Zuñiga OA et al., 2014 [25]	Mononuclear cells	Blood samples collected before , 2 h and 4 h after treatment. PBMC isolated from blood. Protocol based on previous work.	RNA isolated from cells based on previous work.<br>DNase step NR.	NR	*GAPDH*
Castañer O et al., 2012 [22]	Mononuclear cells	Blood samples collected before and after treatment. No information about cells isolation protocol.	Total RNA isolated from PBMC by a liquid-liquid method.<br>DNase step NR.	Yes (methods not indicated)<br>(values NR)	NR
Crespo MC et al., 2015 [58]	Mononuclear cells	Fasting blood samples collected before and after treatment using heparinized tubes, PBMC isolated within 2 h by centrifugation using Histopaque-1077. PBMC stored in Qiazol at -80 ºC prior to RNA extraction.	Total RNA extracted and purified from homogenized PBMC using miRNeasy minikit<br>DNase step included.	Yes (quantitation by NanoDrop; quality by Bioanalyzer)<br>(values NR)	*ACTB*<br>(stability tested for *GAPDH, ACTB, RPLPO* by Normfinder)
Kropat C et al., 2013 [47]	Mononuclear cells	Blood samples collected before and 1, 2, 4 and 8 h after treatment using EDTA tubes. PBMC isolated using Hystopaque-1119, and stored in RNAlater at -80 ºC.	RNA isolated using RNeasy Mini Kit.<br>DNase step NR.	NR	*GAPDH*<br>(*ACTB* also tested)
Perez-Herrera A et al., 2013 [24]	Mononuclear cells	Blood samples collected using EDTA tubes before and 2 and 4 h after treatment. PBMC isolated from blood by centrifugation using Ficoll gradient, stored as a dry pellet prior to RNA isolation.	RNA isolated using Tri Reagent. Stored at -80 ºC.<br>DNase step included.	Yes (quantity by spectrophotometer; quality by agarose gel)<br>(values NR)	*GAPDH*
Chachay VS et al., 2014 [72]	Mononuclear cells	Blood collected in heparin tubes at baseline, at week 1 and at the end of study. PBMC isolated by centrifugation using Ficoll gradient. Cells quantified and stored at -80 ºC.	Total RNA isolated from PBMC using RNeasy kit.<br>DNase step NR.	Yes (quantity and quality determined by Nanodrop)<br>(values NR)	*ACTB*
Yiu EM et al., 2015 [73]	Mononuclear cells	Methods as described in previous work. Not explained in the article.	RNA isolation not described, based on previous work.<br>DNase step NR.	NR	NR
Xie, L et al., 2017 [49]	Mononuclear cells	Whole blood samples collected in EDTA tubes at the baseline and at the end of study. PBMC isolated from whole blood cells using Ficoll-Paque premium and density gradient centrifugation. Cells collected in PBS, then in ice-cold fetal bovine serum and finally in cryopreservation media before storage at -80 ºC in a cryotank. Cell quantitation but no further characterization of the composition.	RNA isolated from pelleted PBMC with Trizol reagent.<br>DNAse step included.	NR	*GAPDH*
Radler U et al., 2011 [65]	Mononuclear cells	Fasted venous blood sample. PBMC enriched directly from blood by density gradient centrifugation (Ficoll-Hypaque). No description of cell composition. Cells stored at -80 ºC.	Stabilization reagent added to samples after thawing. Total RNA extracted and mRNA isolated using a kit for blood/bone marrow.<br>DNase step NR.	NR	*B2M, ACTB*<br>(both genes used)
Tomé-Carneiro J et	Mononuclear	Fasted blood collected in heparinized tubes. At	Total RNA extracted using the All-Prep	Yes (quantity and	*GAPDH*

al., 2013 [45]	cells	baseline, 6 and 12 months of treatment. Cells isolated by density gradient (Histopaque-1077), lysed in RLT buffer and stored -80 ºC. Protocol performed during the same time gap (morning and within 1 h). Cells counts provided: lymphocytes (84.4 ± 2.9%), monocytes (13.0 ± 3.2%), granulocytes (2.2 ± 1.4%)	DNA/RNA/proteins Mini kit DNase step NR.	quality by NanoDrop and Bioanalyzer) (Abs $_{260/280}$ 1.8-2.1, RIN >8.5)	
Jamilian M et al., 2018 [62]	Mononuclear cells	Fasting blood samples collected at baseline and at the end of the study (42 d). PBMC isolated from blood using 50% Percoll. Cells counted and viability tested by Trypan blue but no information provided.	RNA extracted using RNX-plus kit. RNA kept at -20 ºC. DNase step NR.	Yes (quantiy and purity by spectrophotometer) (Abs $_{260/280}$ 1.7-2.1)	*GAPDH*
Nelson DM et al., 2011 [78]	Leukemic blasts	Blood samples collected before and 2 h after the infusion. Blasts enrichment (≥70%) by Ficoll-Paque density gradient separation.	Total RNA isolated from the blasts using RNeasy Mini Kit. DNase step NR	Yes (quantity and quality by NanoDrop) (some samples had degraded RNA or low quantity)	*RPLP0, ACTB, B2M* (different reference genes used for the analysis of different genes)
Persson I et al., 2000 [1]	Lymphocytes	Blood samples collected before and after treatment. Lymphocytes isolated from blood by centrifugation. No further description or characterization.	Total RNA isolated from lymphocytes using Trizol LS reagent Kit. DNase step NR.	NR	*ACTB*
Morrow DMP et al., 2001 [75]	Lymphocytes	Fasting blood samples collected at baseline, after placebo, after treatment, and 35 d after the end of study. Peripheral lymphocytes isolated from blood using density gradient centrifugation, then stored in DNA–RNA stablisation reagent for blood/bone marrow at -70 ºC.	Total RNA isolated from lymphocytes using GlassMax RNA Microisolation Spin Cartridge System. DNase step NR.	Yes (quantity and quality by GeneQuant II RNA DNA calculator and gel analysis) (values NR)	*GAPDH*
de Pascual-Teresa S et al., 2003 [38]	Lymphocytes	Blood samples collected before and 3 h and 6 h after treatment. Lymphocytes immediately isolated from blood samples by centrifugation, cells re-suspended and counted.	Total RNA inmediately isolated from cells using RNeasy mini kit. RNase-free water and RNase inhibitor included. RNA stored at -70 ºC. DNase step NR.	Yes (quantity determined by Ribogreen) (values NR)	*GAPDH*
Boettler U et al., 2012 [37]	Lymphocytes	Venous blood samples collected at 4 time points (28 d before baseline, at baseline, at the end of the study and 28 d after the end of the study) in EDTA tubes and stored at room temperature until the individual sampling period was completed. Lymphocytes isolated from blood (protocol referenced).	Total RNA isolated from lymphocytes using Rneasy Mini Kit. DNase step NR.	NR	*ACTB*
Volz N et al., 2012 [36]	Lymphocytes	Blood samples collected at baseline and at at 4 time points (28 d before baseline, at baseline,	Total RNA extracted using RNeasyMini Kit. DNase step NR.	NR	*ACTB*

		at the end of the study and 28 d after the end of the study) using sodium heparin tubes. Immediately after, lymphocytes were isolated by centrifugation using Histopaque-1077 and stored in RNAlater stabilization reagent.			
Hernández-Alonso P et al., 2014 [31]	Lymphocytes	Fasting blood samples collected at the baseline and the end of the study.<br>Cell isolation (unclear) in heparin tubes.<br>Process and characterization not described.	Total RNA isolated from blood using Tempus Spin RNA Isolation Kit but gene expression in lymphocytes.<br>DNase step NR.	Yes (quantity and purity by NanoDrop)<br>(values NR)	*HPRT1, YWHAZ* (selected and used based on Genevestigator)
Carrera-Quintanar L et al., 2015 [55]	Lymphocytes, neutrophils	Fasting blood samples collected in EDTA tubes, at baseline and at the end of the study. Lymphocytes and neutrophils isolated using an adaptation of the method by Böyum [88]. No further description of the samples.	Total RNA isolated from lymphocytes and neutrophils using Tripure extraction kit.<br>DNase step NR.	NR	*RPLP0*<br>(*36B4 rRNA*)
Marotta F et al, 2010 [39]	Neutrophils	Fasting blood samples collected in heparine tubes at the baseline, in the middle (14 d) and, at end of the study. Immediately after, neutrophiles were extracted using Ficoll–Paque centrifugation. Enrichment and viability of cells tested (>90% neutrophils 90% viable).	Total RNA extracted by Trizol.<br>DNase step included.	Yes (quantity and quality by spectrophotometer and agarose gel)<br>(values NR)	*GAPDH, HMBS* (different reference genes used for the analysis of different genes)
Yanaka A et al., 2009 [15]	Polymorpho-nuclear granulocytes	Blood samples collected before and 24 h after treatment. Polymorphonuclear granulocytes purified from blood samples using Polymorphprep (protocol based on previous work). No other detail included.	RNA isolation not described.<br>DNase step NR.	NR	NR
Boesch-Saadatmandi C et al., 2009 [77]	CD14+ monocytes	Fasting venous blood samples collected at baseline and at the end of the study using EDTA tubes. Monocytes (CD14 positive) isolated by density centrifugation with LymphoPrep, then positively selected with magnetic beads, protocol referenced.	RNA isolated from monocytes using the RNeasy Kit.<br>DNase step NR.	Yes (quantitation by spectrophotometer; quality not indicated)<br>(values NR)	*ACTB*
Nieman DC et al., 2007 [76]	White blood cells, skeletal muscle	Whole blood collected in EDTA tubes at at d 22, 23, 24 (before and after exercise, but all time use of quercetin). Leukocytes isolated by centrifugation.<br>Skeletal muscle biopsy samples obtained at d 22 and 24 (before and after exercise, but all time use of quercetin) from the leg using the percutaneous needle biopsy procedure modified to include suction, under local anesthesia. Biopsy samples divided, immediately frozen and homogenized with a polytron in liquid N_2 and stored at -80 ºC prior to analysis.	Leukocytes RNA isolated using QIAampRNA Blood Mini Kit Protocol. Contaminants were washed away and then, RNA eluted in RNase-free water.<br>Total RNA extracted from biopsies using the guanidine thiocynate method with Trizol Reagent , then dissolved in diethylpyrocarbonate-treated water.<br>DNase step NR.	Yes (quantity by spectrophotometer at 260 nm)<br>(values NR)	*18S rRNA*

Gastrointestinal tissue samples					
Mallery SR et al., 2008 [46]	Oral intra-epithelial neoplasia and normal ventral-lateral tongue	Excisional biopsies obtained at the beggining (from subjects with neoplasia) and at the end of the study (from both neoplasia and normal tissues), snap frozen.	Total RNA isolated using Absolutely RNA Miniprep Kit<br>DNase step NR	Yes (quantity by NanoDrop, quality by Bioanalyzer)<br>(values NR)	*GUSB*
Turowski JB et al., 2015 [56]	Buccal swabs	Buccal samples collected using a MasterAmp Buccal Swab Brush and deposited into RNAlater, stored at 4 ºC first and then to -80 ºC prior analysis (under one month). No further description or characterization.	Cells lysed, then RNA isolated using a QIAprep Spin Miniprep Kit.<br>DNase step NR	Yes (quantity by NanoDrop)<br>(values NR)	*18S rRNA*
Knobloch TJ et al., 2016 [48]	Oral cancer and distal normal high risk oral mucosa	Incisional biopsies obtained during surgical resection, collected into Ambio RNAlater reagent.	Total RNA isolated using Rneasy Fibrous Tissue Kit.<br>Dnase step included	Yes (quantity by NanoDrop and quality by Bionalayzer)<br>(RIN values=6-9)	*DUSP1*
Gasper AV et al., 2007 [12]	Gastric antrum	Gastric antral biopsies (fasted endoscopy) obtained 1 d before and 6 h after treatment, kept into RNAlater, held at 4 ºC overnight, then snap frozen in liquid N_2 and stored at -80 ºC prior to RNA extraction. No further information or characterization of the samples.	Total RNA isolated using RNeasy mini kit.<br>DNase step NR.	Yes (quantity by NanoDrop, quality by Bioanalyzer)<br>(values NR)	*18S rRNA*
Koosirat C et al., 2010 [82]	Gastric antrum	Biopsies obtained at baseline and at the end of the study (42 d), kept in RNAstabilization solution at 4 ºC. Biopsies homogenized using QIAshredder. No details of biopsies extraction or tissue characterization.	RNA isolated using RNeasy mini spin column.<br>DNase step NR.	NR	*GAPDH*
Labonté M-E et al., 2013 [61]	Duodenal tissue	Gastro-duodenoscopy from the second portion of duodenum with multiple sample single-use biopsy forceps. Flash frozen in liquid N_2. Stored at -80 ºC.	Biopsy samples homogenized using Qiazol, then RNA isolated using RNeasy mini kit. RNA stored at -80 ºC.<br>DNase step included.	Yes (quality assessed by Bioanalyzer)<br>(values NR)	*ATP5O* (selected from *HPRT1, ATP5O, G6PD, 18SrRNA* based on the literature)
Frommel TO et al., 1994 [66]	Colon tissue	Colon tissue samples obtained at the baseline and at the end of study, using biopsy forceps, placed in RPMI medium at 4 ºC and transported to the lab microfuge for RNA extraction.	Total RNA isolated using the method of Chomczynski and Sacchi [86].<br>DNase step NR.	Yes (quantity and purity based on Abs $_{260/280}$ nm)<br>(values NR)	*B2M*
Nguyen AV et al., 2009 [42]	Colon tissue (cancer, normal)	Tissue cancer and normal biopsies obtained at colonoscopy (pre-treatment). Samples taken for pathologist revision and placed in RNAlater. Biopsies taken at surgical resection (post-treatment). No further details of the tissue characterization.	Total RNA isolated using a Trizol reagent.<br>DNase step NR.	NR	*ACTB*

Ishikawa H et al., 2012 [41]	Colon mucosa	Biopsies from normal tissue taken at baseline (afternoon) and after intervention (morning) by endoscopy (20 cm from the anal verge). Samples were immersed in RNAlater and kept in ice in the dark prior to analysis.	RNA isolation not described.<br>DNase step NR.	NR	*HMBS*
Nuñez-Sanchez MA et al., 2017 [50]	Colon tissue (cancer, normal)	Biopsies taken at colonoscopy (pre-treatment) and surgical samples (post-treatment) from normal and malignant tissue. Samples taken to the pathologist for analysis and into RNAlater. Stored at -80 ºC.<br>All colon tissue samples homogenized with lysis buffer using an IKA T10 Ultra-Turrax Equipment.	Total RNA isolation not described. Based on previous work.<br>DNase step NR.	Yes (quantity and quality by Nanodrop and Bioanalyzer) (Abs $_{260/280}$ 1.8-2.1, RIN values> 6)	*GAPDH* (stability tested by Normfinder/ GeNorm for *HPRT1, GUSB, GAPDH)*
**Other tissue samples**					
González-Sarrías A et al., 2010 [30]	Prostate tissue (benign hyperplasia and cancer tissues)	Surgical prostate tissue biopsies obtained either by prostatectomy, transurethral resection or adenomectomy, examined by pathologists, then stored in RNAlater at -80 ºC, prior to RNA extraction.	RNA extracted using RNeasy mini kit.<br>DNase step NR.	Yes (quantity and quality by NanoDrop and 1% agarose gel) (Abs $_{260/280}$ 1.8-2.1)	*GAPDH*
Chan JM et al., 2011 [21]	Prostate tissue (normal)	Ultrasound-guided four-core needle research biopsies were taken at baseline and after intervention. Tissues were reviewed by the pathologist. No further description.	Total RNA extracted from areas of normal prostate peripheral zone tissue containing both stroma and epithelial cells. Method NR.<br>DNase step NR.	Yes (quality and quantity adequate for the samples analyzed, values NR)	*GUSB*
Lazarevic B et al., 2012 [79]	Prostate tissue (cancer, normal)	Surgical specimen (post-treatment) from normal and cancer tissues, examined by the pathologist and frozen at -80 ºC. At least 10,000 cells from histologically benign and cancer glands from each patient selected and captured using laser capture microdissection, stored at -80 ºC, prior to RNA extraction.	Total RNA isolated using the Arcturus PicoPure RNA Isolation Kit.<br>DNase step NR.	Yes (quantity by NanoDrop) (values NR)	*ALAS1*
Most J et al., 2015 [81]	Adipose tissue	At the end of post-prandial period (6 h), 1 g abdominal subcutaneous adipose tissue collected under local anaesthesia using a needle biopsy technique. Snap frozen in liquid N_2. Stored at -80 ºC	Total RNA extracted using total RNA stabilization and purification kit for human samples<br>DNase step NR.	NR	*18S rRNA*
Kruse M et al., 2015 [29]	Adipose tissue	Periumbilical adipose tissue (1 g) taken at baseline and at the end of acute study (4 h) and chronic study (28 d), rinsed and snap frozen in liquid N_2. Stored at -80 ºC. No other information described.	RNA isolation not described (based on previous work).<br>DNase step NR.	NR	*RPL32*
Poulsen MM et al., 2013 [70]	Skeletal muscle and adipose tissue	Tissue biopsies obtained at the end of the basal period and 20 min into the clamp period , (both at the baseline and at the end of the	Total RNA isolated from muscle and adipose tissue using Trizol .<br>DNase step NR.	Yes (quantity by NanoDrop; quality by agarose gel)	*B2M*

		study) under sterile conditions and anesthesia (lidocaine). The muscle biopsy was taken from the *vastus lateralis* muscle (Bergström needle). Tissue dissected from fat and connective tissue and frozen in liquid N_2. Periumbilical subcutaneous abdominal fat was taken by liposuction 15 cm lateral to the umbilicus, cleaned and frozen in liquid N_2. No further description of the tissues.		(Abs $_{260/280}$ ≥1.8)	
Yoshino J et al., 2012 [69]	Skeletal muscle and adipose tissue	Anesthetized (lidocaine). A ~0.5 cm skin incision made with a scalpel; subcutaneous abdominal adipose tissue aspirated (liposuction) and muscle skeletal (*Vastus lateralis*) tissue obtained by Tilley-Henkel forceps during the basal period of the clamp. Samples were rinsed, frozen in liquid N_2 and stored at -80 ºC.	Total RNA isolation protocol not described. DNase step NR.	NR	*GAPDH*
Kerksick CM et al., 2013 [80]	Skeletal muscle	At the end of intervention, muscle samples were taken immediately prior to and 6 and 24 h post-exercise under anesthesia (lidocaine), using a 16-gauge microbiopsy needle, from the middle portion of the *vastus lateralis* at a depth of 4-5 cm. Tissue trimmed of adipose contamination, frozen in liquid N_2 and stored at -80 ºC.	Tissue samples homogenized and RNA isolated using TRI reagent phase method. Samples were washed, diluted in RNAase-free water, then stored at -80 ºC until analyses.<br>DNase step NR.	Yes (quantity and quality by spectrophotometer and optical density at 260 nm)<br>(values NR)	*ACTB*
Olesen J et al., 2014 [71]	Skeletal muscle	On the second day (48 h after the first experimental day), and at the end of intervention, samples of the *vastus lateralis* muscle were obtained under local anaesthesia (lidocaine) using the percutaneous needle biopsy technique with suction. Samples were then quick-frozen in liquid N_2 and stored at -80 ºC until analysis.	Total RNA isolated from muscle tissue by a modified guanidinium thiocyanate–phenol–chloroform extraction method by Chomczynski and Sacchi [86], modified in the step of the tissue homogenization.<br>DNase step NR.	Yes (quantity and quality by spectrophotometer)<br>(Abs $_{260/280}$ >1.8)	*ssDNA*
Riedl MA et al., 2009 [14]	Cells from nasal lavage	Nasal lavage collection at baseline and at the end of study, procedure referred in the Supplementary material. Cells were collected using sterile saline lavage. The cells were then pelleted and re-suspended in lysis buffer and stored at -80 ºC. No information on cell composition or characterization.	RNA extracted using RNeasy Mini Kit and stored at -80 ºC until RT-PCR analysis.<br>DNase step NR.	Yes (quantity measured but method not indicated)<br>(RNA yield: 0.3-1.0 µg per sample)	*18S rRNA*
Knott A et al., 2008 [51]	Suction blister epidermis	After 12 weeks of treatment, suction blister epidermis isolated (protocol referenced). No further details of the sample characteristics.	Suction blister epidermis samples homogenized in RNAzol and Ultra Turrax T8 for RNA extraction.<br>DNase step NR	NR	*18S rRNA*

Marini A et al., 2014 [63]	Skin	4 mm punch biopsies taken before and after supplementation from unexposed and exposed skin areas. No other detail included. Based on previous references.	RNA extraction based on previous work. DNase step NR.	NR	NR
Marini A et al., 2012 [54]	Buttock skin	4 mm punch biopsies from buttock skin taken before and after supplementation. Samples snap-frozen in liquid N_2 and stored at -80 ºC prior to RNA isolation.	RNA extracted from frozen samples using Peq-Gold Total RNA Kit. DNase step NR.	NR	*18S rRNA*
Farris P et al., 2014 [64]	Photo-damaged face skin	2 mm punch biopsies collected on the facial area at the baseline and at the end of the study. Quick frozen in liquid N_2. Stored at -80 ºC.	RNA isolated using RNA micro kits. DNase step NR.	NR	NR
Bertuccelli G. et al., 2016 [40]	Forearm skin	3-ml punch biopsies obtained from the extensor side of forearms using Corneofix® foils, at the beginning and at the end of the study.	Cells lysed using Ambion lysis buffer. Lysates mixed with ethanol and centrifuged, washed, eluted using buffers. RNA retrieved by centrifugation. DNase step included.	Yes (quantity by optical density at 260 nm; quality in 1.5% agarose gel) (values NR)	*ACTB* or *GAPDH* (unclear)

[1] **W**hite blood cells or leukocytes**:** mononuclear cells agranulocytes (lymphocytes and monocytes) and polymorphonuclear granulocytes (neutrophils, eosinophils, basophils, mast cells)**;** lymphocytes (mostly T cells, B cells and NK cells, also some dendritic cells); leukemic blasts (myeloblasts or immature white blood-forming cells)**.**

Table abbreviations (in alphabetical order): DEPC, diethyl pyrocarbonate; DNA, deoxyribonucleic acid; DNase, *deoxyribonuclease;* EDTA, ethylenediaminetetraacetic acid; miRNA, microRNA; MNC, mononuclear cells; NR, not reported; PBMC, peripheral blood mononuclear cells; RIN, RNA integrity number; RNA, ribonucleic acid; *RPMI,* Roswell park memorial institute medium; rRNA, ribosomal RNA; RT-PCR, real time polymerase chain reaction; ssDNA, *single-stranded DNA; WBC, white blood cells*.

Genes nomenclature from GeneCards [89] (in alphabetical order): *ACTB,* actin beta; *ALAS1*, 5'-aminolevulinate synthase 1; *ATP5O,* ATP synthase, H+ transporting, mitochondrial F1 complex, O subunit; *AW109,* competitor RNA (cRNA); *B2M*, beta-2-microglobulin; *DUSP1,* dual specificity phosphatase 1; *GAPDH*, glyceraldehyde-3-phosphate dehydrogenase; *G6PD*, glucose-6-phosphate dehydrogenase; *GUSB*, glucuronidase beta; *HMBS*, hydroxymethylbilane synthase (alias: *PBGD,* porphobilinogen deaminase); *HPRT1*, hypoxanthine phosphoribosyltransferase 1; *PPIA*, peptidylprolyl isomerase A; *RPLP0,* ribosomal protein lateral stalk subunit P0; *RPL13A*, ribosomal protein L13a; *RPL32,* ribosomal protein L32; *18S rRNA*, 18S ribosomal RNA; *UBC*, ubiquitin C; *YWHAZ*, tyrosine 3-monooxygenase/tryptophan 5-monooxygenase activation protein zeta.

Table S3. Reported changes in gene expression levels in human tissues (as analyzed by RT-PCR) following intervention with diets, foods or products rich in bioactive compounds or with single compounds (↓green color, significant downregulation; ↑red color, significant upregulation, attributed to treatment either in comparison with baseline or with control; *p*-values are indicated, $p<0.05$ are accepted as significant). The type of comparisons performed in the study, the way data are presented and whether there were any association(s) with bioactive metabolites and (or) with protein quantity/activity are also included.

**Gene Expression Results**										
Reference	Treatment description (T) (major bioactive compound(s))	Type of sample	Gene(s)<br>Change associated with T (↓, ↑) (*p*-value)	Comparisons (number of samples)		Data presentation			Association with	
				Post- *vs* pre-	Post-	Type of data: expression levels, change (ratio, FC, %)	Type of values: mean, median, individual data	Variability information: SD, SEM, range, IQR, 95% CI, % of individuals with a change	Metabolites	Protein levels, activity
**Blood**										
Almendingen K et al., 2005 [3]	Mix FruVeg<br>T_1: 300g<br>T_2: 750g	Blood	(NC) *PTGS2*	T_1 (39)<br>T_2 (39)<br>(different doses)	T_2 (39) *vs* T_1 (39)	Expression levels	Mean	SEM	NR	NR
Guarrera S et al., 2007 [35]	Green tea, bilberry juice and soya (flavonoids)	Blood	↓***AHR*** (*p*=0.038)<br>↑***XRCC3*** (*p*=0.038)<br>(NC) *APEX1, ERCC1, ERCC2, ERCC4, MGMT, OGG1, XPA, XPC,XRCC1, CYP1A1*	T (9)	NR	Change (FC)	Mean	SD	NR	NR
Doss JF et al., 2016 [18]	Broccoli (SFGluc)<br>T_1: 50g<br>T_2: 100g<br>T_3: 150g	Blood	↑***HBG1*** (+130.8%, *p*=0.025, T_2)<br>↑***HMOX1*** (+53.5%, *p*=0.045, T_3)<br>(NS↑) *NQO1* (T_1, T_2)	T_1 (5)<br>T_2 (5)<br>T_3 (4)<br>(different doses)	NR	Relative mRNA expression, change (%) in text	Mean (values only in figure)	Unclear (values only in figure)	NR	(NC) Hbg or HbF (some individual values in Figure)
Atwell LL et al., 2015 [17]	T_1: Broccoli sprout (SFGluc)<br>T_2: Myrosinase-treated broccoli sprout extract (SFGluc)	Blood	(NC) *CDKN1A, HMOX1*	T_1 (10)<br>T_2 (10)<br>(myrosinase increases the levels of SF *vs* untreated)	T_1 (10) *vs* T_2 (10)	Change (FC)	Mean and geometric mean (values only in figure)	SEM, SD (values only in figure)	NR	(NC) plasma levels of HMOX1 (HO-1)
Di Renzo L et al., 2014 [8]	T_1: Red wine<br>T_2: Med meal<br>T_3: T_1+T_2<br>T_4: McD meal<br>T_5: T_4+T_1	Blood	↓***CAT*** (*p*≤0.05, T_4)<br>↑***CAT*** (*p*≤0.05, C *vs* T_1, T_4 *vs* T_5)<br>↑***GPX1*** (*p*≤0.05, C *vs* T_1, C *vs* T_3, C *vs* T_5)<br>↑***SIRT2*** (*p*≤0.05, T_1 *vs* T_3)<br>↑***CCL5*** (*p*≤0.05, C *vs* T_2, C *vs* T_3, C *vs* T_4, C *vs* T_5, T_1 *vs* T_5)<br>↓***CCL5*** (*p*≤0.05, T_2 vs T_3)<br>(NC) *SOD1*	C (24), T_1 (24), T_2 (24), T_3 (24), T_4 (24), T_5 (24),	C (24) *vs* T_1 (24),<br>C (24) *vs* T_2 (24),<br>C (24) *vs* T_3 (24),<br>C (24) *vs* T_4 (24),<br>C (24) *vs* T_5 (24),<br>T_1 (24) *vs* T_5 (24),<br>T_2 (24) *vs* T_3 (24)	Change (unclear, ΔCt in figure, FC in methods)	Unclear (values only in figure)	95% CI (values only in figure)	NR	NR

Weseler AR et al., 2011 [43]	Grape seed extract (Flavanols)	Blood	↓*IL-6* (-18%, $p<0.05$, T, 28 d)<br>↓*TNF*, ↓*IL10* (-12%, -27%, $p<0.05$, T, 56 d)<br>(NS↓) *CAT, GSR, HMOX1* (T, 56 d)<br>(NC) *IL1β, CXCL8, NOS2, NFKBIA, ICAM1, VCAM1, GPX1, GPX4, SOD2*	T (15) , C (13) (2 time points)	T(15) *vs* C (13)	% Change (in text)	Unclear (values only in figures)	Unclear (values only in figure)	NR	Plasma:↓TNF T (post- *vs* pre-)<br>T *vs* C (post-)<br>(NC) IL10
De Lorenzo A et al., 2016 [9]	T_1: Tocopherol-enriched Med meal<br>T_2: Western high -fat meal	Blood	↑ ***IRAK1, CCL5, DUOX2, UCP2*** ($p<0.05$, post- *vs* pre-, T_2)<br>↓***BCL2***, ↑***CCL5*** ($p<0.05$, post- *vs* pre-, T_1)<br>↓ ***BCL2, IRAK1, DUOX2, UCP2*** ($p<0.05$, T_2 *vs* T_1)	T_1 (25), T_2 (25)	T_1 (25) *vs* T_2 (25)	Change (FC)	Unclear (values only in figure)	Unclear (values only in figure)	NR	NR
Vors C et al., 2017 [67]	T_1: EPA<br>T_2: DHA	Blood	↓ ***CD14*** (p=0.008, T_1 *vs* C, p=0.02 T_2 *vs* C)<br>↑ ***PPARA*** (p=0.003 T_1 *vs* C, p=0.01, T_2 *vs* C)<br>↑ ***TNF*** (p=0.06, T_1 *vs* C, p=0.01 T_2 *vs* C)<br>↑ ***TRAF3*** (p=0.002, T_1 *vs* C p=0.07, T_2 *vs* C)<br>(NC) *CCL2, IL10, IL1B, IL1RN, NFKB1, PPARG, TNFRSF1A*	NR	T_1 (44) *vs* C (44)<br>T_2 (44) *vs* C (44)<br>T_1 (44) *vs* T_2 (44)	Nº copies mRNA	Mean	SEM	↑% of EPA and DHA in plasma phospholipids, but no relation with gene expression	No correlation between *TNF* and plasma levels of TNF
Donadio JLS et al., 2017 [32]	Brazil nuts (Se)	Blood	↑ ***GPX1*** (p=0.026, T, just for the CC genotype)<br>(NC) ***SELENOP, SELENOS, SELENOF***	T (130) (different genotypes)	NR	Relative expression levels	Unclear (values only in figure)	Unclear (values only in figure)	NR	NR<br>*Different responses to supplementation according to genotype
Di Renzo L et el., 2017 [34]	T_1: McD meal+ raw hazelnuts<br>T_2: McD meal	Blood	53 genes up- or downregulated ($p>0.05$, FC>1.5 or<-1.5, T_2 *vs* T_1)<br>Examples: ↑***TNF, TNFSF4, GPX1, GPX3, GPX4***<br>↓ ***NQO1, GPX7, UCP2***	T_1 (22),<br>T_2 (22),	T_1 (22) *vs* T_2 (22),	Change (FC)	Unclear (values only in figure)	NR	NR	NR
**Peripheral blood isolated immune cells[1]**										
Møller P et al., 2003 [2]	Mix FruVeg	White blood cells	(NC) *OGG1, ERCC1*	T (11-15),<br>C_1 (9-15),<br>C_2 (9-12)<br>(variable number of samples depending on RNA quality)<br>(5 time points)	T (11-15) *vs* C_1 (9-15) *vs* C_2 (9-12)	Expression levels	Mean	SD	NR	OGG1 responsible for the excision of 8-oxoguanine.<br>No association with urinary

										8-oxodG (mutagenic byproduct of exposure to reactive oxygen).
Dragsted LO et al., 2006 [6]	Mix FruVeg (C_1, C_2, different controls)	White blood cells	(NS↑) *GPX1* in T (post- *vs* pre-, in 2 subjects) , (NC) *GCLC, FOSL1, AHRR*	T, C_1, C_2 (n=43 divided in the three groups, unclear)	T *vs* C_1 T *vs* C_2 , C_1 *vs* C_2 (post-)	Change (FC)	Mean (values only in figures)	SEM (values only in figures)	NR	Erythrocytes: ↑GPX1 activity in T (post- *vs* pre) [7]
Farràs M et al, 2013 [23]	Olive oil (polyphenols) (T_1: high T_2: moderate levels)	White blood cells	↑*ABCA1, SCARB1, PPARG, PPARA, PPARD, MED1, CD36* (*p*=0.017 , T_1 *vs* T_2) ↑*PTGS1* (*p*= 0.024, T_1 *vs* T_2) (NC) *ABCG1, PTGS2*	NR	T_1 (13) *vs* T_2 (13) (different doses)	Change (ratio)	Geometric mean (values only in figure)	95 % CI (values only in figure)	↑ of 1 µmol/L of HTyr acetate and ↑43.2% of *ABCA1* in T_1	NR
Daak AA et al., 2015 [60]	n-3 PUFA (EPA, DHA) (C_1, C_2, different controls)	White blood cells	↓*NFKB1* (*p*<0.05, T *vs* C_1) (NS↓, T *vs* C2)	NR	T (24) *vs* C_1 (21), T (24) *vs* C_2 (18), C_1 (21) *vs* C_2 (18)	Change (FC)	Unclear (values only in figure)	Unclear (values only in figure)	NR	NR
Marques-Rocha JL et al., 2016 [10]	Med diet	White blood cells	(NC) *IL6, TNF, ICAM1, IL18, SERPINE1, VCAM1*	T (40)	NR	Change (FC)	Mean	SD	NR	NR
Mansur AP et al., 2017 [74]	Res	White blood cells	(NC) *SIRT1*	T (24), C (24)	T (24), C (24)	Arbitrary units	Mean	SD	NR	↑ Serum hSIRT1 in both T (post- *vs* pre-) and C (post- *vs* pre-)
Plat J and Mensik RP, 2001 [57]	Plant stanol esters mix	Mononuclear cells	↑*LDLR* (+43%, *p*=0.003) T_{1+2} *vs* C (NC) *HMGCR*	T_{1+2} (29), C (15)	T_{1+2} (29) *vs* C (15)	Change (%), Nº copies/µg RNA	Mean	95% CI, SEM	Negative correlation with changes in Cam, Positive correlation with changes in Lath, Negative correlation with LDL cholesterol	↑LDL receptor protein in monocytes (+37%, *p*=0.003) and in T-lymphocytes (+25%, *p*=0.013) in T_{1+2} *vs* C.
Konstantinidou V et al., 2010 [20]	Med diet+ olive oil (polyphenols)	Mononuclear cells	↓*ADRB2, ARHGAP15, IL7R, POLK, IFNG* (*p*<0.05, T_1+T_2 *vs* C)	T_1+T_2 (36), C (20), T_1(20), T_2 (16)	T_1+T_2 (36) *vs* C(20) T_1 (20) *vs* T_2 (16) , T_1 (20) *vs* C (20),	Change (ratio)	Mean	SEM	↓*IFNG* with ↑Tyr in T_1	↓ IFNγ in plasma (T_1+T_2 post-

	(T_1: high, T_2: low polyphenols)		↓*ADRB2, ARHGAP15, IFNG* (*p*<0.05, T_1 *vs* C)<br>(NS↓) *IL7R* (*p* = 0.052, T_1 *vs* C, post-)		T_2 (16) *vs* C (20)					*vs* pre-; T_1, post- *vs* pre-)<br>NC *vs* C
Riso P et al., 2010 [16]	Broccoli (SFGluc, lutein, *β*-carotene, vitC)	Mononuclear cells	(NC) *OGG1, NUDT1, HMOX1*	C (17), T (17)	NR	Expression levels	Mean	SD	NR	NR
Shrestha S et al., 2007 [52]	Psyllium + plant sterols	Mononuclear cells	↑*LDLR* (+26%, *p*<0.03)<br>(NC) *HMGCR*	NR	T (17) *vs* C (17)	Expression levels, change (%)	Mean (individual data in figure)	SD (individual data in figure)	NR	↓ plasma cholesterol concentration in LDL subfractions in T *vs* C (post-)
Boss A et al., 2016 [59]	Olive leaf extract (oleuropein, HTyr)	Mononuclear cells	↓*EGR1* (*p*=0.025)<br>↓*PTGS2* (*p*=0.016)<br>↑*ID3* (*p*=0.023)	NR	T (15) *vs* C (14)	Change (FC)	Mean	SD	NR	NR
Klickovic U et al., 2014 [83]	Cur	Mononuclear cells	(NC) *HMOX1*	T (10)	NR	Baseline expression levels (ΔCt) in table and change (FC) in figure	Mean	SD (stratification of results by genotype)	No Cur detected in plasma at any time point (48 h)	(NC) HMOX1 in PBMC
Hernáez Á et al., 2015 [26]	Olive oil (polyphenols) (T_1: high T_2: low content)	Mononuclear cells	(NS↑) *LPL* (*p*=0.08, T_1)	T_1 (18), T_2 (18) (different doses)	T_1 (18) *vs* T_2 (18)	Change (%)	Mean (values only in figure)	SEM (values only in figure)	NR	NR
Barona J et al., 2012 [44]	Grape powder	Mononuclear cells	↑*NOS2* (*p*<0.001, individuals without dyslipidemia, T *vs* C<br>(NC) *CYBB, SOD1, SOD2, GPX1, GPX4*	T(24), C(24)	T (24) *vs* C (24) (dyslipidemia 11), (non-dyslipidemia, 13)	Arbitrary units	Mean	SD (stratification of results by dyslipidemia status)	NR	NR
Ghanim H et al., 2010 [53]	*Polygonum cuspidatum* extract (Res)	Mononuclear cells	↓ *MAPK8, IKBKB, PTPN1, SOCS3*<br>↑*IRS1* (all comparisons *p*<0.05, T post- *vs* pre-, T *vs* C)<br>(NC) *TLR4, SIRT1*	T (10) , C (10) (several time points)	T (10) *vs* C (10)	Change (%)	Mean	SEM	NR	↓MAPK8, ↓PTPN1 in MNC, T (post- *vs* pre-), T *vs* C (post-)
Martín-Peláez S et al ., 2015 [28]	Olive oil (polyphenols) (T_1: high T_2: low content)	Mononuclear cells	↓*ACE* (*p*=0.014 T_1)<br>↓*NR1H2* (*p*=0.022, T_1),<br>↓*CXCR2* (, *p*=0.02, T_1 *vs* T_2)<br>(NS↓) *CXCR1, ADRB2* (T_1)<br>(NS↓) *MPO* (T_1, T_2)<br>(NS↓) *ADRB2, ACE* (T_1 *vs* T_2)	T_1 (18) , T_2 (18) (different doses)	T_1 (18) *vs* T_2 (18)	Change (log_2 ratio)	Mean (values only in figure)	SD (values only in figure)	NR	NR

			(NC) *ECE2, OLR1*							
Camargo A et al., 2010 [19]	Olive oil (polyphenols) (T_1: high T_2: low content)	Mononuclear cells	↓***EGR1*** (*p*=0.014) ↓***IL1B*** (*p*=0.006) (NS↓) *JUN, PTGS2* (*p*=0.083, *p*=0.118)	NR	T_1 (20) *vs* T_2 (20) (different doses)	Expression levels	Mean (values only in figure)	SEM (values only in figure)	NR	NR
Rangel-Zuñiga OA et al., 2014 [25]	T_1: Virgin olive oil T_2: Sunflower oil T_3: Sunflower + canola oil+ dimethylpolysiloxane T_4: olive antioxidants	Mononuclear cells	↑***XBP1***(*p*=0.04, 4h *vs* pre-, T_2) ↑***HSPA5*** (*p*=0.035, 4h *vs* pre-, T_2) ↑***CALR*** (*p*=0.023, 4h *vs* pre-, T_2)	T_1 (20), T_2 (20), T_3 (20), T_4 (20) (0, 2h, 4h)	T_1 (20) *vs* T_2 (20) *vs* T_3 (20) *vs* T_4 (20)	Expression levels	Mean (values only in figure)	SEM (values only in figure)	NR	NR
Castañer O et al., 2012 [22]	Olive oil (polyphenols) (T_1: high T_2: low polyphenol content)	Mononuclear cells	↓***CD40LG, IL23A, IL7R, CXCR2, OLR1*** (*p*<0.05, T_1, T_1 *vs* T_2) ↓***ADRB2*** (*p*< 0.05, T_1 *vs* T_2) ↓***CCL2*** (*p*< 0.05, T_1, T_2) (NS↓) *IFNG, VEGFB, ICAM1* (T_1) (NC) *ALOX5AP, TNFSF10*	T_1 (18), T_2 (18) (different doses)	T_1 (18) *vs* T_2 (18)	Change (%)	Mean (values only in figure)	SEM (values only in figure)	Inverse correlation between ↓*OLR1* (-2.6) and ↓*ICAM1* (-2.8) and the 10% ↑Tyr and ↑HTyr in urine	↓CCL2 T_1 *vs* T_2 (NC) ICAM1, IFNG
Crespo MC et al., 2015 [58]	Olive waste water extract (enriched in HTyr)	Mononuclear cells	(NC) Phase II enzymes: *NQO1,2; GSTA1,4; GSTK1, GSTM1-5; GSTO1,2; GSTP1; GSTM1,2; HNMT; INMT; MGST1-3*	T_1 (21), T_2 (21), C (21) (different doses)	T_1 (21) *vs* T_2 (21) *vs* C (21)	Change (FC)	Mean	SEM	NR	NR
Kropat C et al., 2013 [47]	Bilberry pomace extract (anthocyanins)	Mononuclear cells	↑***NQO1*** (*p*<0.05 at 1h, 2h, 4h, *p*<0.01 at 8h, healthy) ↓***HMOX1*** (*p*<0.001, at 1h, 2h, 4h, *p*<0.05 at 8h, healthy) ↓***NFE2L2*** (*p*<0.001, at 2h, 4h, *p*<0.01 at 8h, healthy; *p*<0.001 at 2h, *p*<0.05 at 4h, 8h ileostomy)	T (5 healthy, 5 ileostomy) (different time points: pre-, and 1h, 2h, 4h, 8h post-)	NR	Expression levels (%)	Mean	SD	NR	NR
Perez-Herrera A et al., 2013 [24]	T_1: Virgin olive oil T_2: Sunflower oil T_3: Sunflower + canola oil+ dimethylpolysiloxane T_4: olive antioxidants	Mononuclear cells	NADPH-oxidase subunits: ↑***CYBB*** (*p*=0.013, 4h, T_2, *p*= 0.030, 4h, T_2 *vs* T_1) , ↑ ***NCF1*** (*p*=0.021, 2h, *p*=0.033, 4h T_2) ↑ ***CYBA*** (*p*=0.026, 4h, T_2, *p*=0.009, 4h, T_3) ↑ ***NFE2L2*** (*p*=0.015, 2h, *p*<0.05, 4h, T_2) ↑***SOD1*** (*p*=0.040, 2h, *p*<0.05 4h, T_2)	T_1 (12), T_2 (12), T_3 (12), T_4 (12)	T_1 (12) *vs* T_2 (12), T_2 (12) *vs* T_3 (12), T_2 (12) *vs* T_4 (12)	Expression levels	Mean (values only in figure; difficult to discern significant comparisons between treatments and between pre- and post-samples)	SEM (values only in figure)	NR	In PBMC: (NC) NFE2L2 cytoplasmic levels with T, Nuclear NFE2L2 levels ↓ upon T_1, T_3, T_4 (*p*=0.016, 0.027, 0.023) but NC with

			↑*CAT* (p= 0.034, 4 h, T_2 *vs* T_1 p= 0.015, 4h, T_2 *vs* T_3, p<0.05, 4h, T_2) ↑*GSR* (p=0.026, 2h, T_2, p<0.05, 4h, T_2) ↑*GSTP1, TXN* (p<0.05, 4h, T_2)							T_2 SOD activity ↓ after T_1 (p=0.041)
Chachay VS et al., 2014 [72]	Res	Mononuclear cells	(NC) *NQO1, PTP1B, IL6, HMOX1*	T (9) (different time points)	NR	Expression levels	Median (values only in figure for *PTP1B* and *NQO1*). Table with *p*-values	Inter-quartiles ranges (values only in figure)	NR	↓plasma IL6 in T (post- *vs* pre-)
Yiu EM et al., 2015 [73]	Res (T_1, T_2, two doses)	Mononuclear cells	(NC) *FXN* (T_1) (NS↓) *FXN* (p=0.08, T_2)	T_1 (12) , T_2 (12)	T_1 (12) vs T_2 (12)	Change (%)	Mean	SD, 95% CI	NR	(NC) FXN levels in PBMC
Xie, L et al., 2017 [49]	Aronia berry extract (polyphenols)	Mononuclear cells	(NC) *LDLR, HMGR*	T (25), C (24)	T (25) *vs* C (24)	Expression levels	Mean	SEM	No association between plasma/urine aronia metabolites and gene expression	↓LDL receptor in PBMC by 56% in the T (post- *vs* pre), (p= 0.0036) T *vs* C (post-)
Radler U et al., 2011 [65]	Low-fat yoghurt containing grapeseed extract + fish oil + phospholipids +L- Carn + VitC + VitE	Mononuclear cells	↑***PPARG, CPT1A, CPT1B, CRAT, SLC22A5*** (p<0.05, T)	T (22) C (20; results NC but data not included)	NR	Change (FC)	Mean	SD (values only in figure)	↑*CRAT* and ↑urine acetyl-Carn in T	NR
Tomé-Carneiro J et al., 2013 [45]	Grape extract + Res (T_1: polyphenols T_2: polyphenols +Res)	Mononuclear cells	↓ ***IL1β*** (T_2, p<0.001; T_1, p<0.01; T_1 or T_2 *vs* C, p<0.05) ↓ ***TNF*** (T_2, p<0.01; T_2 *vs* C, p=0.059) ↓ ***CCL3*** (T_2, p<0.01) ↑ ***LRRFIP1*** (T_2, p<0.01) ↓ ***NFKBIA*** (T_1, p<0.01) (NC) *NFKB1*	T_1 (13), T_2 (13), C (9) (different time points)	T_1 (13) *vs* C (9), T_2 (13) *vs* C (9), T_2 (13) *vs* T1 (13)	Change (ratio)	Median	25th-75th interquartile range	NR	NC in TNF levels in PBMC or serum
Jamilian M et al., 2018 [62]	Fish oil (EPA + DHA)	Mononuclear cells	↑***PPARG*** (p= 0.04, T *vs* C) ↓***LDLR*** (p< 0.001, T *vs* C) ↓***IL1B*** (p = 0.007, T *vs* C) ↓***TNF*** (p = 0.01, T *vs* C) (NC) *CXCL8*	NR	T (20) *vs* C (20)	Change (FC)	Mean (values only in figure)	SD (values only in figure)	NR	NR
Nelson DM et al., 2011 [78]	Flavopiridol	Leukemic blasts	↑***BCL2*** (p=0.0005) (NS↑) *CCDN1* (p=0.104) ↓***HMGA1*** (p=0.0005)	T (16)	NR	Expression levels in text , change (%) in	Mean (data stratification by responders	SD, range, % of individuals exhibiting the	NR	NR

			↓***STAT3*** (*p*=0.041)<br>↓***E2F1*** (*p*=0.009)<br>↓***POLR2A***(*p*=0.034)<br>(NC) *MCL-1, VEGFA*			figure	and non-responders)	change		
Persson I et al., 2000 [1]	Mix Veg	Lymphocytes	↓ ***GSTP1*** (*p* < 0.05)	T (6)	NR	Change (ratio) % change	Median	Range (%), individual values	NR	↓ GSTP1 in lymphocytes
Morrow DMP et al., 2001 [75]	Quer	Lymphocytes	↓ ***TIMP1*** (-75 and -85%, T *vs* C, T post- *vs* pre-, T *vs* washout, in the 4 individuals)<br>(NC) *TIMP2, MMP2*	T (4), C (4)	T (4) *vs* C (4)	Change (%)	Individual values (only in figure)	NR	NR	↓ TIMP-1 in plasma in T *vs* C, T *vs* baseline, T *vs* after 35 days of the end of T
de Pascual-Teresa S et al., 2003 [38]	Quer (onions)<br>T_1: low Quer<br>T_2: high Quer	Lymphocytes	(NC) *PTGS2*	T_1 (8), T_2 (8) (different doses)<br>C (8)	NR	Expression levels	NR	NR	No association with plasma Quer metabolites	NR
Boettler U et al., 2012 [37]	Coffee (CGA, NMP)	Lymphocytes	↑***NFE2L2*** (*p*<0.05, T post- *vs* pre-, post- *vs* after study washout)<br>(NS↑) *NFE2L2* in T (post- *vs* before baseline)	T (18) (different time points)	NR	Change (FC)	Median (most values only in figure)<br>Mean (values in figure and table)	Box plots with quartiles (most values only in figure; stratification in responders and not responders and effect of genotype)	NR	NR
Volz N et al., 2012 [36]	Coffee (CGA, NMP)	Lymphocytes	↑***NFE2L2*** (*p*< 0.05, T, post- *vs* study washout)<br>↓***HMOX1, SOD1*** (*p*< 0.05, T, post- *vs* study washout)<br>(NC) *GCLC, NQO1, GSTT1, CAT, GPX1, GSTM5, GSR*	T (22-29)	NR	Change (FC)	Mean (most values only in figure)	Box plots with quartiles (most values only in figure) (stratification in responders and not responders and effect of genotype)	NR	NR
Hernández-Alonso P et al., 2014 [31]	Pistachio	Lymphocytes	↓***IL6, RETN, SLC2A4***<br>(-9%, *p*= 0.004, -6%, *p*= 0.04, (+69%, *p* =0.03, T *vs* C)<br>(NC) *SLC2A3, TLR2, TLR4*	T (49), C (49)	T (49) *vs* C (49)	Change (%) in text for T *vs* C, change (ratio) T, C (post- *vs* pre-)	Unclear (values only in figure)	Unclear (values only in figure)	NR	(NC) plasma IL-6 and resistin T, C (post- *vs* pre-), T *vs* C (post-)
Carrera-Quintanar L et	T_1: *Lippia citriodora* extract	Lymphocytes neutrophils	Neutrophils<br>↓***SOD2, SOD1, GSR*** (*p*<0.05, T_3),	C (8), T_1 (8), T_2 (9), T_3 (8)	NR	Expression levels	Mean (values in figure)	SEM (values in	NR	↑SOD activity in

al., 2015 [55]	T_2: Almond beverage (+ vitC + vitE)<br>T_3: T_1 + T_2		↑*SOD2* (*p*<0.05, C)<br>↓*GSR* (*p*<0.05, C)<br>(NC) *GPX1*<br>Lymphocytes<br>(NC) in any of the genes studied					figure)		neutrophils in T_1 (post- *vs* pre- and compared to C).<br>NS↓ SOD with T_3<br>NS↓ GSR in C or T_3
Marotta F et al, 2010 [39]	Fermented papaya	Neutrophils	↑*SOD1, CAT, GPX1, OGG1*<br>(all *p*<0.05, post- *vs* pre-)	T (11)	NR	Arbitrary units (relative expression levels)	Unclear (values only in figures; no association between *GSTM1* and *OGG1* genotype and changes in gene expression)	NR	NR	NR
Yanaka A et al., 2009 [15]	Broccoli sprout (SFGluc)	Polymorpho-nuclear granulocytes	↑*HMOX1* (FC= 2 to 3)	T (few volunteers)	NR	Change (FC)	NR	NR	NR	NR
Boesch-Saadatmandi C et al., 2009 [77]	Quer	CD14 + monocytes	(NC) *PON2*	T (20)	NR	Relative expression levels	Mean (values only in figure)	SEM (values only in figure)	No association with plasma quercetin	NR
Nieman DC et al., 2007 [76]	Quer	White blood cells, skeletal muscle	Leukocyte:<br>↓*CXCL8* (-33%, *p* = 0.019, T *vs* C post- exercise)<br>↓*IL10* (*p*= 0.012, T *vs* C post-exercise)<br>(NC) *IL1RN*<br>Muscle:<br>(NC) *PTGS2, IL6, CXCL8, IL1B, TNF* (T *vs* C, post-exercise)	T (20), C (20)	T (20) *vs* C (20) (post-exercise: 3h cycling)	Change (FC)	Mean (values only in figures)<br>Change % (in text)	SEM (values only in figures)	NR	(NS↓) IL-8 and TNF in plasma in T *vs* C (day 1 post-exercise
**Gastrointestinal tissue samples**										
Mallery SR et al., 2008 [46]	Freeze-dried black raspberry gel (10% w/w)	Oral intra-epithelial neoplasia and normal ventral-lateral tongue	Several genes examined: *KRT76, DSC1, UGT2B28, KSR1, PPP2CA, TMPRSS11E, SPRR3, UBD, TGM1, LOR, KRT13, SPRR2C* (high heterogeneity in the results). Examples:<br>*PTGS2* (↓ n=11, ↑ n=9)<br>*NOS2* (↓ n=12, ↑ n=8)<br>*VEGFA* (↓ n=14, ↑ n=6)	T (20 with neoplasia, 10 healthy)	NR	Change (FC)	Individual values (attempt to stratify in high and low responders)	NR	NR	High inter-individual variability<br>↓PTGS2 (*p*<0.005, 17/20 patients)<br>↓NOS2 (12/20 patients)

Turowski JB et al., 2015 [56]	Flaxseed (subgroups population: healthy; cystic fibrosis patients)	Buccal swabs	↓ ***HMOX1*** (*p*=0.026, healthy, post- *vs* pre-)<br>(NS↑) *HMOX1* (cystic fibrosis, post- *vs* pre-)<br>(NC) *NQO1* (healthy, post- *vs* pre-)<br>(NS↑) *NQO1*(cystic fibrosis, post- *vs* pre-)	T (5 healthy, 10 with cystic fibrosis)	NR	Change (FC)	Unclear (values only in figures)	Unclear (values only in figures)	Stratification of cystic fibrosis patients in low plasma lignans and high plasma lignans: NS differences associated to the levels of lignans	NR
Knobloch TJ et al., 2016 [48]	Black raspberry powder (in topically applicable troches)	Oral cancer and distal normal high risk oral mucosa	↓***AURKA, BIRC5, EGFR, NFKB1, PTGS1*** (*p*<0.05 in tumour samples post- *vs* pre- after multiple comparison adjustment by disease stage, BMI, smoking, age)	T (33)	NR	Change (FC)	NR	Individual data for some genes, 90 % CI	NR	NR
Gasper AV et al., 2007 [12]	Broccoli drink (SFGluc)<br>(T_1: high SFGluc<br>T_2: standard SFGluc)	Gastric antrum	↑***GCLM*** ($p \leq 0.05$, T_1)<br>↑***TXNRD1*** ($p \leq 0.001$ T_1, $p \leq 0.05$ T_2)<br>(NC) *CDKN1A*	T_1 (6-10), T_2 (6-10), C (6-10)	NR	Change (log2 FC)	Mean (values only in figures)	SEM (values only in figures)	NR	NR
Koosirat C et al., 2010 [82]	Turmeric (containing Cur)	Gastric antrum	(NC) *CXCL8, IL1B, TNF, PTGS2* (T, post- *vs* pre-)<br>↓ ***CXCL8*** (C, post- *vs* pre-)<br>↓ ***CXCL8, PTGS2*** (*p*=0.0008, *p*=0.04, T *vs* C post-)	C (19), T (17)	C (19) *vs* T (17)	Expression levels (fold-decrease values)	Mean (some values in text and figures, plots of individual values and changes).	SD (values in text)<br>95% CI (values in figures)<br>(% individuals with a change in text)	NR	NR
Labonté M-E et al., 2013 [61]	Fish oil (EPA + DHA)	Duodenal tissue	(NC) *IL6, TNF, IL18, STAT3*	NR	C (12) *vs* T (12)	Nº copies/10^5 copies of the ref gene	Median	IQR	NR	NR
Frommel TO et al., 1994 [66]	β-carotene	Colon tissue	(NS↑) *GJA1* (in 4 individuals after T but only in 1 after C_1/ C_2)	T (6) , C_{1+2}(8)	NR	Scoring system of intensity	NR	NR	No correlation between tissue β-carotene and *GJA1* but some association in some subjects	NR
Nguyen AV et al., 2009 [42]	Res, Quer, grape powder<br>T_1: high Res + Quer<br>T_2: low Res + Quer<br>T_3: high grape	Colon tissue (cancer, normal)	Normal tissue<br>↑***MYC*** ($p = 0.01$, T_4)<br>↓***CCND1*** ($p < 0.005$, T_4)<br>↓***AXIN2*** ($p < 0.05$, T_4)<br>Cancer tissue<br>↑***MYC*** ($p < 0.01$, T_4)<br>↑***CCND1*** ($p < 0.05$, T_4)	T_1 (1), T_2 (2), T_3 (2), T_4 (3)	NR	Expression levels	Unclear (values only in figures)	Unclear (values only in figures)	NR	NR

	powder<br>T_4: low grape powder									
Ishikawa H et al., 2012 [41]	Propolis (Atrepillin C, polyphenols)	Colon mucosa	↑*CCND1* (*p*=0.018, T)<br>(NC) *PCNA, BAX*	T (15), C (15)	T (15) *vs* C (15)	Expression levels (also fold of control, unclear)	Mean (individual values in figures)	95% CI	NR	NR
Nuñez-Sanchez MA et al., 2017 [50]	Pomegranate extract (ETs) (T_1,T_2, different doses of ETs components analysed as one T)	Colon tissue (cancer, normal)	↓*CD44, CTNNB1, CDKN1A EGFR, TYMS*<br>(different comparisons and different *p*-values; counterbalance effect in normal and cancer tissue)<br>(NC) *MYC, CASP3, KRAS*	T_{1+2} (35) , C (10)	T_{1+2} (35) *vs* C (10)	Expression levels, change (FC)	Median	Range (inter-individual variability study)	No association found with levels of metabolites (urolithins) in colon tissues or in urine (metabotypes)	NR
**Other tissue samples**										
González-Sarrías A et al., 2010 [30]	Walnuts and pomegranate juice (ETs)<br>T_1: walnuts (ETs),<br>T_2: pomegranate juice (ETs)	Prostate tissue (benign hyperplasia and cancer tissues)	(NC) *CDKN1A, MKI67, MYC*	NR	C (30) *vs*<br>T_1 (14)+T_2 (19),	Expression levels	Mean	SD, Range (box-plots representation of variability)	No association with prostate tissue metabolites (urolithins derivatives)	NR
Chan JM et al., 2011 [21]	T_1: lycopene in soy oil + olive oil<br>T_2: fish oil + soy oil + olive oil	Prostate tissue (normal)	(NC) *IGF1, IGF1R, PTGS2*	C (26), T_1 (22), T_2 (21)	C (26) *vs* T_1 (22),<br>C (26) *vs* T_2 (21)	Change (FC)	Mean	SD	NR	NR
Lazarevic B et al., 2012 [79]	Genistein	Prostate tissue (cancer, normal)	↓*KLK4* (*p*=0.033 in cancer tissue, *p*=0.087 in normal tissue, T *vs* C, *p*=0.041 in T normal *vs* cancer)<br>(NS↓) *CDKN1A* (cancer tissue, T *vs* C, *p*=0.184)<br>(NC) *AR, NKX3-1, CDKN1B, TP53*	NR	T (10) *vs* C (12)<br>T (normal *vs* cancer), C (normal *vs* cancer)	Expression levels	Mean (values only in figures)	SEM (values only in figures)	NR	NR
Most J et al., 2015 [81]	EGCG	Adipose tissue	↑*LEP* (*p*=0.05)<br>(NS↑) *CD36*<br>(NC) *CPT1A, PNPLA2, LIPE, ACACA*	NR	T (24) *vs* C (24)<br>(6 h post-prandial)	Expression levels	Mean	SEM	NR	NR
Kruse M et al., 2015 [29]	T_1: rapeseed/canola oil (MUFA, PUFA)<br>T_2: Olive oil (MUFA) and two	Adipose tissue	Chronic:<br>↓*IL6* (*p*=0.001, T_1 *vs* T_2)<br>(NC) *IL1B, CCL2, ADGRE1, CXCL8, IL10, SERPINE1, TNF* (T_1 *vs* T_2)<br>Acute:	T_1 (9), T_2 (9)	T_1 (9) *vs* T_2 (9)	Arbitrary units, change (%, FC)	Mean (values only in figure)	SEM (values only in figure)	NR	Chronic:<br>(NC) serum IL6, CCL2<br>Acute:<br>↑serum IL6 in T_1, T_2 (NS)

	periods/doses (chronic, acute)		**↑*IL6 (p = 0.032, T_1)*** **↑*IL1B (p= 0.030 T_1)*** **↑*ADGRE1*** (*p*=0.049, T_1) **↑*CCL2*** (*p*=0.009, T_1, *p*=0.043, T_2) (NC) *IL1B, IL6, CCL2, ADGRE1, CXCL8, IL10, SERPINE1, TNF* (T_1 *vs* T_2)							(NC) CCL2
Poulsen MM et al., 2013 [70]	Res	Skeletal muscle and adipose tissue	Muscle **↓ *SLC2A4*** (p<0.05, T) (NC) *PPARGC1A* Adipose (NC) *TNF, NFKB1*	T (12), C (12)	T (12) *vs* C (12)	Expression levels	Mean (values only in figure)	SEM (values only in figure)	NR	NR
Yoshino J et al., 2012 [69]	Res	Skeletal muscle and adipose tissue	(NC) *SIRT1, NAMPT, PPARGC1A, UCP3*	T (8-12), C (8-12)	T(8-12) *vs* C (8-12)	Expression levels	Mean (values only in figure)	SEM (values only in figure)	NR	NR
Kerksick CM et al., 2013 [80]	T_1: NAC T_2: EGCG	Skeletal muscle	(NC) *TRIM63, FBXO32, PSMA1, PSMA2, UBE3B, CAPN2, CAPN1*	C (10), T_1 (10), T_2 (10) (different time points)	C (10) *vs* T_1 or T_2 (10)	Change (FC)	Mean	SEM	NR	NR
Olesen J et al., 2014 [71]	Res (+physical exercise in 2 of 4 groups)	Skeletal muscle	(NC) *PPARGC1A, TNF, NOS2*	T (9) , C (7) $T_{exercise}$ (14), $C_{exercise}$ (13)	T (9) *vs* C (7), $T_{exercise}$ (14) *vs* $C_{exercise}$ (13)	Change (FC)	Mean (values only in figure)	SEM (values only in figure)	NR	(NC) TNF, NOS2 in muscle and plasma in T
Riedl MA et al., 2009 [14]	Broccoli sprout (SFGluc) (different doses)	Cells from nasal lavage	**↑*GSTM1, GSTP1, NQO1, HMOX1*** (dose-response from T_4-T_8, *p* ≤0.001 ; *p* ≤0.004, T_8 *vs* C)	T (3-10)	T_8 (8) *vs* C (5)	Change (%)	Mean	SD	NR	NR
Knott A et al., 2008 [51]	*Arctium lappa* fruit extract (Arctiin) topical application)	Suction blister epidermis	**↑*HAS2*** (*p*=0.018)	NR	T (6) *vs* C_1 (6)	Expression levels	Mean	SD	NR	↑27.1% hyaluronan levels T *vs* C (post-)
Marini A et al., 2014 [63]	Lycopene + β-carotene + probiotic (*Lactobacillus johnsonii)*	Skin	Unclear effects on *ICAM-1* (T *vs* C, post-)	T (29-30), C (29-30)	T (29-30) *vs* C (29-30)	NR	Unclear (values only in figure)	Unclear (values only in figure)	NR	NR
Marini A et al., 2012 [54]	Pine bark extract Pycnogenol (procyanidins)	Buttock skin	**↑*HAS1*** (*p*<0.001) (NS↑) *COL1A1, COL1A2*	T (20)	NR	Change (%)	Mean (values only in figure)	SEM (values only in figure)	NR	NR
Farris P et al., 2014 [64]	Res + baicalin + vitE (topical application)	Photo-damaged face skin	**↑ *HMOX1, COL3A1*** **↓ *VEGFA*,** (NC) *COL1A1, PRKAA1, SOD1*	T (10)	NR	Change (FC)	NR	NR	NR	NR

Bertuccelli G. et al., 2016 [40]	Fermented papaya (sublingual dose)	Forearm skin	↑*AQP3* (*p*<0.05, T post- *vs* pre-, T *vs* C)<br>↓ *PPIA, CD47* (*p*<0.05, T post- *vs* pre-, T *vs* C)<br>(NS↓) *LMNA* (*p*= 0.068, *T*)	C (30), T (30)	C (30) *vs* T (30)	Expression levels	Unclear (values only in figure)	Unclear (values only in figure)	NR	NR

[1] White blood cells or leukocytes: mononuclear cells agranulocytes (lymphocytes and monocytes) and polymorphonuclear granulocytes (neutrophils, eosinophils, basophils, mast cells); lymphocytes (mostly T cells, B cells and NK cells, also some dendritic cells); leukemic blasts (myeloblasts or immature white blood-forming cells).

Table abbreviations (in alphabetical order): BMI, body mass index; C, control; Cam, campesterol; Carn, carnitine; CGA: chlorogenic acid; CI, confidence interval; Cur, curcumin; DHA, docosaxehaenoic acid; EGCG, epigallocatechin gallate; EPA, eicosapentaenoic acid; ETs, ellagitannins; FC, fold-change; FruVeg, fruits and vegetables; HbF, fetal hemoglobin; Hbg, hemoglobin; HTyr, hydroxytyrosol; IQR, interquartile range; Lath, Lathosterol; LDL, low-density lipoprotein; McD, Macdonald; Med, Mediterranean; MNC, mononuclear cells; MUFA, monounsaturated fatty acids; NAC, N-acetyl-cysteine; NC, no change; NMP, *N*-methylpyridinum; NR, not reported; NS, not significant; post-, after treatment; pre-, baseline or before treatment; PUFA, polyunsaturated fatty acids; Quer, quercetin; Res, resveratrol; SD, standard deviation; Se, selenium; SEM, standard error of the mean; SFGluc, sulforaphane glucosinolates; T, treatment; Tyr, tyrosol; UVA, ultraviolet A; Veg, vegetables; VitC, vitamin C; VitE, vitamin E; *8-oxodG*, 8-oxo-7,8-dihydro-2'–deoxyguanosine.

Genes nomenclature from GeneCards [89] (in alphabetical order): *ABCA1*, ATP binding cassette subfamily A member 1; *ABCG1,* ATP binding cassette subfamily G member 1; *ACACA*, acetyl-CoA carboxylase alpha (alias: *ACC1*); *ACE*, angiotensin I converting enzyme; *ADGRE1*, adhesion G protein-coupled receptor E1 (alias:*EMR1*); *ADRB2*, adrenoceptor beta 2; *AHR*, aryl hydrocarbon receptor; *AHRR*, aryl-hydrocarbon receptor repressor; *ALOX5AP*, arachidonate 5-lipoxygenase activating protein; *APEX1*, apurinic/apyrimidinic endodeoxyribonuclease 1; *AQP3*, aquaporin 3 (Gill blood group); *AR*, androgen receptor; *ARHGAP15*, rho GTPase activating protein 15; *AURKA*, aurora kinase A; *AXIN2*, axin 2; *BAX*, BCL2 associated X, apoptosis regulator; *BCL2*, BCL2, apoptosis regulator; *BIRC5*, baculoviral IAP repeat containing 5; *CALR*, calreticulin (alias: *CRT*); *CAPN1*, calpain 1; *CAPN2*, calpain 2; *CASP3*, caspase 3; *CAT*, catalase; *CCL2*, C-C Motif Chemokine Ligand 2 (alias: *MCP-1*, monocyte chemotactic protein 1); *CCL3*, C-C motif chemokine ligand 3; *CCL5*, C-C motif chemokine ligand 5; *CCND1*, cyclin D1; *CD14*, CD14 molecule; *CD36*, CD36 molecule; *CD40LG*, CD40 ligand; *CD44*, CD44 molecule (Indian blood group); *CD47*, CD47 molecule; *CDKN1A*, cyclin dependent kinase inhibitor 1A (alias: *p21*); *CDKN1B*, cyclin dependent kinase inhibitor 1B (alias: *p27*); *COL1A1*, collagen type I alpha 1 chain; *COL1A2*, collagen type I alpha 2 chain; *COL3A1*, collagen type III alpha 1 chain; *CPT1A*, carnitine palmitoyltransferase 1A; *CPT1B*, carnitine palmitoyltransferase 1B; *CRAT*, carnitine O-acetyltransferase; *CTNNB1*, catenin beta 1; *CXCL8*, C-X-C motif chemokine ligand 8 (alias: *IL8*); *CXCR1*, C-X-C motif chemokine receptor 1; *CXCR2*, C-X-C motif chemokine receptor 2 (alias: *IL8RA*); *CYBA*, cytochrome B-245 alpha chain (alias: *P22-Phox*); *CYBB*, cytochrome B-245 beta chain (alias: *NOX2, GP91-Phox*); *CYP1A1*, cytochrome P450 family 1 subfamily A member 1; *DSC1*, desmocollin 1; *DUOX2*, dual oxidase 2; *ECE2*, endothelin converting enzyme 2; *EGFR*, epidermal growth factor receptor; *EGR1*, early growth response 1; *E2F1*, E2F transcription factor 1; *ERCC1*, ERCC excision repair 1, endonuclease non-catalytic subunit; *ERCC2*, ERCC excision repair 2, TFIIH core complex helicase subunit; *ERCC4,* ERCC excision repair 4, endonuclease catalytic subunit; *FBXO32*, F-box protein 32 (alias: *Atrogin1*); *FOSL1*, FOS like 1, AP-1 transcription factor subunit (alias: *FRA-1*); *FXN*, frataxin, Friedreich ataxia protein; *GCLC*, glutamate-cysteine ligase catalytic subunit (alias: *γGCL*); *GCLM*, glutamate-cysteine ligase modifier subunit; *GJA1*, gap junction protein alpha 1; *GPX1*, glutathione peroxidase 1; *GPX4*, glutathione peroxidase 4; *GPX7*, glutathione peroxidase 7; *GSTA1*, glutathione S-transferase alpha 1; *GSTA4*, glutathione S-transferase alpha 4; *GSTK1*, glutathione S-transferase kappa 1; *GSTM1*, glutathione S-transferase Mu 1; *GSTM2*, glutathione S-transferase Mu 2; *GSTM3*, glutathione S-transferase Mu 3; *GSTM4*, glutathione S-transferase Mu 4; *GSTM5*, glutathione S-transferase Mu 5; *GSTO1*, glutathione S-transferase omega 1; *GSTO2*, glutathione S-transferase omega 2; *GSTP1*, glutathione S-transferase Pi 1 (alias: *GSTP1-1*); *GSR*, glutathione-disulfide reductase (alias: *GRD1*); *GSTT1*, glutathione S-transferase theta 1; *HAS1*, hyaluronan synthase 1; *HAS2*, hyaluronan synthase 2; *HBG1*, hemoglobin subunit gamma 1; *HMGA1*, high mobility group at-hook 1; *HMGCR*, 3-hydroxy-3-methylglutaryl-CoA reductase; *HMOX1*, heme oxygenase 1 (alias:*HO-1*); *HNMT*, histamine N-methyltransferase; *HSPA5*, heat shock protein family A (Hsp70) member 5 (alias: *BIP*); *ICAM1*, intercellular adhesion molecule 1; *ID3*, inhibitor of DNA binding 3, HLH protein; *IGF1*, insulin like growth factor 1; *IGF1R*, insulin like growth factor 1 receptor; *IFNG*, interferon gamma; *IKBKB*, inhibitor of nuclear factor kappa B kinase subunit

beta (alias: *IKKβ*); *IL1B*, interleukin 1 beta; *IL1RN*, interleukin 1 receptor antagonist; *IL6*, interleukin 6; *IL7R*, interleukin 7 receptor; *IL10*, interleukin 10; *IL18*, interleukin 18; *IL23A*, interleukin 23 subunit alpha; *INMT*, indolethylamine N-methyltransferase; *IRAK1*, interleukin 1 receptor associated kinase 1; *IRS1*, insulin receptor substrate 1; *JUN*, jun proto-oncogene, AP-1 transcription factor subunit; *KLK-L1*, kallikrein-like protein 1; *KLK4*, kallikrein related peptidase 4; *KRAS*, KRAS proto-oncogene, GTPase; *KRT13*, keratin 13; *KRT76*, keratin 76; *KSR1*, kinase suppressor of Ras 1; *LDLR*, low density lipoprotein receptor; *LEP*, leptin; *LIPE*, lipase E, hormone sensitive type (alias: *HSL*); *LMNA*, lamin A/C (truncated LMNA: *Progerine*); *LOR*, loricrin; *LPL*, lipoprotein lipase; *LRRFIP1*, LRR binding FLII interacting protein 1; *MAPK8*, mitogen-activated protein kinase 8 (alias: *JKN1*); *MCL1*, MCL1, BCL2 family apoptosis regulator; *MED1*, mediator complex subunit 1 (alias: *PPARBP*); *MGMT*, O-6-methylguanine-DNA methyltransferase; *MGST1*,microsomal glutathione S-transferase 1; *MGST2*, microsomal glutathione S-transferase 2; *MGST3*, microsomal glutathione S-transferase 3; *MKI67*, marker of proliferation Ki-67; *MMP2*, matrix metallopeptidase 2; *MPO*, myeloperoxidase; *MYC*, MYC proto-oncogene, BHLH transcription factor; *NAMPT*, nicotinamide phosphoribosyltransferase; *NCF1* , neutrophil cytosolic factor 1 (alias: *p47-Phox*); *NFE2L2*, nuclear factor, erythroid 2 like 2 (alias: *NRF2*); *NFKB1*, nuclear factor kappa B subunit 1; *NFKBIA*, NFKB inhibitor alpha; *NKX3-1*, NK3 homeobox 1 (alias: *NK3X1*); *NQO1*, NAD(P)H quinone dehydrogenase 1; *NQO2*, N-ribosyldihydronicotinamide:quinone reductase 2; *NOS2*, nitric oxide synthase 2 (alias: *iNOS*); *NR1H2*, nuclear receptor subfamily 1 group H member 2; *NUDT1*, nudix hydrolase 1; *OGG1*, 8-oxoguanine DNA glycosylase; *OLR1*, oxidized low density lipoprotein receptor 1; *PCNA*, proliferating cell nuclear antigen; *PNPLA2*, patatin like phospholipase domain containing 2 (alias: *ATGL*); *POLK*, DNA polymerase kappa; *POLR2A*, RNA polymerase II subunit A; *PON2*, paraoxonase 2; *PPARA*, peroxisome proliferator activated receptor alpha; *PPARD*, peroxisome proliferator activated receptor delta; *PPARG*, peroxisome proliferator activated receptor gamma; *PPARGC1A*, PPARG coactivator 1 alpha (alias: *PGC1α*); *PPIA*, peptidylprolyl isomerase A; *PPP2CA*, protein phosphatase 2 catalytic subunit alpha; *PRKAA1*, protein kinase AMP-activated catalytic subunit alpha 1; *PSMA1*, proteasome subunit alpha 1 (alias: *HC2*); *PSMA2*, proteasome subunit alpha 2 (alias: *HC3*); *PTGS1*, prostaglandin-endoperoxide synthase 1 (alias: *COX1*); *PTGS2*, prostaglandin-endoperoxide synthase 2 (alias: *COX2*); *PTPN1*, protein tyrosine phosphatase, non-receptor type 1 (alias: *PTP1B*); *RETN*, resistin; *SCARB1*, scavenger receptor class B member 1 (alias: *SRB1*); *SELENOF*, selenoprotein F; *SELENOP*, selenoprotein P; *SELENOS*, selenoprotein S; *SERPINE1*, serpin family E member 1; *SIRT1*, sirtuin 1; *SIRT2*, sirtuin 2; *SLC2A3*, solute carrier family 2 member 3; *SLC2A4*, solute carrier family 2 member 4 (alias: *GLUT4*); *SLC22A5*, solute carrier family 22 member 5 (alias: *OCTN2*); *SOCS3*, suppressor of cytokine signaling 3; *SOD1*, superoxide dismutase 1 (alias: *Cu/ZnSOD*); *SOD2*, superoxide dismutase 2 (alias: *MnSOD*); *SPRR2C*, small proline rich protein 2C (pseudogene); *SPRR3*, small proline rich protein 3; *STAT3*, signal transducer and activator of transcription 3; *TGM1*, transglutaminase 1; *TIMP1*, TIMP metallopeptidase inhibitor 1; *TIMP2*, TIMP metallopeptidase inhibitor 2; *TLR2*, toll like receptor 2; *TLR4*, toll like receptor 4; *TMPRSS11E*, transmembrane protease, serine 11E; *TNF*, tumor necrosis factor (alias: *TNFα*); *TNFRSF1A*, TNF receptor superfamily member 1A; *TNFSF4*, TNF Superfamily Member 4; *TNFSF10*, TNF superfamily member 10; *TP53*, tumor protein P53 (alias: *p53*); *TRAF3*, TNF receptor associated factor 3; *TRIM63*, tripartite motif containing 63 (alias: *MURF1*); *TXN*, thioredoxin; *TXNRD1*, thioredoxin reductase 1 (alias: *TR1*); *TYMS*, thymidylate synthetase; *UBD*, ubiquitin D; *UBE3B*, ubiquitin protein ligase E3B; *UCP2*, uncoupling protein 2; *UCP3*, uncoupling protein 3; *UGT2B28*, UDP glucuronosyltransferase family 2 member B28; *VCAM1*, vascular cell adhesion molecule 1; *VEGFA*, vascular endothelial growth factor A (alias: *VEGF*); *VEGFB*, vascular endothelial growth factor B; *XBP1*, X-box binding protein 1; *XPA*, XPA DNA damage recognition and repair factor; *XPC*, XPC complex subunit, DNA damage recognition and repair factor; *XRCC1*, X-ray repair cross complementing 1; *XRCC3*, X-ray repair cross complementing 3.

Table S4. Summary of the expression changes in *TNF* as determined in human cell or tissue samples following intervention with diets, foods or derived products rich in bioactive compounds or with single bioactive compounds. The type and quality of the data presented in the study, estimation of or information about the variability as well as information about the association between the observed effects with the presence of bioactive metabolites and (or) with the corresponding protein quantity/activity are indicated.

Reference	Intervention (daily dose, duration)	Effect potentially associated to the intervention with the bioactive source, (cells[1], tissue, sample size)	*TNF* expression results in human clinical studies			Association with	
			Data presentation	Data quality[2]	Variability information[3]	Compounds/ Metabolites (urine, plasma, tissue)	Protein levels and (or) activity, tissue
Diets rich in bioactive compounds							
Marques-Rocha JL et al., 2016 [10]	Hypocaloric Med-based diet (56 d)	No effect (white blood cells, n=40)	• C: not included<br>• T: data presented as FC (mean ± SD)<br>- Post-: 1.53 ± 1.25, pre: 1.01 ± 0.86; FC (post- *vs* pre-): NS (*p*-value=0.08)	Poor (control group not included)	Calculated CV<br>post- T: CV=81.7%<br>pre- T: CV= 85.1%	NR	(NC) TNF in plasma
Di Renzo L et al., 2017 [34]	T_1: McD meal+ hazelnuts (40 g)<br>T_2: McD meal<br>3 h	↓*TNF* (blood, n=22)	• C: not included<br>• Comparisons between treatments T_1 or T_2 and no dietary treatment (pre- *vs* post-) and between T_1 and T_2 (post-)<br>- T_2 (pre- *vs* post-): FC<-1.5 ($p<0.05$)<br>- T_1 (pre- *vs* post-): NC<br>- T_2 *vs* T_1: FC>+1.5 ($p<0.05$)	Poor (data only in figures; unclear data reporting)	No information available	NR	NR
Grapes and derived products containing bioactive compounds							
Weseler AR et al., 2011 [43]	Grape seeds (flavanols) (200 mg/d, 56 d)	↓*TNF* (blood, n=15)	• C: not included<br>• T: data presented as % of change<br>- (post- *vs* pre-) Change=-12%; *p*-value<0.05	Poor (control not included; data in figures)	No information available	NR	Inhibition of *ex vivo* LPS-induced TNF in blood
Tomé-Carneiro J et al., 2013 [45]	T_1: Grape extract (151 mg/d, 183 d)	No effect (mononuclear cells, n=9-13)	• C: data presented as Ratio (median, IQR)<br>- Ratio (post- *vs* pre-): 0.83 (0.57-1.25) (*p*-value=0.239)<br>• T: data presented as Ratio (median, IQR)<br>- Ratio (post- *vs* pre-): 0.78 (0.58-1.59) (*p*-value=0.775)	High	IQR	NR	(NC) TNF in serum or plasma
	T_1: Grape extract (302 mg/d, 365 d)		• C: data presented as Ratio (median, IQR)<br>- Ratio (post- *vs* pre-): 0.80 (0.70-1.35) (*p*-value=0.890)<br>• T: data presented as Ratio (median, IQR)<br>- Ratio (post- *vs* pre-): 0.86 (0.37-1.31) (*p*-value=0.083)				
	T_2: Grape extract (151 mg + 8 mg	↓*TNF* (mononuclear cells, n=9-13)	• C: data presented as Ratio (median, IQR)<br>- Ratio (post- *vs* pre-): 0.83 (0.57-1.25) (*p*-value=0.239)<br>• T: data presented as Ratio (median, IQR)				

	Res/d, 183 d)		Ratio (post- *vs* pre-): 0.39 (0.25-1.23) (*p*-value=0.016)				
	T_2: Grape extract (302 mg + 16 mg Res/d, 365 d)		• C: data presented as FC (median, IQR)<br>- Ratio (post- *vs* pre-): 0.80 (0.70-1.35) (*p*-value=0.890)<br>• T: data presented as Ratio (median, IQR)<br>- Ratio (post- *vs* pre-): 0.65 (0.22-1.02) (*p*-value=0.019)<br>• T *vs* C: data presented as Ratio (median): 0.54 (-46%) (*p*-value=0.06)				
Poulsen MM et al., 2013 [70]	Res (1500 mg/d, 28 d)	No effect (adipose tissue, n=12)	• C: data presented as expression levels (mean, SEM)<br>- Post-: 0.00039 (0.000025), pre-: 0.00038 (0.00005)<br>• T: data presented as expression levels (mean, SEM)<br>- Post-: 0.00047 (0.000035), pre-: 0.00036 (0.000025)<br>(values estimated from figures)	Medium (data only in figures)	Calculated CV<br>post- C: CV=22.2%<br>pre- C: CV= 45.6%<br>post- T: CV=25.8%<br>pre- T: CV= 24.1%	NR	(NC) TNF plasma (ND) in adipose tissue
Olesen J et al., 2014 [71]	Res (250 mg/d, 56 d)	No effect (skeletal muscle, n=7-14)	• C: data presented as expression levels (mean, SEM)<br>- Post-: 1.1 (0.26), pre-: 1.0 (0.11)<br>• T: data presented as expression levels (mean, SEM)<br>- Post-: 0.83 (0.17), pre-: 1.0 (0.2)<br>(values estimated from figures)	Medium (data only in figures)	Calculated CV<br>post- C: CV=62.5%<br>pre- C: CV= 29.1%<br>post- T: CV=61.4%<br>pre- T: CV= 60.0%	NR	(NC) TNF muscle & plasma
	Res + training (56 d)	Reverts the effect of exercise (skeletal muscle, n=7-14)	• C: data presented as expression levels (mean, SEM)<br>- Post-: 0.57 (0.11), pre-: 1.0 (0.17)<br>• T: data presented as expression levels (mean, SEM)<br>- Post-: 0.90 (0.14), pre-: 1.0 (0.1)		Calculated CV<br>post- C: CV=69.5%<br>pre- C: CV= 61.0%<br>post- T: CV=58.2%<br>pre- T: CV= 37.4%		
Oils rich in bioactive compounds							
Labonté M-E et al., 2013 [61]	Fish oil (EPA + DHA) (3g/d, 56 d)	No effect *TNF* (duodenal biopsies, n=12)	• C: data presented as Nº copies/10^5 copies ref gene (median, IQR)<br>- Post-: 109 (51)<br>• T: data presented as Nº copies/10^5 copies ref gene (median, IQR)<br>- Post-: 93 (50)<br>• T *vs* C (difference):-16 (*p*-value:0.75)	Poor (only post data)	IQR	NR	NR
Jamilian M et al., 2018 [62]	Fish oil (360 mg EPA + 240 mg DHA, 42 d)	↓*TNF* (mononuclear cells, n=20)	• C: placebo capsules<br>• T: data presented as relative Fold-change compared to placebo (log transformed data, unclear reporting, mean, SD)<br>• T *vs* C (difference):-1.12 (*p*-value:0.01)	Medium-Poor (poor data reporting, data only in figures)	Calculated CV<br>post- C: CV=10.5%<br>post- T: CV=18.0%	NR	NR
Vors C. et al., 2017 [67]	T_1: EPA (2.7g/d, 70 d)	↑*TNF* (blood, n=44)	• C: data presented as Nº copies/copies of ref gene (mean, SEM)<br>- Post-: 3110 (93)<br>• T: data presented as Nº copies/copies of ref gene (mean, SEM)<br>- Post-: 3329 (120)<br>• T *vs* C (difference):+219 (*p*-value:0.06)	Poor (only post- data)	Calculated CV<br>post- C: CV=19.8%<br>post- T: CV=23.9%<br>Individual data provided	NR	No correlation between *TNF* & plasma protein
	T_2: DHA (2.7g/d, 70 d)	↑*TNF* (blood, n=44)	• C: data presented as Nº copies/copies ref gene (mean, SEM)<br>- Post-: 3110 (93)<br>• T: data presented as Nº copies/copies ref gene (mean, SEM)<br>- Post-: 3392 (124)<br>• T *vs* C (difference):+282 (*p*-value:0.01)		Calculated CV<br>post- C: CV=19.8%<br>post- T: CV=24.2%<br>Individual data provided		
Kruse M et	T_1:rapeseed/	No difference	• C: not included	Poor (only	Calculated CV	NR	NR

al., 2015 [29]	canola oil (MUFA, PUFA) (50g/d, 28 d)	T_1 *vs* T_2 (adipose tissue, n=9)	• T: data presented as arbitrary units (mean, SEM)<br>- Post-: 0.55 (0.18)	post- data)	post- T: CV=98.1%		
	T_2: Olive oil (MUFA) (50g/d, 28 d)		• C: not included. T: data presented as arbitrary units (mean, SEM)<br>- Post-: 0.20 (0.05)		Calculated CV<br>post- T: CV=75.0%		
Other bioactive compounds							
Koosirat C et al., 2010 [82]	Turmeric tablet (Cur) (120 mg/d, 28 d)	No effect (gastric antrum, n=17-19)	• C: data presented as expression levels, FC (mean, 95% CI, SD), % individuals with a change<br>- Pre-: 0.20 (0.11-0.29); FC (post- *vs* pre-): -37.2 (34.06); 63.2% ↓*TNF*<br>• T: data presented as expression levels, FC (mean, 95% CI, SD), % individuals with a change<br>- Pre-: 0.50 (0.27-0.73); FC (post- *vs* pre-): -1.9 (0.38); 52.9% ↓*TNF*<br>- T *vs* C (difference): NS (stronger reducing effect in control group)	Medium-Poor (poor data reporting)	Calculated CV<br>pre- C: CV=109%<br>C (post- *vs* pre-): CV:91.6%<br>pre- T: CV=99%<br>T (post- *vs* pre-): CV: 20%	NR	NR
Nieman DC et al., 2007 [76]	Quer (1000 mg/d, 24 d)	No effect (skeletal muscle n=20)	• C: data presented as FC (mean, SEM)<br>- Post-: +3.5 (0.38);<br>• T: data presented as FC (mean, SEM)<br>- Post-: +5.0 (0.75)<br>T *vs* C (difference): NS (p-value:0.930)	Poor (only post- data)	Calculated CV<br>post- C: CV=48.5%<br>post- T: CV=67.0%	NR	NR

[1]**White** blood cells or Leukocytes**:** mononuclear cells agranulocytes (lymphocytes and monocytes) and polymorphonuclear granulocytes (neutrophils, eosinophils , basophils, mast cells)**;**[2]Data quality based on: absence of proper control group, poor or confusing data reporting, results only in figures;
[3]Coefficient of variation calculated when data (mean, SD, SEM) available.

Table abbreviations (in alphabetical order): C, control group; CI, confidence intervals; Cur, curcumin; CV, coefficient of variation; d, days; DHA, docosahexaenoic acid;EPA, eicosapentaenoic acid; FC, fold-change; h, hours; IQR, interquartile range; LPS, lipopolysaccharide; McD, MacDonald; Med, Mediterranean; MUFA, monounsaturated fatty acids; NC, no change; ND, not detected; NR, not reported; NS, not significant; post-, after treatment; pre-, baseline or before treatment; PUFA, polyunsaturated fatty acids; Quer, quercetin; ref gene, reference gene; Res, resveratrol; SD, standard deviation; SEM, standard error of the mean; T, treated group; *TNF*, tumour necrosis factor alpha (alias: *TNFα*).

Table S5. Summary of the expression changes in the *PPAR* family of genes as determined in human cell or tissue samples following intervention with diets, foods or derived products rich in bioactive compounds or with single bioactive compounds. The type and quality of the data presented in the study, estimation of or information about the variability as well as information about the association between the observed effects with the presence of bioactive metabolites and (or) with the corresponding protein quantity/activity are indicated.

Reference	Intervention (daily dose, duration)	Effect potentially associated to the intervention with the bioactive source, (cells[1], tissue, sample size)	*PPARs* expression results in human clinical studies			Association with	
			Data presentation	Data quality[2]	Variability information[3]	Compounds/ Metabolites (urine, plasma, tissue)	Protein levels and (or) activity, tissue
**Mixed products rich in bioactive compounds**							
Radler U et al., 2011 [65]	Low-fat yoghurt (grapeseed extract + fish oil + phospholipids + carnitine + VitC + VitE) (125g ×2/d, 84 d)	↑*PPARG* (mononuclear cells, n=20-22)	• C: data not shown<br>- No change indicated in the text<br>• T: data presented as expression levels (mean, SD)<br>- Post-: 2.53 (1.26), pre-: 1.0 (NR)<br>(values estimated from figures)	Poor (Control data not included; data only in figures)	Calculated CV<br>post- T: CV= 49.8%	NR	NR
**Oils rich in bioactive compounds**							
Vors C et al., 2017 [67]	T_1: EPA (2.7g/d, 70 d)	↑*PPARA* (blood, n=44)	• C: data presented as Nº copies/copies of ref (mean, SEM)<br>- Post-: 411.6 (16.2)<br>• T: data presented as Nº copies/copies of ref (mean, SEM)<br>- Post-: 461.4 (12.5)<br>• T *vs* C (difference): +49.8 (*p*-value:0.003)	Poor (only post- data)	Calculated CV<br>post- C: CV=26.1%<br>post- T: CV= 17.9%<br>Individual data provided	NR	NR
	T_2: DHA (2.7g/d, 70 d)		• C: data presented as Nº copies (mean, SEM)<br>- Post-: 411.6 (16.2)<br>• T: data presented as Nº copies (mean, SEM)<br>- Post-: 454.7 (13.4)<br>• T *vs* C (difference): +43.1 (*p*-value:0.01)		Calculated CV<br>post-C: CV=26.1%<br>post-T: CV= 19.5%<br>Individual data provided		
	T_1: EPA (2.7g/d, 70 d)	No effect *PPARG* (blood, n=44)	• C: data presented as Nº copies/copies of ref (mean, SEM)<br>- Post-: 46.0 (3.5)<br>• T: data presented as Nº copies/copies of ref (mean, SEM)<br>- Post-: 44.1 (3.5)<br>• T *vs* C (difference): -1.9 (*p*-value:0.30)		Calculated CV<br>post-C: CV=50.5%<br>post-T: CV= 52.6%		
	T_2: DHA (2.7g/d, 70 d)		• C: data presented as Nº copies (mean, SEM)<br>- Post-: 46.0 (3.5)<br>• T: data presented as Nº copies (mean, SEM)<br>- Post-: 48.0 (3.9)<br>• T *vs* C (difference): -2.0 (*p*-value:0.68)		Calculated CV<br>post-C: CV=50.5%<br>post-T: CV= 53.9%		

Jamilian M et al., 2018 [62]	Fish oil (360 mg EPA + 240 mg DHA, 42 d)	↑*PPARG* (mononuclear cells, n=20)	• C: placebo capsules<br>• T: data presented as relative Fold-change compared to placebo (log transformed data, unclear reporting, mean, SD)<br>• T *vs* C (difference):+1.06 (*p*-value: 0.04)	Medium-Poor (poor data reporting, data only in figures)	Calculated CV<br>post-C: CV=9.0%<br>post-T: CV=11.3%	NR	NR
Farràs M al., 2013 [23]	Olive oil (T_1: 26.2 mg polyphenols T_2: 8.0 mg polyphenols, postprandial response 5 h)	↑*PPARG* (white blood cells, n=13)	• T: data presented as the ratio T_1/T_2 (geometric mean, 95%CI)<br>- FC (T_1/T_2): 2.8 (1.7-4.6) (*p*-value<0.05)<br>(values estimated from figures)	Poor (Control not included; post- *vs* pre-comparison not included; data only in figures)	Calculated CV<br>T_1/T_2: CV=118.3%	NR	NR
		↑*PPARA* (white blood cells, n=13)	• T: data presented as the ratio T_1/T_2 (geometric mean, 95%CI)<br>- FC (T_1/T_2): 2.0 (1.5-2.6) (*p*-value<0.05)<br>(values estimated from figures)		Calculated CV<br>T_1/T_2: CV= 55.2%		
		↑*PPARD* (white blood cells, n=13)	• T: data presented as the ratio T_1/T_2 (geometric mean, 95%CI)<br>- FC (T_1/T_2): 2.0 (1.2-2.35) (*p*-value<0.05)<br>(values estimated from figures)		Calculated CV<br>T_1/T_2: CV= 32.2%		
		↑*PPARBP (MED1)* (white blood cells, n=13)	• T: data presented as the ratio T_1/T_2 (geometric mean, 95%CI)<br>- FC (T_1/T_2): 1.45 (1.2-1.8) (*p*-value<0.05)<br>(values estimated from figures)		Calculated CV<br>T_1/T_2: CV= 44.4%		
Single bioactive compounds							
Poulsen MM et al., 2013 [70]	Res (1500 mg/d, 28 d)	No effect on *PPARGC1A* (skeletal muscle tissue, n=12)	• C: data presented as expression levels (mean, SEM)<br>- Post-: 0.15 (0.021), pre-: 0.12 (0.011)<br>• T: data presented as expression levels (mean, SEM)<br>- Post-: 0.11 (0.017), pre-: 0.13 (0.017)<br>(values estimated from figures)	Medium (data only in figures)	Calculated CV<br>post-C: CV=48.5%<br>pre-C: CV= 31.7%<br>post-T: CV=53.5%<br>pre-T: CV= 45.3%	NR	NR
Olesen J et al., 2014 [71]	Res (250 mg/d, 56 d)	No effect on *PPARGC1A* (skeletal muscle tissue, n=7-14)	• C: data presented as expression levels (mean, SEM)<br>- Post-: 0.96 (0.42), pre-: 1.0 (0.22)<br>• T: data presented as expression levels (mean, SEM)<br>- Post-: 0.92 (0.20), pre-: 1.0 (0.24)<br>(values estimated from figures)	Medium (data only in figures)	Calculated CV<br>post-C: CV=112%<br>pre-C: CV= 58%<br>post-T: CV=65.2%<br>pre-T: CV= 72.0%	NR	NR
	Res + training (56 d)		• C: data presented as expression levels (mean, SEM)<br>- Post-: 1.48 (0.28), pre-: 1.0 (0.24)<br>• T: data presented as expression levels (mean, SEM)<br>- Post-: 1.44 (0.24), pre-: 1.0 (0.14)<br>(values estimated from figures)		Calculated CV<br>post-C: CV=50.0%<br>pre-C: CV= 63.5%<br>post-T: CV=50.0%<br>pre-T: CV= 42.0%		
Yoshino J et al., 2012 [69]	Res (75mg/d, 84 d)	No effect on *PPARGC1A* (skeletal muscle, n=15)	• C: data presented as expression levels (mean, SEM)<br>- Post-: 1.0 (0.10), pre-: 1.1 (0.10)<br>• T: data presented as expression levels (mean, SEM)<br>- Post-: 1.1 (0.10), pre-: 0.97 (0.13)<br>(values estimated from figures)	Medium (data only in figures)	Calculated CV<br>post-C: CV=38.7%<br>pre-C: CV= 35.2%<br>post-T: CV=35.2%<br>pre-T: CV= 51.9%	NR	NR
		No effect on *PPARGC1A* (adipose tissue, n=15)	• C: data presented as expression levels (mean, SEM)<br>- Post-: 1.1 (0.10), pre-: 1.1 (0.10)<br>• T: data presented as expression levels (mean, SEM)<br>- Post-: 1.13 (0.17), pre-: 1.13 (0.10)<br>(values estimated from figures)		Calculated CV<br>post-C: CV=35.2%<br>pre-C: CV= 35.2%<br>post-T: CV=58.3%<br>pre-T: CV= 34.3%		

[1]White blood cells or Leukocytes: mononuclear cells agranulocytes (lymphocytes and monocytes) and polymorphonuclear granulocytes (neutrophils, eosinophils , basophils, mast cells);[2]Data quality based on: absence of proper control group, poor or confusing data reporting, results only in figures;
[3]Coefficient of variation calculated when data (mean, SD, SEM) available.

Table abbreviations (in alphabetical order): C, control group; CI, confidence intervals; CV, coefficient of variation, d, days; DHA, docosahexaenoic acid; EPA, eicosapentaenoic acid; FC, fold-change; *MED1*, mediator complex subunit 1 (alias: *PPARBP*); NR, not reported; post-, after treatment; *PPARA*, peroxisome proliferator activated receptor alpha; *PPARG*, peroxisome proliferator activated receptor gamma; *PPARD*, peroxisome proliferator activated receptor delta; *PPARGC1A,* PPARG coactivator 1 alpha (alias: *PGC1α*); pre-, baseline or before treatment; ref gene, reference gene; Res, resveratrol; SD, standard deviation; SEM, standard error of the mean; T, treated group; VitC, vitamin C; VitE, vitamin E.

Table S6. Summary of the expression changes in the *GPX* family of genes as determined in human cell or tissue samples following intervention with diets, foods or derived products rich in bioactive compounds or with single bioactive compounds. The type and quality of the data presented in the study, estimation of or information about the variability as well as information about the association between the observed effects with the presence of bioactive metabolites and (or) with the corresponding protein quantity/activity are indicated.

Reference	Intervention (daily dose, duration)	Effect potentially associated to the intervention with the bioactive source, (cells[1], tissue, sample size)	*GPXs* expression results in human clinical studies			Association with	
			Data presentation	Data quality[2]	Variability information[3]	Compounds/ Metabolites (urine, plasma, tissue)	Protein levels and (or) activity, tissue
Diets rich in bioactive compounds							
Di Renzo L et al., 2014 [8]	T_1: red wine (250 mL)<br>T_2: Med meal<br>T_3: T_1+T_2<br>T_4: McD meal<br>T_5: T_4+T_1<br>4 h	↑ *GPX1* (blood, n=24)	• C (baseline) *vs* T_1: data presented as ΔCt in bar figures<br>- ΔCt : +0.5 (estimated from figure) (*p*-value: ≤ 0.05)	Poor (confusing data reporting, data only in figures)	No information available	NR	NR
		↑ *GPX1* (blood, n=24)	• C (baseline) *vs* T_3 : data presented as ΔCt in bar figures<br>- ΔCt : +0.6 (estimated from figure) (*p*-value: ≤ 0.05)				
		↑ *GPX1* (blood, n=24)	• C (baseline) *vs* T_5 : data presented as ΔCt in bar figures<br>- ΔCt : +1.5 (estimated from figure) (*p*-value: ≤ 0.05)				
Di Renzo L et al., 2017 [34]	T_1: McD meal+ hazelnuts (40 g)<br>T_2: McD meal<br>3 h	↓ *GPX1* (blood, n=22)	• T_1 : data presented as FC in figure<br>- FC (pre- *vs* post-): >+1.5 (*p*-value:<0.05)<br>• T_2 *vs* T_1 : data presented as FC in figure<br>- FC : >+1.5 (*p*-value:<0.05)	Poor (confusing data reporting, data only in figures)	No information available	NR	NR
		↓ *GPX3* (blood, n=22)	• T_2 : data presented as FC in figure<br>- FC (pre- *vs* post-): <- 1.5 (*p*-value:<0.05)<br>• T_2 *vs* T_1 : data presented as FC in figure<br>- FC: > +1.5 (*p*-value:<0.05)				
		↓*GPX4* (blood, n=22)	• T_2 *vs* T_1 : data presented as FC in figure<br>- FC: > +1.5 (*p*-value:<0.05)				
		↑*GPX7* (blood, n=22)	• T_2 : data presented as FC in figure<br>- FC (pre- *vs* post-): >1.5 (*p*-value:<0.05)<br>• T_2 *vs* T_1 : data presented as FC in figure<br>- FC: <-1.5 (*p*-value:<0.05)				
Grapes and derived products/bioactive compounds							
Weseler AR et al., 2011 [43]	Grape seeds flavanols (200 mg/d, 56 d)	No effect on *GPX1* (blood, n= 28)	• C: NS data presented only in figures (only significantly changed presented as % of change in text)<br>• T: NS data presented only in figures (only significantly changed presented as % of change in text)	Medium – poor (no information on variability)	No information available	NR	NR
		No effect on *GPX4* (blood, n= 28)					

Barona J et al., 2012 [44]	Grape powder (46 g/d, 28 d)	No effect on *GPX1* (mononuclear cells, n= 24)	• T *vs* C : data presented as arbitrary units (mean ± SD)<br>- Dyslipidaemic : 0.460 ± 1.793<br>- Non-dyslipidaemic: −0.152 ± 0.722	Medium-poor (only post- values)	Calculated CV<br>Dyslipidaemic:<br>post-T *vs* post-C :<br>CV= 390%<br>Non-dyslipidaemic<br>post-T *vs* post-C :<br>CV= 475%	NR	NR
		No effect on *GPX4* (mononuclear cells, n= 24)	• T *vs* C : data presented as arbitrary units (mean ± SD)<br>- Dyslipidaemic : 0.196 ± 0.738<br>- Non-dyslipidaemic: -0.196 ± 0.798		Calculated CV<br>Dyslipidaemic:<br>post-T *vs* post-C :<br>CV= 377%<br>Non-dyslipidaemic<br>post-T *vs* post-C :<br>CV= 407%		
**Foods and derived extracts**							
Donadio JLS et al., 2017 [32]	Brazil nuts (Se) (3-4 g (300 µg)/d, 56 d)	↑ ***GPX1*** (blood, n= 130 or n=12, unclear)	• C: not included<br>• T: data presented as relative expression levels, stratification by genotype (CC, CT+TT)<br>• CC : post-: +2.2 (data estimated from figure), pre-: +1.7 (data estimated from figure)<br>- Post- *vs* pre- (↑*p*-value: < 0.05)	Poor (control not included; unclear reporting of individuals per group; data in figures; unclear reporting of the type of data)	No information available	NR	NR
Dragsted LO et al., 2006 [6]	Mix FruVeg (600 g /d, 24 d)	(NS↑) *GPX1* (white blood cells, n=43)	• Two controls, C_1 and C_2 (data presented as time-course of expression in figure, mean ± SEM)<br>• T: (data presented as time-course of expression in figure, mean ± SEM), FC<+1.5	Poor (unclear reporting of tissue sample and of number of subjects per group, data only in figure)	The↑ is mostly due to large changes only in 2 subjects	NR	Erythrocytes: ↑GPx-1 activity in T (post- *vs* pre-) previously reported [7]
Marotta F et al, 2010 [39]	Fermented papaya (6 g /28 d)	↑***GPX1*** (neutrophils, n= 11)	• C: not included<br>• T: data presented as arbitrary expression units (relative expression levels, data estimated from figure)<br>- Post (28d)-: +6.7, post (14 d)-: +6.1 *vs* pre-: +0.1 (*p*-value: < 0.01)	Poor (control group not included, data only in figures)	No information available	NR	NR

Volz N et al., 2012 [36]	Coffee (29.5 g /d, 28 d)	No effect on *GPX1* (lymphocytes, n= 29)	• C: not included<br>• T: data presented as FC<br>- FC (post- *vs* pre-): 0.5-2.2 (individual data presented)	Poor (control group not included)	Calculated CV (based on calculated mean: 1.16 and SD: 0.51)<br>CV: 44 %	NR	NR
Carrera-Quintanar L et al., 2015 [55]	T_1: *Lippia citriodora* extract (1.22 g/d)<br>T_2: Almond beverage + vitC + vitE) (250 mL + 25 mg + 75 mg)/d),<br>T_3: 0.55 g/d T_1 + 250 mL/d T_2,<br>21 d	No effect on *GPX1* (neutrophils, n=33)	• C: placebo, data presented as expression levels (data estimated from figure, mean ± SEM)<br>- Post-: 0.7 *vs* pre-: 1 (arbitrarily referred)<br>• T_1: data presented as expression levels (data estimated from figure, mean ± SEM)<br>- Post-: 0.8 *vs* pre-:1 (arbitrarily referred)<br>• T_2: data presented as expression levels (data estimated from figure, mean ± SEM)<br>- Post-: 1.3 *vs* pre-:1 (arbitrarily referred)<br>• T_3: data presented as expression levels (data estimated from figure, mean ± SEM)<br>- Post-: 1.2 *vs* pre-:1(arbitrarily referred)	Poor (data only in figures, only intragroup comparison)	CV calculated<br>C: CV=164%<br>T_1: CV=71.3%<br>T_2: CV=168%<br>T_3: CV=95.8%	NR	↑ GPx-1 activity in erythrocytes in T_1 *vs* C (post)

[1]**White blood cells or Leukocytes:** mononuclear cells agranulocytes (lymphocytes and monocytes) and polymorphonuclear granulocytes (neutrophils, eosinophils , basophils, mast cells)**;** [2]Data quality based on: absence of proper control group, poor or confusing data reporting, results only in figures; [3]Coefficient of variation calculated when data (mean, SD, SEM) available.

Table abbreviations (in alphabetical order): C, control group; CV, coefficient of variation; d, days; FC, fold-change; *GPX1,* glutathione peroxidase 1; *GPX4*, glutathione peroxidase 4; *GPX7*, glutathione peroxidase 7; h, hours; McD, McDonald; Med, Mediterranean; FruVeg, fruits and vegetables; NR, not reported; NS, not significant; post-, after treatment; pre-, baseline or before treatment; SD, standard deviation; SEM, standard error of the mean; Se, selenium; T, treated group; vitC, vitamin C; vitE, vitamin E.

Supplementary References

1. Persson, I.; He, L.; Fang, C.; Normén, L.; Rylander, R. Influence of vegetables on the expression of GSTP1 in humans-a pilot intervention study (Sweden). *Cancer Causes Control* **2000**, *11*, 359–361.
2. Møller, P.; Vogel, U.; Pedersen, A.; Dragsted, L.O.; Sandström, B.; Loft, S. No effect of 600 grams fruit and vegetables per day on oxidative DNA damage and repair in healthy nonsmokers. *Cancer Epidemiol. Biomarkers Prev.* **2003**, *12*, 1016–1022.
3. Almendingen, K.; Brevik, A.; Nymoen, D.A.; Hilmarsen, H.T.; Andresen, P.A.; Andersen L.F.; Vatn, M. Modulation of COX-2 expression in peripheral blood cells by increased intake of fruit and vegetables? *Eur. J. Clin. Nutr.* **2005**, *59*, 597–602, DOI:10.1038/sj.ejcn.1602110.
4. Brevik, A.; Andersen, L.F.; Karlsen, A.; Trygg, K.U.; Blomhoff, R.; Drevon, C.A. Six carotenoids in plasma used to assess recommended intake of fruits and vegetables in a controlled feeding study. *Eur. J. Clin. Nutr.* **2004**, *58*, 1166–1173, DOI:10.1038/sj.ejcn.1601945.
5. Brevik, A.; Rasmussen, S.E.; Drevon, C.A.; Andersen, L.F. Urinary excretion of flavonoids reflects even small changes in the dietary intake of fruits and vegetables. *Cancer Epidemiol. Biomarkers Prev.* **2004**, *13*, 843–849.
6. Dragsted, L.O.; Krath, B.; Ravn-Haren, G.; Vogel, U.B.; Vinggaard, A.M.; Bo Jensen, P.; Loft, S.; Rasmussen, S.E.; Sandstrom, T.; Pedersen, A. Biological effects of fruit and vegetables. *Proc. Nutr. Soc.* **2006**, *65*, 61–67.
7. Dragsted, L.O.; Pedersen, A.; Hermetter, A.; Basu, S.; Hansen, M.; Haren, G.R.; Kall, M.; Breinholt, V.; Castenmiller, J.J.; Stagsted, J.; et al. The 6-a-day study: Effects of fruit and vegetables on markers of oxidative stress and antioxidative defense in healthy nonsmokers. *Am. J. Clin. Nutr.* **2004**, *79*, 1060–1072.
8. Di Renzo, L.; Carraro, A.; Valente, R.; Iacopino, L.; Colica, C.; De Lorenzo, A. Intake of red wine in different meals modulates oxidized LDL level, oxidative and inflammatory gene expression in healthy people: A randomized crossover trial. *Oxid. Med. Cell. Longev.* **2014**, *2014*, 681318, DOI:10.1155/2014/681318.
9. De Lorenzo, A.; Bernardini, S.; Gualtieri, P.; Cabibbo, A.; Perrone, M.A.; Giambini, I.; Di Renzo, L. Mediterranean meal versus Western meal effects on postprandial ox-LDL, oxidative and inflammatory gene expression in healthy subjects: A randomized controlled trial for nutrigenomic approach in cardiometabolic risk. *Acta Diabetol.* **2017**, *54*, 141–149, DOI:10.1007/s00592-016-0917-2.
10. Marques-Rocha, J.L.; Milagro, F.I.; Mansego, M.L.; Zulet, M.A.; Bressan, J.; Martínez, J.A. Expression of inflammation-related miRNAs in white blood cells from subjects with metabolic syndrome after 8 wk of following a Mediterranean diet-based weight loss program. *Nutrition* **2016**, *32*, 48–55, DOI:10.1016/j.nut.2015.06.008.
11. Zulet, M.A.; Bondia-Pons, I.; Abete, I.; de la Iglesia, R.; Lopez-Legarrea, P.; Forga, L.; Navas-Carretero, S.; Martínez, J.A. The reduction of the metabolyc syndrome in Navarra-Spain study: A multidisciplinary strategy based on chrononutrition and nutritional education, together with dietetic and psychological control. *Nutr. Hosp.* **2011**, *26*, 16–26.
12. Gasper, A.V.; Traka, M.; Bacon, J.R.; Smith, J.A.; Taylor, M.A.; Hawkey, C.J.; Barrett, D.A.; Mithen, R.F. Consuming broccoli does not induce genes associated with xenobiotic metabolism and cell cycle control in human gastric mucosa. *J. Nutr.* **2007**, *137*, 1718–1724, DOI:10.1093/jn/137.7.1718.
13. Gasper, A.V.; Al-Janobi, A.; Smith, J.A.; Bacon, J.R.; Fortun, P.; Atherton, C.; Taylor, M.A.; Hawkey, C.J.; Barrett, D.A.; Mithen, R.F. Glutathione S-transferase M1 polymorphism and metabolism of sulforaphane from standard and high glucosinolate broccoli. *Am. J. Clin. Nutr.* **2005,** *82*, 1283–1291, DOI:10.1093/ajcn/82.6.1283.
14. Riedl, M.A.; Saxon, A.; Diaz-Sanchez, D. Oral sulforaphane increases Phase II antioxidant enzymes in the human upper airway. *Clin. Immunol.* **2009**, *130*, 244–251, DOI:10.1016/j.clim.2008.10.007.
15. Yanaka, A.; Fahey, J.W.; Fukumoto, A.; Nakayama, M.; Inoue, S.; Zhang, S.; Tauchi, M.; Suzuki, H.; Hyodo, I.; Yamamoto, M. Dietary sulforaphane-rich broccoli sprouts reduce colonization and attenuate gastritis in Helicobacter pylori-infected mice and humans. *Cancer Prev. Res.* **2009**, *2*, 353–360, DOI:10.1158/1940-6207.CAPR-08-0192.

16. Riso, P.; Martini, D.; Møller, P.; Loft, S.; Bonacina, G.; Moro, M.; Porrini, M. DNA damage and repair activity after broccoli intake in young healthy smokers. *Mutagenesis* **2010,** *25*, 595–602, DOI:10.1093/mutage/geq045.
17. Atwell, L.L.; Hsu, A.; Wong, C.P.; Stevens, J.F.; Bella, D.; Yu T.W.; Pereira C.B.; Löhr, C.V.; Christensen, J.M.; Dashwood, R.H.; et al. Absorption and chemopreventive targets of sulforaphane in humans following consumption of broccoli sprouts or a myrosinase-treated broccoli sprout extract. *Mol. Nutr. Food Res.* **2015**, *59*, 424–433, DOI:10.1002/mnfr.201400674.
18. Doss, J.F.; Jonassaint, J.C.; Garrett, M.E.; Ashley-Koch, A.E.; Telen M.J.; Chi, J.T. Phase 1 study of a sulforaphane-containing broccoli sprout homogenate for sickle cell disease. *PLoS ONE* **2016**, *11*, e0152895, DOI:10.1371/journal.pone.0152895.
19. Camargo, A.; Ruano, J.; Fernandez, J.M.; Parnell, L.D.; Jimenez, A.; Santos-Gonzalez, M.; Marin, C.; Perez-Martinez, P.; Uceda, M.; Lopez-Miranda, J.; et al. Gene expression changes in mononuclear cells in patients with metabolic syndrome after acute intake of phenol-rich virgin olive oil. *BMC Genomics*. **2010**, *11*, 253, DOI:10.1186/1471-2164-11-253.
20. Konstantinidou, V.; Covas, M.I.; Muñoz-Aguayo, D.; Khymenets, O.; de la Torre, R.; Saez, G.; Tormos Mdel, C.; Toledo, E.; Marti, A.; Ruiz-Gutiérrez, V.; et al. *In vivo* nutrigenomic effects of virgin olive oil polyphenols within the frame of the Mediterranean diet: A randomized controlled trial. *FASEB J.* **2010**, *24*, 2546–2557, DOI:10.1096/fj.09-148452.
21. Chan, J.M.; Weinberg, V.; Magbanua, M.J.; Sosa, E.; Simko, J.; Shinohara, K.; Federman, S.; Mattie, M.; Hughes-Fulford, M.; Haqq, C.; et al. Nutritional supplements, COX-2 and IGF-1 expression in men on active surveillance for prostate cancer. *Cancer Causes Control* **2011**, *22,* 141–150, DOI:10.1007/s10552-010-9684-5.
22. Castañer, O.; Covas, M.I., Khymenets, O.; Nyyssonen, K.; Konstantinidou, V.; Zunft, H.F.; de la Torre, R.; Muñoz-Aguayo, D.; Vila, J.; Fitó, M. Protection of LDL from oxidation by olive oil polyphenols is associated with a downregulation of CD40-ligand expression and its downstream products in vivo in humans. *Am. J. Clin. Nutr.* **2012**, *95*, 1238–1244, DOI:10.3945/ajcn.111.029207.
23. Farràs, M.; Valls, R.M.; Fernández-Castillejo, S.; Giralt, M.; Solà, R.; Subirana, I.; Motilva, M.J.; Konstantinidou, V.; Covas, M.I.; Fitó, M. Olive oil polyphenols enhance the expression of cholesterol efflux related genes in vivo in humans. A randomized controlled trial. *J. Nutr. Biochem.* **2013**, *24*, 1334–1339, DOI:10.1016/j.jnutbio.2012.10.008.
24. Perez-Herrera, A.; Rangel-Zuñiga, O.A.; Delgado-Lista, J.; Marin, C.; Perez-Martinez, P.; Tasset, I.; Tunez, I.; Quintana-Navarro, G.M.; Lopez-Segura, F.; Luque de Castro, M.D.; et al. The antioxidants in oils heated at frying temperature, whether natural or added, could protect against postprandial oxidative stress in obese people. *Food Chem.* **2013,** *138*, 2250–2259, DOI:10.1016/j.foodchem.2012.12.023.
25. Rangel-Zuñiga, O.A.; Haro, C.; Perez-Martinez, P.; Delgado-Lista, J.; Marin, C.; Quintana-Navarro, G.M.; Tinahones, F.J.; Malagón, M.M.; Lopez-Segura, F.; López-Miranda, J.; Perez-Jimenez, F.; Camargo, A. Effect of frying oils on the postprandial endoplasmic reticulum stress in obese people. *Mol. Nutr. Food Res.* **2014**, *58*, 2239–2242, DOI:10.1002/mnfr.201400401.
26. Hernáez, Á.; Remaley, A.T.; Farràs, M.; Fernández-Castillejo, S.; Subirana, I.; Schröder, H.; Fernández-Mampel, M.; Muñoz-Aguayo, D.; Sampson, M.; Solà, R.; et al. Olive oil polyphenols decrease LDL concentrations and LDL atherogenicity in men in a randomized controlled trial. *J. Nutr.* **2015**, *145*, 1692–1697, DOI:10.3945/jn.115.211557.
27. Covas, M.I.; Nyyssönen, K.; Poulsen, H.E.; Kaikkonen, J.; Zunft, H.J.; Kiesewetter, H.; Gaddi, A.; de la Torre R.; Mursu, J.; Bäumler, H.; et al. EUROLIVE study group. The effect of polyphenols in olive oil on heart disease risk factors: a randomized trial. *Ann. Intern. Med.* **2006**, *145,* 333–341.
28. Martín-Peláez, S.; Castañer, O.; Konstantinidou, V.; Subirana, I.; Muñoz-Aguayo, D.; Blanchart, G.; Gaixas, S.; de la Torre, R.; Farré, M.; Sáez, G.T.; et al. Effect of olive oil phenolic compounds on the expression of blood pressure-related genes in healthy individuals. *Eur. J. Nutr.* **2017**, *56*, 663–670, DOI:10.1007/s00394-015-1110-z.
29. Kruse, M.; von Loeffelholz, C.; Hoffmann, D.; Pohlmann, A.; Seltmann, A.C.; Osterhoff, M.; Hornemann, S.; Pivovarova, O.; Rohn, S.; Jahreis, G.; et al. Dietary rapeseed/canola-oil supplementation reduces serum lipids and liver enzymes and alters postprandial inflammatory

responses in adipose tissue compared to olive-oil supplementation in obese men. *Mol. Nutr. Food Res.* **2015**, *59*, 507–519, DOI:10.1002/mnfr.201400446.

30. González-Sarrías, A.; Giménez-Bastida, J.A.; García-Conesa, M.T.; Gómez-Sánchez, M.B.; García-Talavera N.V.; Gil-Izquierdo, A.; Sánchez-Alvarez, C.; Fontana-Compiano, L.O.; Morga-Egea, J.P.; Pastor-Quirante, F.A.; et al. Occurrence of urolithins, gut microbiota ellagic acid metabolites and proliferation markers expression response in the human prostate gland upon consumption of walnuts and pomegranate juice. *Mol. Nutr. Food Res.* **2010**, *54*, 311–322, DOI:10.1002/mnfr.200900152.
31. Hernández-Alonso, P.; Salas-Salvadó, J.; Baldrich-Mora, M.; Juanola-Falgarona, M.; Bulló, M. Beneficial effect of pistachio consumption on glucose metabolism, insulin resistance, inflammation, and related metabolic risk markers: a randomized clinical trial. *Diabetes Care* **2014**, *37*, 3098–3105, DOI:10.2337/dc14-1431.
32. Donadio, J.L.S.; Rogero, M.M.; Cockell, S.; Hesketh, J.; Cozzolino, S.M.F. Influence of genetic variations in selenoprotein genes on the pattern of gene expression after supplementation with Brazil nuts. *Nutrients* **2017**, *9*, E739, DOI:10.3390/nu9070739.
33. Donadio, J.L.S.; Rogero, M.M.; Guerra-Shinohara, E.M.; Desmarchelier, C.; Borel, P.; Cozzolino, S.M.F. SEPP1 polymorphisms modulate serum glucose and lipid response to Brazil nut supplementation. *Eur. J. Nutr.* **2017**, 1–10, DOI:10.1007/s00394-017-1470-7.
34. Di Renzo, L.; Merra, G.; Botta, R.; Gualtieri, P.; Manzo, A.; Perrone, M.A.; Mazza, M.; Cascapera, S.; De Lorenzo, A. Post-prandial effects of hazelnut-enriched high fat meal on LDL oxidative status, oxidative and inflammatory gene expression of healthy subjects: A randomized trial. *Eur. Rev. Med. Pharmacol. Sci.* **2017**, *21*, 1610–1626.
35. Guarrera, S.; Sacerdote, C.; Fiorini, L.; Marsala, R.; Polidoro, S.; Gamberini, S.; Saletta, F.; Malaveille, C.; Talaska, G.; Vineis, P.; et al. Expression of DNA repair and metabolic genes in response to a flavonoid-rich diet. *Br. J. Nutr.* **2007**, *98*, 525–533, DOI:10.1017/S0007114507725151
36. Volz, N.; Boettler, U.; Winkler, S.; Teller, N.; Schwarz, C.; Bakuradze, T.; Eisenbrand, G.; Haupt, L.; Griffiths, L.R.; Stiebitz, H.; et al. Effect of coffee combining green coffee bean constituents with typical roasting products on the Nrf2/ARE pathway in vitro and in vivo. *J. Agric. Food Chem.* **2012**, *60*, 9631–9641, DOI:10.1021/jf302258u.
37. Boettler, U.; Volz, N.; Teller, N.; Haupt, L.M.; Bakuradze, T.; Eisenbrand, G.; Bytof, G.; Lantz, I.; Griffiths, L.R.; Marko, D. Induction of antioxidative Nrf2 gene transcription by coffee in humans: depending on genotype? *Mol. Biol. Rep.* **2012**, *39*, 7155–7162, DOI:10.1007/s11033-012-1547-6.
38. de Pascual-Teresa, S.; Johnston, K.L.; DuPont, M.S.; O'Leary, K.A.; Needs, P.W.; Morgan, L.M.; Clifford, M.N.; Bao, Y.; Williamson, G. Quercetin metabolites downregulate cyclooxygenase-2 transcription in human lymphocytes ex vivo but not in vivo. *J. Nutr.* **2004**, *134*, 552–557, DOI:10.1093/jn/134.3.552.
39. Marotta, F.; Koike, K.; Lorenzetti, A.; Jain, S.; Signorelli, P.; Metugriachuk, Y.; Mantello, P.; Locorotondo, N. Regulating redox balance gene expression in healthy individuals by nutraceuticals: A pilot study. *Rejuvenation Res.* **2010**, *13*, 175–178, DOI:10.1089/rej.2009.0950.
40. Bertuccelli, G.; Zerbinati, N.; Marcellino, M.; Nanda Kumar, N.S.; He, F.; Tsepakolenko, V.; Cervi, J.; Lorenzetti, A.; Marotta, F. Effect of a quality-controlled fermented nutraceutical on skin aging markers: An antioxidant-control, double-blind study. *Exp. Ther. Med.* **2016**, *11*, 909–916, DOI:10.3892/etm.2016.3011.
41. Ishikawa, H.; Goto, M.; Matsuura, N.; Murakami, Y.; Goto, C.; Sakai, T.; Kanazawa, K. A pilot, randomized, placebo-controlled, double-blind phase 0/biomarker study on effect of artepillin C-rich extract of Brazilian propolis in frequent colorectal adenoma polyp patients. *J. Am. Coll. Nutr.* **2012**, *31*, 327–337.
42. Nguyen, A.V.; Martinez, M.; Stamos, M.J.; Moyer, M.P.; Planutis, K.; Hope, C.; Holcombe, R.F. Results of a phase I pilot clinical trial examining the effect of plant-derived resveratrol and grape powder on Wnt pathway target gene expression in colonic mucosa and colon cancer. *Cancer Manag. Res.* **2009**, *1*, 25–37.
43. Weseler, A.R.; Ruijters E.J.; Drittij-Reijnders, M.J.; Reesink, K.D.; Haenen, G.R.; Bast, A. Pleiotropic benefit of monomeric and oligomeric flavanols on vascular health—a randomized controlled clinical pilot study. *PLoS ONE* **2011**, *6*, e28460, DOI:10.1371/journal.pone.0028460.

44. Barona, J.; Blesso, C.N.; Andersen, C.J.; Park, Y.; Lee, J.; Fernandez, M.L. Grape consumption increases anti-inflammatory markers and upregulates peripheral nitric oxide synthase in the absence of dyslipidemias in men with metabolic syndrome. *Nutrients* **2012**, *4*, 1945–1957, DOI:10.3390/nu4121945.
45. Tomé-Carneiro, J.; Larrosa, M.; Yáñez-Gascón, M.J.; Dávalos, A.; Gil-Zamorano, J.; Gonzálvez, M.; García-Almagro, F.J.; Ruiz Ros, J.A.; Tomás-Barberán, F.A.; Espín, J.C.; et al. One-year supplementation with a grape extract containing resveratrol modulates inflammatory-related microRNAs and cytokines expression in peripheral blood mononuclear cells of type 2 diabetes and hypertensive patients with coronary artery disease. *Pharmacol. Res*. **2013**, *72*, 69–82, DOI:10.1016/j.phrs.2013.03.011.
46. Mallery, S.R.; Zwick, J.C.; Pei, P.; Tong, M.; Larsen, P.E.; Shumway, B.S.; Lu, B.; Fields, H.W.; Mumper, R.J.; Stoner, G.D. Topical application of a bioadhesive black raspberry gel modulates gene expression and reduces cyclooxygenase 2 protein in human premalignant oral lesions. *Cancer Res*. **2008**, *68*, 4945–4957, DOI:10.1158/0008-5472.CAN-08-0568.
47. Kropat, C.; Mueller, D.; Boettler, U.; Zimmermann, K.; Heiss, E.H.; Dirsch, V.M.; Rogoll, D.; Melcher, R.; Richling, E.; Marko, D. Modulation of Nrf2-dependent gene transcription by bilberry anthocyanins in vivo. *Mol. Nutr. Food Res*. **2013**, *57*, 545–550, DOI:10.1002/mnfr.201200504.
48. Knobloch, T.J.; Uhrig, L.K.; Pearl, D.K.; Casto, B.C.; Warner, B.M.; Clinton, S.K.; Sardo-Molmenti, C.L.; Ferguson, J.M.; Daly, B.T.; Riedl, K.; et al. Suppression of proinflammatory and prosurvival biomarkers in oral cancer patients consuming ablack raspberry phytochemical-rich troche. *Cancer Prev. Res.* **2016**, *9*, 159–171, DOI:10.1158/1940-6207.CAPR-15-0187.
49. Xie, L.; Vance, T.; Kim, B.; Lee, S.G.; Caceres, C.; Wang, Y.; Hubert, P.A.; Lee, J.Y.; Chun, O.K.; Bolling, B.W. Aronia berry polyphenol consumption reduces plasma total and low-density lipoprotein cholesterol in former smokers without lowering biomarkers of inflammation and oxidative stress: a randomized controlled trial. *Nutr. Res.* **2017**, *37*, 67–77, DOI:10.1016/j.nutres.2016.12.007.
50. Nuñez-Sánchez, M.A.; González-Sarrías, A.; García-Villalba, R.; Monedero-Saiz, T.; García-Talavera, N.V.; Gómez-Sánchez, M.B.; Sánchez-Álvarez, C.; García-Albert, A.M.; Rodríguez-Gil, F.J.; Ruiz-Marín, M.; et al. Gene expression changes in colon tissues from colorectal cancer patients following the intake of an ellagitannin-containing pomegranate extract: a randomized clinical trial. *J. Nutr. Biochem*. **2017**, *42*, 126–133, DOI:10.1016/j.jnutbio.2017.01.014.
51. Knott, A.; Reuschlein, K.; Mielke, H.; Wensorra, U.; Mummert, C.; Koop, U.; Kausch, M.; Kolbe, L.; Peters, N.; Stäb, F.; et al. Natural *Arctium lappa* fruit extract improves the clinical signs of aging skin. *J. Cosmet. Dermatol.* **2008**, *7*, 281–289, DOI:10.1111/j.1473-2165.2008.00407.x.
52. Shrestha, S.; Freake, H.C.; McGrane, M.M.; Volek, J.S.; Fernandez, M.L. A combination of psyllium and plant sterols alters lipoprotein metabolism in hypercholesterolemic subjects by modifying the intravascular processing of lipoproteins and increasing LDL uptake. *J. Nutr*. **2007**, *137*, 1165–1170, DOI:10.1093/jn/137.5.1165.
53. Ghanim, H.; Sia, C.L.; Abuaysheh, S.; Korzeniewski, K.; Patnaik, P.; Marumganti, A.; Chaudhuri, A.; Dandona, P. An antiinflammatory and reactive oxygen species suppressive effects of an extract of Polygonum cuspidatum containing resveratrol. *J. Clin. Endocrinol. Metab.* **2010**, *95*, E1–E8, DOI:10.1210/jc.2010-0482.
54. Marini, A.; Grether-Beck, S.; Jaenicke, T.; Weber, M.; Burki, C.; Formann, P.; Brenden, H.; Schönlau, F.; Krutmann, J. Pycnogenol® effects on skin elasticity and hydration coincide with increased gene expressions of collagen type I and hyaluronic acid synthase in women. *Skin Pharmacol. Physiol*. **2012**, *25*, 86–92, DOI:10.1159/000335261.
55. Carrera-Quintanar, L.; Funes, L.; Vicente-Salar, N.; Blasco-Lafarga, C.; Pons, A.; Micol, V.; Roche, E. Effect of polyphenol supplements on redox status of blood cells: A randomized controlled exercise training trial. *Eur. J. Nutr*. **2015**, *54,* 1081–1093, DOI:10.1007/s00394-014-0785-x.
56. Turowski, J.B.; Pietrofesa, R.A.; Lawson, J.A.; Christofidou-Solomidou, M.; Hadjiliadis, D. Flaxseed modulates inflammatory and oxidative stress biomarkers in cystic fibrosis: A pilot study. *BMC Complement. Altern. Med*. **2015**, *15*, 148, DOI:10.1186/s12906-015-0651-2.

57. Plat, J.; Mensink, R.P. Effects of plant stanol esters on LDL receptor protein expression and on LDL receptor and HMG-CoA reductase mRNA expression in mononuclear blood cells of healthy men and women. *FASEB J.* **2002**, *16*,258–260, DOI:10.1096/fj.01-0653fje.
58. Crespo, M.C.; Tomé-Carneiro, J.; Burgos-Ramos, E.; Loria Kohen, V.; Espinosa, M.I.; Herranz, J.; Visioli, F. One-week administration of hydroxytyrosol to humans does not activate Phase II enzymes. *Pharmacol. Res.* **2015**, *95–96*,132–137, DOI:10.1016/j.phrs.2015.03.018.
59. Boss, A.; Kao, C.H.; Murray, P.M.; Marlow, G.; Barnett, M.P.; Ferguson, L.R. Human intervention study to assess the effects of supplementation with olive leaf extract on peripheral blood mononuclear cell gene expression. *Int. J. Mol Sci.* **2016**, *17*, E2019, DOI:10.3390/ijms17122019.
60. Daak, A.A.; Elderdery, A.Y.; Elbashir, L.M.; Mariniello, K.; Mills, J.; Scarlett, G.; Elbashir, M.I.; Ghebremeskel, K. Omega 3 (*n*-3) fatty acids down-regulate nuclear factor-kappa B (NF-KB) gene and blood cell adhesion molecule expression in patients with homozygous sickle cell disease. *Blood Cells Mol. Dis.* **2015**, *55*, 48–55, DOI:10.1016/j.bcmd.2015.03.014.
61. Labonté, M.È.; Couture, P.; Tremblay, A.J.; Hogue, J.C.; Lemelin, V.; Lamarche, B. Eicosapentaenoic and docosahexaenoic acid supplementation and inflammatory gene expression in the duodenum of obese patients with type 2 diabetes. *Nutr. J.* **2013**, *12*, 98, DOI:10.1186/1475-2891-12-98.
62. Jamilian, M.; Samimi, M.; Mirhosseini, N.; Afshar Ebrahimi, F.; Aghadavod, E.; Taghizadeh, M.; Asemi, Z. A randomized double-blinded, placebo-controlled trial investigating the effect of fish oil supplementation on gene expression related to insulin action, blood lipids, and inflammation in gestational diabetes mellitus-fish oil supplementation and gestational diabetes. *Nutrients* **2018**, *10*, E163, DOI:10.3390/nu10020163.
63. Marini, A.; Jaenicke, T.; Grether-Beck, S.; Le Floc'h, C.; Cheniti, A.; Piccardi, N.; Krutmann, J. Prevention of polymorphic light eruption by oral administration of a nutritional supplement containing lycopene, β-carotene, and *Lactobacillus johnsonii*: Results from a randomized, placebo-controlled, double-blinded study. *Photodermatol. Photoimmunol. Photomed.* **2014**, *30*, 189–194, DOI:10.1111/phpp.12093.
64. Farris, P.; Yatskayer, M.; Chen, N.; Krol, Y.; Oresajo, C. Evaluation of efficacy and tolerance of a nighttime topical antioxidant containing resveratrol, baicalin, and vitamin E for treatment of mild to moderately photodamaged skin. *J. Drugs Dermatol*. **2014**, *13*, 1467–1472.
65. Radler, U.; Stangl, H.; Lechner, S.; Lienbacher, G.; Krepp, R.; Zeller, E.; Brachinger, M.; Eller-Berndl, D.; Fischer, A.; Anzur, C.; et al. A combination of (ω-3) polyunsaturated fatty acids, polyphenols and L-carnitine reduces the plasma lipid levels and increases the expression of genes involved in fatty acid oxidation in human peripheral blood mononuclear cells and HepG2 cells. *Ann. Nutr. Metab.* **2011**, *58*, 133–140, DOI:10.1159/000327150.
66. Frommel, T.O.; Lietz, H.; Mobarhan, S. Expression of mRNA for the gap-junctional protein connexin43 in human colonic tissue is variable in response to beta-carotene supplementation. *Nutr. Cancer* **1994**, *22*, 257–265.
67. Vors, C.; Allaire, J.; Marin, J.; Lépine, M.C.; Charest, A.; Tchernof, A.; Couture, P.; Lamarche, B. Inflammatory gene expression in whole blood cells after EPA vs. DHA supplementation: Results from the ComparED study. *Atherosclerosis* **2017**, *257*, 116–122, DOI:10.1016/j.atherosclerosis.2017.01.025.
68. Allaire, J.; Couture, P.; Leclerc, M.; Charest, A.; Marin, J.; Lépine, M.C.; Talbot, D.; Tchernof, A.; Lamarche, B. A randomized, crossover, head-to-head comparison of eicosapentaenoic acid and docosahexaenoic acid supplementation to reduce inflammation markers in men and women: The Comparing EPA to DHA (ComparED) Study. *Am. J. Clin. Nutr.* **2016**, *104*, 280–287, DOI:10.3945/ajcn.116.131896.
69. Yoshino, J.; Conte, C.; Fontana, L.; Mittendorfer, B.; Imai, S.; Schechtman, K.B.; Gu, C.; Kunz, I.; Rossi Fanelli, F.; Patterson, B.W.; Klein, S. Resveratrol supplementation does not improve metabolic function in nonobese women with normal glucose tolerance. *Cell Metab.* **2012**, *16*, 658–664, DOI:10.1016/j.cmet.2012.09.015.
70. Poulsen, M.M.; Vestergaard, P.F.; Clasen, B.F.; Radko, Y.; Christensen, L.P.; Stødkilde-Jørgensen, H.; Møller, N.; Jessen, N.; Pedersen, S.B.; Jørgensen J.O. High-dose resveratrol supplementation in obese

men: An investigator-initiated, randomized, placebo-controlled clinical trial of substrate metabolism, insulin sensitivity, and body composition. *Diabetes* **2013**, *62*, 1186–1195, DOI:10.2337/db12-0975.

71. Olesen, J.; Gliemann, L.; Biensø, R.; Schmidt, J.; Hellsten, Y.; Pilegaard, H. Exercise training, but not resveratrol, improves metabolic and inflammatory status in skeletal muscle of aged men. *J. Physiol.* **2014**, *592*, 1873–1886, DOI:10.1113/jphysiol.2013.270256.
72. Chachay, V.S.; Macdonald, G.A.; Martin, J.H.; Whitehead, J.P.; O'Moore-Sullivan, T.M.; Lee ,P.; Franklin, M.; Klein, K.; Taylor, P.J.; Ferguson, M.; et al. Resveratrol does not benefit patients with nonalcoholic fatty liver disease. *Clin. Gastroenterol. Hepatol*. **2014**, *12*, 2092–2103, DOI:10.1016/j.cgh.2014.02.024.
73. Yiu, E.M.; Tai, G.; Peverill, R.E.; Lee, K.J.; Croft, K.D.; Mori, T.A.; Scheiber-Mojdehkar, B.; Sturm, B.; Praschberger, M.; Vogel, A.P.; et al. An open-label trial in Friedreich ataxia suggests clinical benefit with high-dose resveratrol, without effect on frataxin levels. *J. Neurol*. **2015**, *262*, 1344–1353, DOI:10.1007/s00415-015-7719-2.
74. Mansur, A.P.; Roggerio, A.; Goes, M.F.S.; Avakian, S.D.; Leal, D.P.; Maranhão, R.C.; Strunz, C.M.C. Serum concentrations and gene expression of sirtuin 1 in healthy and slightly overweight subjects after caloric restriction or resveratrol supplementation: A randomized trial. *Int. J. Cardiol.* **2017,** *227*, 788–794, DOI:10.1016/j.ijcard.2016.10.058.
75. Morrow, D.M.; Fitzsimmons, P.E.; Chopra, M.; McGlynn, H. Dietary supplementation with the anti-tumour promoter quercetin: Its effects on matrix metalloproteinase gene regulation. *Mutat. Res*. **2001,** *480–481*, 269–276.
76. Nieman, D.C.; Henson, D.A.; Davis, J.M.; Angela Murphy, E.; Jenkins, D.P.; Gross, S.J.; Carmichael, M.D.; Quindry, J.C.; Dumke, C.L.; Utter, A.C.; et al. Quercetin's influence on exercise-induced changes in plasma cytokines and muscle and leukocyte cytokine mRNA. *J. Appl. Physiol*. **2007**, *103*, 1728–1735, DOI:10.1152/japplphysiol.00707.2007.
77. Boesch-Saadatmandi, C.; Pospissil, R.T.; Graeser, A.C.; Canali, R.; Boomgaarden, I.; Doering, F.; Wolffram, S.; Egert, S.; Mueller, M.J.; Rimbach, G. Effect of quercetin on paraoxonase 2 levels in RAW264.7 macrophages and in human monocytes—Role of quercetin metabolism. *Int. J. Mol. Sci.* **2009**, *10*, 4168–4177, DOI:10.3390/ijms10094168.
78. Nelson, D.M.; Joseph, B.; Hillion, J.; Segal, J.; Karp, J.E.; Resar, L.M. Flavopiridol induces BCL-2 expression and represses oncogenic transcription factors in leukemic blasts from adults with refractory acute myeloid leukemia. *Leuk. Lymphoma* **2011**, *52*, 1999–2006, DOI:10.3109/10428194.2011.591012.
79. Lazarevic, B.; Hammarström, C.; Yang, J.; Ramberg, H.; Diep, L.M.; Karlsen, S.J.; Kucuk, O.; Saatcioglu, F, Taskèn K.A.; Svindland A. The effects of short-term genistein intervention on prostate biomarker expression in patients with localised prostate cancer before radical prostatectomy. *Br. J. Nutr*. **2012**, *108,* 2138–2147, DOI:10.1017/S0007114512000384.
80. Kerksick, C.M.; Roberts, M.D.; Dalbo, V.J.; Kreider, R.B.; Willoughby, D.S. Changes in skeletal muscle proteolytic gene expression after prophylactic supplementation of EGCG and NAC and eccentric damage. *Food Chem. Toxicol*. **2013**, *61*, 47–52, DOI:10.1016/j.fct.2013.01.026.
81. Most, J.; van Can, J.G.; van Dijk, J.W.; Goossens, G.H.; Jocken, J.; Hospers, J.J.; Bendik, I.; Blaak, E.E. A 3-day EGCG-supplementation reduces interstitial lactate concentration in skeletal muscle of overweight subjects. *Sci. Rep*. **2015**, *5*, 17896, DOI:10.1038/srep17896.
82. Koosirirat, C.; Linpisarn, S.; Changsom, D.; Chawansuntati, K.; Wipasa, J. Investigation of the anti-inflammatory effect of *Curcuma longa* in *Helicobacter pylori*-infected patients. *Int. Immunopharmacol.* **2010,** *10*, 815–818, DOI:10.1016/j.intimp.2010.04.021.
83. Klickovic, U.; Doberer, D.; Gouya, G.; Aschauer, S.; Weisshaar, S.; Storka, A.; Bilban, M.; Wolzt, M. Human pharmacokinetics of high dose oral curcumin and its effect on heme oxygenase-1 expression in healthy male subjects. *Biomed Res. Int*. **2014**, *2014*, 458592, DOI:10.1155/2014/458592.
84. Vogel, U.; Møller, P.; Dragsted, L.; Loft, S.; Pedersen, A.; Sandstrom, B. Inter-individual variation, seasonal variation and close correlation of OGG1 and ERCC1 mRNA levels in full blood from healthy volunteers. *Carcinogenesis* **2002,** *23*, 1505–1509.

85. Böyum, A. Isolation of mononuclear cells and granulocytes from human blood. Isolation of monuclear cells by one centrifugation, and of granulocytes by combining centrifugation and sedimentation at 1 g. *Scand. J. Clin. Lab. Invest. Suppl.* **1968**, *97*, 77–89.
86. Chomczynski, P.; Sacchi, N. Single-step method of RNA isolation by acid guanidinium thiocyanate-phenolchloroform extraction. *Anal. Biochem.* **1987,** *162*, 156–159.
87. Park, Y.K.; Rasmussen, H.E.; Ehlers, S.J.; Blobaum, K.R.; Lu, F.; Schlegal, V.L.; Carr, T.P.; Lee, J.Y. Repression of pro-inflammatory gene expression by lipid extract of *Nostoc commune* var sphaeroides Kutzing, a blue-green alga, *via* inhibition of nuclear factor-kappa-B in RAW 264.7 macrophages. *Nutr. Res.* **2008**, *28*, 83–91, DOI:10.1016/j.nutres.2007.11.008.
88. Böyum, A. Separation of white blood cells. *Nature* **1964**, *204*, 793–794.
89. GeneCards. Available online: http://www.genecards.org/.
